# Effect of the Histone Deacetylase Inhibitor FRM-0334 on Progranulin Levels in Patients With Progranulin Gene Haploinsufficiency: A Randomized Clinical Trial

**DOI:** 10.1001/jamanetworkopen.2021.25584

**Published:** 2021-09-24

**Authors:** Peter A. Ljubenkov, Lauren Edwards, Leonardo Iaccarino, Renaud La Joie, Julio C. Rojas, Mary Koestler, Baruch Harris, Bradley F. Boeve, Barbara Borroni, John C. van Swieten, Murray Grossman, Florence Pasquier, Giovanni B. Frisoni, Catherine J. Mummery, Rik Vandenberghe, Isabelle Le Ber, Didier Hannequin, Scott M. McGinnis, Sophie Auriacombe, Marco Onofrj, Ira J. Goodman, Henry J. Riordan, Gary Wisniewski, Jacob Hesterman, Ken Marek, Beth Ann Haynes, Holger Patzke, Gerhard Koenig, Dana Hilt, Hans Moebius, Adam L. Boxer

**Affiliations:** 1 Memory and Aging Center, Department of Neurology, University of California at San Francisco, Weill Institute for Neurosciences, San Francisco; 2 ROME Therapeutics, Cambridge, Massachusetts; 3 Metera Pharmaceuticals, Cambridge, Massachusetts; 4 Mayo Clinic, Rochester, Minnesota; 5 Neurology Unit, Centre for Neurodegenerative Disorders, ASST Spedali Civili Brescia, Italy; 6 Department of Clinical and Experimental Sciences, University of Brescia, Italy; 7 Department of Neurology, Erasmus University Medical Center, Rotterdam, the Netherlands; 8 Perelman School of Medicine, University of Pennsylvania, Philadelphia; 9 Lille University, Inserm 1172, CHU Lille, CNR-MAJ, DISTALZ, LiCEND, Lille, France; 10 Lab of Alzheimer's Neuroimaging and Epidemiology, IRCCS Istituto Centro San Giovanni di Dio Fatebenefratelli, Brescia, Italy; 11 Dementia Research Centre, Institute of Neurology, University College London, London, United Kingdom; 12 Neurology Service University Hospitals Leuven, Leuven, Belgium; 13 Laboratory for Cognitive Neurology, Department of Neurosciences, Leuven Brain Institute, Leuven, Belgium; 14 Paris Brain Institute, Sorbonne University, Paris, France; 15 Neurology Department, Reference Centre for Rare or Early Dementias, Paris, France; 16 CHU Rouen Normandie, Service de Neurologie, Rouen University Hospital, Rouen, France; 17 Department of Neurology, Massachusetts General Hospital, Charlestown; 18 Neurology Department, CHU Bordeaux, Bordeaux Hospital, Bordeaux, France; 19 Department of Neuroscience Imaging, University G d'Annunzio of Chieti-Pescara, Chieti, Italy; 20 Compass Research, Bioclinica, Orlando, Florida; 21 Worldwide Clinical Trials, King of Prussia, Pennsylvania; 22 Invicro, A Konica Minolta Company, Boston, Massachusetts; 23 Institute for Neurodegenerative Disorders, New Haven, Connecticut; 24 FORUM Pharmaceuticals Incorporated, Waltham, Massachusetts; 25 Alector, South San Francisco, California; 26 Voyager Therapeutics, Watertown, Massachusetts; 27 Arkuda Therapeutics, Watertown, Massachusetts; 28 Lysosomal Therapeutics, Cambridge, Massachusetts; 29 Frequency Therapeutics, Farmington, Connecticut; 30 Athira Pharma Inc, Seattle, Washington

## Abstract

**Importance:**

Histone deacetylase inhibitors have been repeatedly shown to elevate progranulin levels in preclinical models. This report describes the first randomized clinical trial of a histone deacetylase inhibitor in frontotemporal dementia (FTD) resulting from progranulin (*GRN*) gene variations.

**Objective:**

To characterize the safety, tolerability, plasma pharmacokinetics, and pharmacodynamic effects of oral FRM-0334 on plasma progranulin and other exploratory biomarkers, including fluorodeoxyglucose (FDG)–positron emission tomography (PET), in individuals with *GRN* haploinsufficiency.

**Design, Setting, and Participants:**

In this randomized, double-blind, placebo-controlled, dose-escalating, phase 2a safety, tolerability, and pharmacodynamic clinical study, 2 doses of a histone deacetylase inhibitor (FRM-0334) were administered to participants with prodromal to moderate FTD with granulin variations. Participants were recruited from January 13, 2015, to April 13, 2016. The study included 27 participants with prodromal (n = 8) or mild-to-moderate symptoms of FTD (n = 19) and heterozygous pathogenic variations in *GRN* and was conducted at multiple centers in North America, the UK, and the European Union. Data were analyzed from June 9, 2019, to May 13, 2021.

**Interventions:**

Daily oral placebo (n = 5), 300 mg of FRM-0334 (n = 11), or 500 mg of FRM-0334 (n = 11) was administered for 28 days.

**Main Outcomes and Measures:**

Primary outcomes were safety and tolerability of FRM-0334 and its peripheral pharmacodynamic effect on plasma progranulin. Secondary outcomes were the plasma pharmacokinetic profile of FRM-0334 and its pharmacodynamic effect on cerebrospinal fluid progranulin. Exploratory outcomes were FDG-PET, FTD clinical severity, and cerebrospinal fluid biomarkers (neurofilament light chain [NfL], amyloid β 1-42, phosphorylated tau 181, and total tau [t-tau]).

**Results:**

A total of 27 participants (mean [SD] age, 56.6 [10.5] years; 16 women [59.3%]; 26 White participants [96.3%]) with *GRN* variations were randomized and completed treatment. FRM-0334 was safe and well tolerated but did not affect plasma progranulin (4.3 pg/mL per day change after treatment; 95% CI, –10.1 to 18.8 pg/mL; *P* = .56), cerebrospinal fluid progranulin (0.42 pg/mL per day; 95% CI, –0.12 to 0.95 pg/mL; *P* = .13), or exploratory pharmacodynamic measures. Plasma FRM-0334 exposure did not increase proportionally with dose. Brain FDG-PET data were available in 26 of 27 randomized participants. In a cross-sectional analysis of 26 individuals, bifrontal cortical FDG hypometabolism was associated with worse Clinical Dementia Rating (CDR) plus National Alzheimer’s Coordinating Center frontotemporal lobar degeneration sum of boxes score (b = −3.6 × 10^−2^ standardized uptake value ratio [SUVR] units/CDR units; 95% CI, −4.9 × 10^−2^ to −2.2 × 10^−2^; *P* < .001), high cerebrospinal fluid NfL (b = −9.2 × 10^−5^ SUVR units/pg NfL/mL; 95% CI, −1.3 × 10^−4^ to −5.6 × 10^−5^; *P* < .001), and high CSF t-tau (−7.2 × 10^−4^ SUVR units/pg t-tau/mL; 95% CI, −1.4 × 10^−3^ to −9.5 × 10^−5^; *P* = .03).

**Conclusions and Relevance:**

In this randomized clinical trial, the current formulation of FRM-0334 did not elevate PRGN levels, which could reflect a lack of efficacy at attained exposures, low bioavailability, or some combination of the 2 factors. Bifrontal FDG-PET is a sensitive measure of symptomatic *GRN* haploinsufficiency. International multicenter clinical trials of FTD-GRN are feasible.

**Trial Registration:**

ClinicalTrials.gov Identifier: NCT02149160

## Key Points

**Question:**

What is the safety, tolerability, pharmacodynamic, and pharmacokinetic profile of the histone deacetylase inhibitor FRM-0334 in participants with progranulin gene (*GRN*) haploinsufficiency?

**Findings:**

In this randomized placebo-controlled clinical trial including 27 participants with *GRN* haploinsufficiency, FRM-0334 was safe and well tolerated; however, it failed to increase plasma progranulin and cerebrospinal fluid progranulin and failed to demonstrate dose-dependent oral bioavailability.

**Meaning:**

The studied formulation of FRM-0334 should not be investigated in future clinical trials enrolling participants with *GRN* haploinsufficiency; this study did not fully address the potential of histone deacetylase inhibition to alter *GRN* expression.

## Teaser

This randomized clinical trial investigates the pharmacokinetic and pharmacodynamic profile, tolerability, and safety of the histone deacetylase inhibitor FRM-0334 in people with progranulin gene haploinsufficiency.

## Introduction

Autosomal dominant variations in the progranulin (PGRN) gene *GRN* (OMIM 138945)^1,2,3,4,5,6^ are among the most common causes of genetic frontotemporal degeneration (FTD).^7^ Pathogenic *GRN* variations result in haploinsufficiency,^7,8,9,10^ with a variable, roughly 50% reduction in secreted PGRN protein in the plasma^11,12^ and cerebrospinal fluid (CSF).^11^ The potential to restore or replace PGRN has spurred the development of a number of new *GRN* therapies that are entering human clinical trials, with 1 agent having recently entered a phase 3 trial.^13^ Clinical development programs have been aided by the ability to use blood and CSF PGRN concentrations as pharmacodynamics biomarkers in *GRN* haploinsufficiency clinical trials. However, the rarity of trial-ready participants and lack of established end points are barriers to successful development of new therapies.

The histone deacetylase (HDAC) inhibitor FRM-0334, formerly EVP-0334, was designed as a potent, orally available drug with brain-to-plasma concentration ratios of 3 to 6 in rodents.^14^ In preclinical studies, a similar HDAC inhibitor, suberoylanilide hydroxamic acid, enhanced PGRN transcription to near-normal levels in human-derived *GRN*^-/+^(R493X) cells (−/+ denotes that the cells have heterozygous loss of function of the *GRN* gene) and prevented cytosolic TAR DNA-binding protein 43 accumulation in PGRN-deficient lymphoblasts.^15,16^ Although suberoylanilide hydroxamic acid is approved for treatment of cutaneous lymphoma,^17^ its poor central nervous system availability^18^ prioritized the exploration of FRM-0334 in *GRN* haploinsufficiency. In subsequent unpublished preclinical studies, FRM-0334 boosted *GRN* mRNA and PGRN in a dose-dependent manner in human *GRN^-/+^* lymphoblasts and in the cortex of mice (dosed with oral FRM-0334). Additionally, single and multiple doses for FRM-0334, ranging from 10 mg to 400 mg daily, were safe, well tolerated, and associated with dose-proportional plasma exposure in an unpublished, phase 1, placebo-controlled clinical trial of FRM-0334 in 87 healthy volunteers aged 18 to 65 years. We investigated the safety, tolerability, and plasma pharmacodynamic (PD) effects (change in PGRN) of FRM-0334 in a multinational clinical trial completed in PRGN *GRN* haploinsufficiency. We also characterized the peripheral pharmacokinetic (PK) profile and central PD effects (change in CSF PGRN) of FRM-0334 in *GRN* haploinsufficiency. Additionally, this trial’s inclusion of a variety of exploratory PD end points allowed for novel analyses comparing cortical fluorodeoxyglucose positron emission tomography (FDG-PET), clinical measures of functional FTD severity, and candidate CSF biomarkers of neuronal injury, including neurofilament light chain (NfL), amyloid β 1-42 (Aβ_1-42_), phosphorylated tau 181 (p-tau_181_), and total tau (t-tau). We present data from the first trial implementing an international, multisite protocol as a mechanism for patient recruitment in the rare familial FTD space.

## Methods

### Participants

This randomized clinical trial included participants aged 27 to 75 years with Clinical Laboratory Improvement Amendments–confirmed pathogenic *GRN* variations, including prodromal and mild-to-moderate (Clinical Dementia Rating [CDR] plus National Alzheimer’s Coordinating Center [NACC] frontotemporal lobar degeneration [FTLD] behavior and language domain sum of boxes <16)^19,20^ FTD stages. Race and ethnicity information was recorded; participants self-identified their race and ethnicity. All participants were recruited and randomized from January 13, 2015, to April 13, 2016, at 11 clinical sites in the US, UK, France, Italy, the Netherlands, and Belgium (trial protocol in Supplement 1; eTable 1 in Supplement 2). Significant comorbid thyroid, hematologic, kidney, cardiovascular, inflammatory, or hepatic disease was exclusionary. The trial protocol specified that comorbid neurologic or psychiatric disease (if determined to be unrelated to *GRN* variations) was also exclusionary, though these criteria were not cited as the basis for exclusion of any participants recruited for screening (eFigure in Supplement 2). Ethics approval was obtained at each site from the local institutional review board and independent ethics committee. Written informed consent (from participants and legal proxies) was obtained at the start of recruitment, in compliance with the current revision of the Declaration of Helsinki, current International Conference on Harmonisation and Good Clinical Practice guidelines, the Consolidated Standards of Reporting Trials (CONSORT) reporting guideline, and local regulations.

### General Procedures

Participants and site staff were blinded to treatment assignment. A central Interactive Voice/Web Response System^21^ generated randomization assignments in 2 sequential cohorts. Cohort 1 was randomized to placebo or low-dose (300 mg daily) FRM-0334. Cohort 2 began after review of cohort 1 safety data and included randomization to placebo or high-dose (500 mg daily) FRM-0334. Overall, 5 patients were randomized to placebo, 11 to low-dose FRM-0334, and 11 to high-dose FRM-0334. The study drug (FRM-0334 drug substance blended with sodium lauryl sulfate, microcrystalline cellulose, magnesium stearate, and colloidal silicon dioxide) and placebo (containing microcrystalline cellulose) were presented as identical, opaque, white gelatin capsules and taken daily for 28 days. A central pharmacy was used to distribute packaged drugs to blinded site staff. Plasma collection occurred at screening visit 1 (8-30 days before the first dose), at screening visit 2 (1-7 days before the first dose), on study day 1 (first day of dose), in weekly intervals during dosing (days 7, 14, 21, and 28), and at 10 days after the final dose (day 38). Lumbar punctures for CSF collection were performed using standard clinical practice, aligned with Alzheimer’s Disease Neuroimaging Initiative procedures,^22^ on screening day 2 and study day 28 (after final study dose). Cerebrospinal fluid was collected in the afternoon using a gravity drip into a polypropylene collection tube. Magnetic resonance imaging was performed on screening day 1 for safety and diagnostic purposes using scanners, field strengths, and protocols employed for clinical use at each site (eTable 1 in Supplement 2). Brain FDG-PET was performed on screening day 2 and study day 28.

### Outcomes

The primary outcome of safety and tolerability was assessed via treatment-emergent adverse events (AEs), serum chemistry, hematology, urinalysis, vital signs (including orthostatic measurements), 12-lead electrocardiogram, physical examination, and suicidality assessments.^23^ The coprimary outcome of change in plasma PGRN (days 1, 7, 14, 28, and 38) and secondary outcome of change in CSF PGRN (screening day 2 and study day 28) were measured via enzyme-linked immunoassay. The cosecondary outcome of FRM-0334 plasma PK properties (maximum observed concentration [C_max_], time corresponding to C_max_ [T_max_], and area under the curve [AUC]) were assessed via liquid chromatography and tandem mass spectrometry using day 1 (before dose and 1, 2, 4, 6, 8, and 10 hours after dose) and day 7 (before dose and 1, 2, 4, 6, 8, 10, 12, and 24 hours after dose) plasma. Exploratory CSF biomarkers (NfL, Aβ_1-42_, p-tau_181_, and t-tau, measured via enzyme-linked immunoassay) and clinical measures (CDR plus NACC FTLD sum of boxes score,^19,20^ Clinical Global Impressions Scale, severity and change^24^ and Frontotemporal Dementia Rating Scale^25^) were assessed at screening and on study day 28. Biomarker assay procedures are detailed in the eMethods in Supplement 2.

### Statistical Analysis

All statistical analyses were performed in STATA, version 14.2 (StataCorp LLC). A description of the original trial protocol statistical analysis plan may be found in the eMethods in Supplement 2. Treatment-emergent AEs were compared between cohorts using the Fisher exact test with binary values (0 = patient did not have AE; 1 = patient did have AE). Given the small number of people in the placebo group, the Kruskal-Wallis test was used for pairwise comparisons of characteristics and continuous-variable outcome measurers. Given our final sample size and α of .05, we were 80% powered to detect a roughly 30% difference in change in plasma PGRN after treatment with FRM-0334 relative to placebo (*t* test, 2-tailed). Longitudinal changes in plasma PGRN, CSF biomarkers, clinical severity, and bifrontal FDG-PET standardized uptake value ratio (FDG-SUVR) were modeled with linear mixed-effects models (controlling for age and sex), allowing each patient’s outcome measures a random slope and intercept with time. In follow-up sensitivity analyses, we modeled interactions between FRM-0334 (treatment assignment [placebo vs FRM-0334], baseline AUC, and baseline C_max_) and time in determining patient outcome measures. The plasma FRM-0334 standard AUC from 0 to the maximum observed time was calculated using cubic splines.^26^ Linear regression models were used to assess for baseline relationships between baseline PK values and age, sex, FTLD clinical severity (CDR plus NACC FTLD, sum of boxes), and follow-up PK values (day 1 vs day 7). Linear regression models (controlling for age and sex) were used to investigate baseline cross-sectional linear relationships between bifrontal FDG-SUVR, plasma PGRN, CSF biomarker concentrations, and clinical severity outcome measures. Linear regression models (controlling for age and sex) were also used to investigate linear relationships between magnitude of change in PGRN (plasma or CSF) and magnitude of change in FDG-SUVR, CSF biomarker concentrations, and clinical severity measures after treatment with FRM-0334. Univariate regression models were also used to compare baseline PK values by age and sex. The Shapiro-Wilks W test was used to confirm assumptions of normality (of dependent variables and residuals), and the Cameron and Trivedi decomposition of IM-test was used to confirm assumptions of residual homoscedasticity. Non-normally distributed dependent variables were log converted before inclusion in our models. Data were analyzed from June 9, 2019, to May 13, 2021.

### FDG-PET Processing and Analysis

Baseline FDG-PET data were only available for 26 of 27 individuals randomized to the study drug. Baseline FDG-PET data for the remaining participant could not be recovered after conclusion of the trial, and this participant was excluded from our imaging analyses. A summary of scanner types and PET acquisition parameters is detailed in eTable 1 in Supplement 2. Digital Imaging and Communications in Medicine files from a total of 26 trial patients and 52 age-matched individuals without cognitive pathology from the Berkeley Aging Cohort Study were converted to Neuroimaging Informatics Technology Initiative format, warped to standard space with Statistical Parametric Mapping 12 (SPM12) via a PET-only pipeline (eMethods in Supplement 2) and normalized to pons to obtain parametric FDG-SUVR images. Given the heterogeneity of scanner and acquisition parameters (eTable 1 in Supplement 2), final FDG-PET SUVR images were downsampled to match the scan with the least spatial resolution using Analysis of Functional NeuroImages software, version 16.2.16 (NIMH Scientific and Statistical Computing Core). Voxelwise group comparisons (gene variation carriers vs controls) of FDG-SUVR were assessed via analysis of covariance in SPM12, controlling for age and sex, in separate models including prodromal, symptomatic, and all gene variation carriers. Voxelwise associations between FDG-SUVR and 2 primary exploratory measures of FTD severity (CSF NfL and CDR plus NACC FTLD sum of boxes) were assessed in separate multiple regression models (with age and sex entered as covariates) in SPM12, including all variation carriers and excluding controls. T-maps from voxelwise analyses were thresholded (primary threshold *P* < .001 uncorrected for multiple comparisons, cluster-level familywise error [FWE]–corrected *P* < .05), converted to Pearson correlation coefficient *r* maps using the Computational Anatomy Toolbox, version 12 (Jena University Hospital, Departments of Psychiatry and Neurology), and rendered on a 3-dimensional brain surface using BrainNet Viewer.^27^ A more stringent primary threshold of *P* < .05 FWE-corrected for multiple comparisons was also used for supplemental sensitivity analyses. Based on our initial voxelwise results (revealing a chiefly bifrontal pattern of hypometabolism in *GRN* variation carriers) we further investigated potential linear relationships between FDG metabolism in a bifrontal region of interest (ROI) and clinical measures or fluid biomarkers. Baseline bifrontal macro ROI FDG-SUVR values were calculated from weighted averages of constituent bilateral frontal SPM12 using ROI definitions from the Neuromorphometrics Atlas^28^ and compared (as the dependent variable) with clinical measures and fluid biomarkers using linear regression models controlling for age and sex. The included ROIs were anterior, lateral, medial, and posterior orbital gyri, middle and superior frontal gyri, orbital and triangular part of the inferior frontal gyri, and the frontal pole. Change in bifrontal FDG-SUVR over time was also assessed via linear mixed-effects models as a follow-up sensitivity analysis, though the heterogeneity of PET sites and short duration of follow-up limited the potential interpretability of these analyses. The methods of FDG-PET collection and processing (including estimation of W-maps) are further detailed in the eMethods in Supplement 2.

### Magnetic Resonance Imaging Processing

Magnetic resonance imaging processing methods and scanner characteristics are further detailed in the eMethods and eTable 1 in Supplement 2. Though the heterogeneity of scanners and field strengths across sites limited the interpretability of volumetric data, each participant’s relative degree of total brain atrophy was estimated by dividing total parenchymal volume by total intracranial volume. This parenchymal volume variable was exclusively used as a covariate in follow-up sensitivity analyses, controlling for the potential effect of atrophy in multivariate regression models analyzing bifrontal ROI FDG-SUVR.

## Results

A total of 27 participants (mean [SD] age, 56.6 [10.5] years; 16 women [59.3%] and 11 men [40.7%]; 1 Black participant [3.7%] and 26 White participants [96.3%]) with *GRN* variations were randomized and completed treatment (eFigure in Supplement 2). Demographic characteristics of individuals randomized to drug or placebo did not differ at baseline (Table 1). Compared with prodromal *GRN* variation carriers, participants with symptomatic FTD had more severe scores on the CDR plus NACC FTLD sum of boxes, Clinical Global Impressions Scale-severity, and Frontotemporal Dementia Rating Scale, as well as elevated CSF NfL and CSF t-tau level, but did not otherwise differ demographically. No discontinuations or dose-limiting adverse effects occurred throughout treatment. Incidence of treatment-emergent AEs (Table 2) was similar between placebo (4 of 5; 80%), low-dose (7 of 11; 63.6%), and high-dose (7 of 11; 63.6%) cohorts (*P* > .99 for all comparisons). One patient randomized to 500 mg FRM-0334 experienced a serious AE (deep vein thrombosis with nonfatal pulmonary embolism), which was not felt to be related to the study drug in the opinion of the site investigator. Patients randomized to FRM-0334 experienced higher incidences of cardiac, constitutional, dermatologic, infectious, psychiatric, and respiratory symptoms and a lower incidence of gastrointestinal symptoms relative to placebo-treated participants (Table 2).

**Table 1.** Participant Characteristics^a^

Characteristic	Mean (SD)^b^				
	*GRN* variation carriers by treatment assignment			*GRN* variation carriers	
	Placebo (n = 5)	FRM-0334		Prodromal (n = 8)	Symptomatic (n = 19)
		300 mg (n = 11)	500 mg (n = 11)		
Placebo, No. (%)	NA	NA	NA	1 (12.5)	4 (21.1)
FRM-0334 300 mg, No. (%)	NA	NA	NA	2 (25.0)	9 (47.4)
FRM-0334 500 mg, No. (%)	NA	NA	NA	5 (62.5)	6 (31.6)
Sex, No. (%)					
Women	3 (60.0)	7 (63.6)	6 (54.5)	4 (50.0)	12 (63.2)
Men	2 (40.0)	4 (36.4)	5 (45.5)	4 (50.0)	7 (36.8)
Age, y	55.6 (5.9)	59 (9.7)	54.2 (11.1)	51.6 (10.5)	58.4 (8.8)
Baseline clinical severity					
Prodromal/symptomatic	1/4	2/9	5/6	NA	NA
CDR plus NACC FTLD, sum of boxes	4.2 (6.8)	8 (6.1)	4.1 (7)	0	9.9 (5.4)^c^
CGI-S	2.7 (1.5)	3.6 (1.6)	2 (1.7)	1 (0.0)	4.2 (0.9)^c^
FRS	80 (32)	42 (32)	23 (9)	100 (0)	39 (28)^c^
Baseline biomarker data					
Plasma PGRN, pg/mL	8490 (2120)	9190 (2290)	11020 (2990)	9780 (2800)	9680 (2670)
CSF, pg/mL					
PGRN	375 (78)	359 (110)	393 (128)	399 (138)	363 (99)
NfL	1170 (1610)	3070 (2400)	2000 (2340)	627 (435)	3080 (2400)^c^
Aβ_1-42_	1020 (232)	792 (222)	920 (240)	912 (197)	867 (256)
p-Tau_181_	39.4 (2.5)	66.2 (66.8)	41.5 (11.3)	40.8 (12.7)	57.5 (54.3)
Total tau	337 (101)	306 (197)	277 (158)	185 (63.2)	349 (172)^c^
Bilateral frontal FDG-SUVR	1.4 (0.2)	1.2 (0.3)	1.4 (0.3)	1.5 (0.2)	1.2 (0.3)^c^
Plasma FRM-0334 pharmacokinetics					
Visit 1					
AUC, h × ng/mL	NA	4760 (1470)	4080 (1880)	NA	NA
VC_max,_ ng/mL	NA	1110 (339)	887 (404)	NA	NA
VT_max_, h	NA	2.7 (2)	2.6 (1.1)	NA	NA
Visit 7					
AUC, h × ng/mL	NA	5650 (2000)	6180 (3250)	NA	NA
C_max_, ng/mL	NA	899 (373)	936 (380)	NA	NA
T_max_, h	NA	2.9 (1.0)	3.1 (1.2)	NA	NA

Abbreviations: Aβ_1-42_, amyloid β_1-42_; AUC, area under the curve (plasma exposure); CDR plus NACC FTLD, CDR Dementia Staging Instrument plus National Alzheimer’s Coordinating Center frontotemporal lobar degeneration behavior and language domain; CGI-S, Clinical Global Impressions Scale Baseline Severity; C_max_, maximum plasma concentration; CSF, cerebrospinal fluid; FRS, Frontotemporal Dementia Rating Scale; NfL, neurofilament light chain; PGRN, progranulin; p-tau_181_, phosphorylated tau 181; T_max_, time to maximum plasma concentration.

^a^ Details on race and ethnicity are not presented in order to protect patient confidentiality.

^c^ Baseline Kruskal-Wallis test *P* < .05 in comparison between asymptomatic and symptomatic patients, whereas no other pairwise comparisons (placebo vs FRM-0334, high-dose vs low-dose FRM-0334, prodromal vs asymptomatic) met *P* < .05.

**Table 2.** Adverse Events by Treatment Assignment

Variable	No. (%)			
	Placebo (n = 5)	FRM-0334		
		300 mg (n = 11)	500 mg (n = 11)	All (n = 22)
Total experiencing AEs	4 (80.0)	7 (63.6)	7 (63.6)	14 (63.6)
Cardiovascular symptoms	0	1 (9.1)	3 (27.3)	4 (18.2)
Bradycardia	0	0	1 (9.1)	1 (4.5)
Tachycardia	0	0	1 (9.1)	1 (4.5)
Orthostatic hypotension	0	1 (9.1)	0	1 (4.5)
Abdominal hematoma	0	0	1 (9.1)	1 (4.5)
PE/DVT (SAE)	0	0	1 (9.1)	1 (4.5)
Constitutional symptoms	0	0	2 (18.2)	2 (9.1)
Fatigue	0	0	1 (9.1)	1 (4.5)
Decreased appetite	0	0	1 (9.1)	1 (4.5)
Dermatologic symptoms	0	0	2 (18.2)	2 (9.1)
Erythroderma	0	0	1 (9.1)	1 (4.5)
Spider nevus	0	0	1 (9.1)	1 (4.5)
Gastrointestinal symptoms	3 (60.0)	3 (27.3)	4 (36.4)	7 (31.8)
Abdominal pain and dyspepsia	2 (40.0)	0	1 (9.1)	1 (4.5)
Diarrhea	1 (20.0)	3 (27.3)	2 (18.2)	5 (22.7)
Nausea and vomiting	1 (20.0)	0	4 (36.4)	4 (18.2)
Bloating	0	1 (9.1)	0	1 (4.5)
Dental pain	1 (20.0)	0	0	0
Infections	0	3 (27.3)	3 (27.3)	6 (27.3)
Cold sores	0	0	1 (9.1)	1 (4.5)
Viral				
Upper respiratory infection	0	2 (18.2)	2 (18.2)	4 (18.2)
Gastritis	0	1 (9.1)	0	1 (4.5)
Musculoskeletal symptoms	0	1 (9.1)	1 (9.1)	2 (9.1)
Neck pain	0	0	1 (9.1)	1 (4.5)
Weakness	0	1 (9.1)	0	1 (4.5)
Neurologic symptoms	1 (20.0)	3 (27.3)	4 (36.4)	7 (31.8)
Headache	1 (20.0)	2 (18.2)	4 (36.4)	6 (27.3)
Worsening FTD symptoms	0	1 (9.1)	0	1 (4.5)
Psychiatric symptoms	0	1 (9.1)	3 (27.3)	4 (18.2)
Worsening apathy	0	0	1 (9.1)	1 (4.5)
Insomnia	0	0	1 (9.1)	1 (4.5)
Worsening				
Depression	0	0	1 (9.1)	1 (4.5)
Bruxism	0	1 (9.1)	0	1 (4.5)
Respiratory symptoms	0	0	2 (18.2)	2 (9.1)
Cough	0	0	1 (9.1)	1 (4.5)
Epistaxis	0	0	1 (9.1)	1 (4.5)
Laboratory abnormalities	1 (20.0)	1 (9.1)	3 (27.3)	4 (18.2)
Low white blood cell count	1 (20.0)	0	0	0
High urine bilirubin	0	0	1 (9.1)	1 (4.5)
Elevated				
Liver function tests	0	1 (9.1)	0	1 (4.5)
Cholesterol	0	0	1 (9.1)	1 (4.5)
Hematuria	0	0	1 (9.1)	1 (4.5)

Abbreviations: AE, adverse event; DVT, deep vein thrombosis; FTD, frontotemporal degeneration; PE, pulmonary embolism; SAE, severe adverse event.

**Figure 1.**
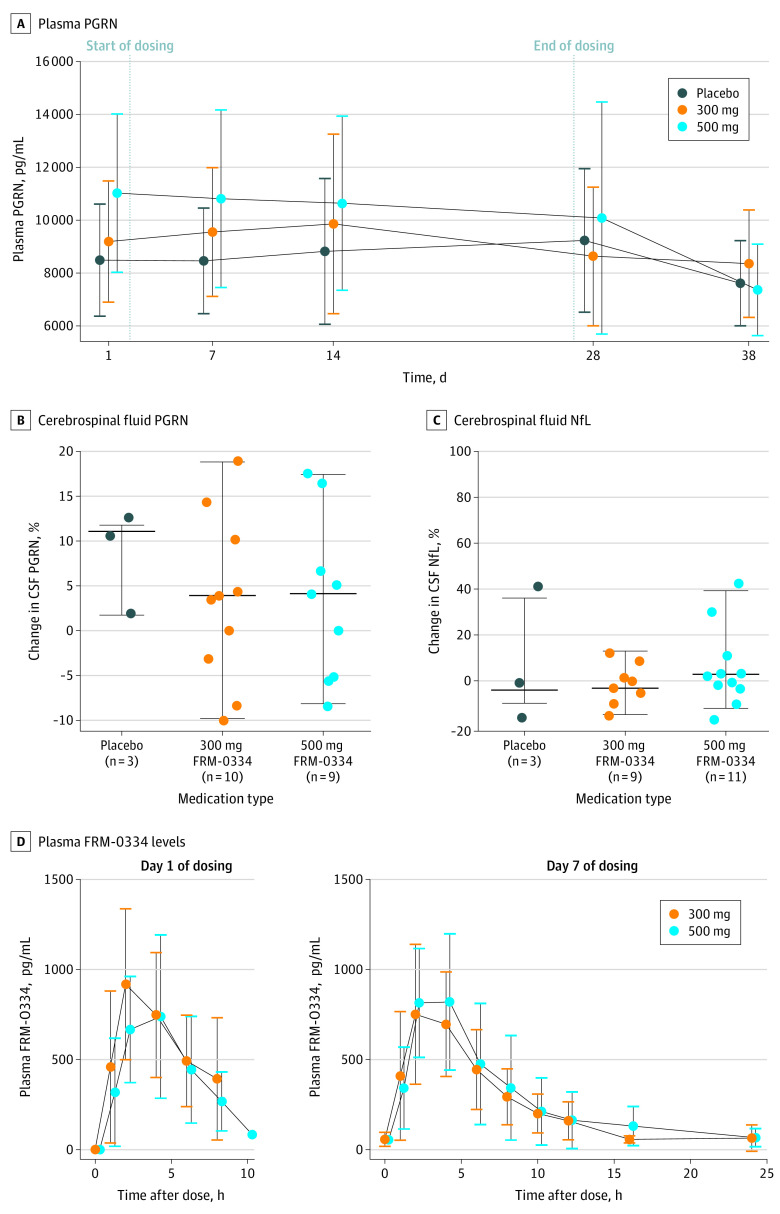
Pharmacodynamic and Pharmacokinetic Properties of FRM-0334 in Participants With *GRN* Haploinsufficiency After 28 days of treatment, patients randomized to FRM-0334 did not experience an improvement in plasma progranulin (PGRN) relative to baseline or patients randomized to placebo (A). After 28 days of treatment, patients randomized to FRM-0334 did not experience a consistent improvement in cerebrospinal fluid PGRN (B) or CSF neurofilament light chain (NfL) (C). Plasma FRM-0334 pharmacokinetic measures (including area under the curve [AUC] and the maximum observed concentration [C_max_]) did not increase in proportion to dose on day 1 and day 7 of dosing (D).

There was no effect of FRM-0334 on concentrations of plasma PGRN (4.3 pg/mL per day change after treatment; 95% CI, −10.1 to 18.8; *P* = .56), CSF PGRN (0.42 pg/mL per day; 95% CI, −0.12 to 0.95; *P* = .13) (Figure 1A and B), or exploratory PD outcomes (CSF NfL, CSF Aβ_1-42_, CSF p-tau_181_, and CSF t-tau, CDR plus NACC FTLD sum of boxes, Frontotemporal Dementia Rating Scale, and bifrontal FDG-SUVR) after 28 days of treatment (Figure 1C; eTable 2 in Supplement 2). Additionally, treatment assignment, baseline FRM-0334 plasma AUC, and baseline FRM-0334 plasma C_max_ were not associated with improvement in PD outcomes (eTable 2 in Supplement 2).

Plasma FRM-0334 exposure did not rise in proportion to dose (Table 1, Figure 1D). Compared with the low-dose cohort, the high-dose cohort did not consistently experience higher AUC (high dose, 4080 hours × ng/mL [1880] vs low dose, 4760 hours × ng/mL [1470]; *P* = .18) or C_max_ (high dose, 887 ng/mL [404] vs low dose, 1110 ng/mL [339]; *P* = .11) values on day 1. Compared with the low- dose cohort, the high-dose cohort also did not consistently experience higher AUC (high dose, 6180 hours × ng/mL [3250] vs low dose, 5650 hours × ng/mL [2000]; *P* = .62) or C_max_ (high dose, 936 ng/mL [380] vs low dose, 889 ng/mL [373]; *P* = .67) values on day 7. Older age (but not clinical severity, plasma/CSF PGRN, or sex) predicted higher baseline AUC (84-hour × ng/mL per year of age, 95% CI, 20.2-148; *P* = .01, *R^2^* = 0.27) and C_max_ (23.3 mg/mL per year of age, 95% CI, 10.2-36.5; *P* = .001, *R^2^* = 0.41) (eTable 3 in Supplement 2). Day 1 and day 7 AUC were linearly related (b = 0.52; 95% CI, 0.35-0.69; *P* < .001; *R^2^* = 0.64), although day 1 and day 7 C_max_ was not (b = 0.39; 95% CI, –0.06 to 0.84; *P* = .08; *R^2^* = 0.14). In patients who received FRM-0334, change in PGRN (plasma and CSF) was not linearly related to change in bifrontal FDG-SUVR, CSF NfL, CSF Aβ_1-42_, CSF total tau, or measures of clinical severity, although an increase in CSF PGRN was associated with a decrease in p-tau_181_ (b = −2.3; 95% CI, −4.2 to −0.4; *P* = .02) (eTable 3 in Supplement 2).

**Figure 2.**
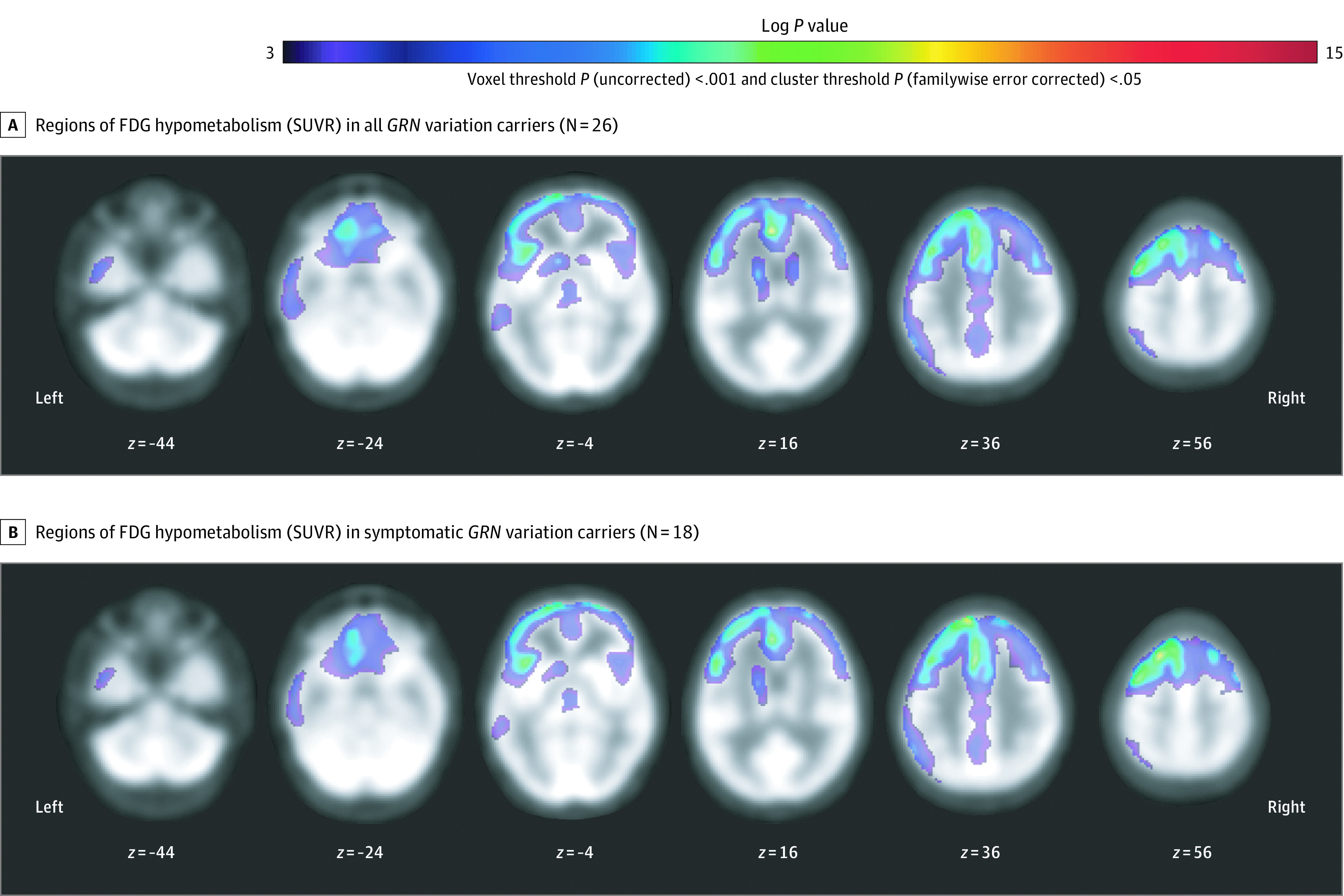
Hypometabolism in *GRN* Variation Carriers Relative to Age-Matched Controls Regions of fluorodeoxyglucose (FDG) hypometabolism in all *GRN* variation carriers (A) and in symptomatic *GRN* variation carriers (B). The depicted results meet a primary voxel-level threshold of uncorrected *P* < .001 and a cluster-level threshold of familywise error *P* < .05. Voxels meeting a more stringent threshold of familywise error *P* < .05 are detailed in eTable 3 in Supplement 2. No voxels met this threshold in prodromal variation carriers (n = 8). SUVR indicates standardized uptake value ratio.

**Figure 3.**
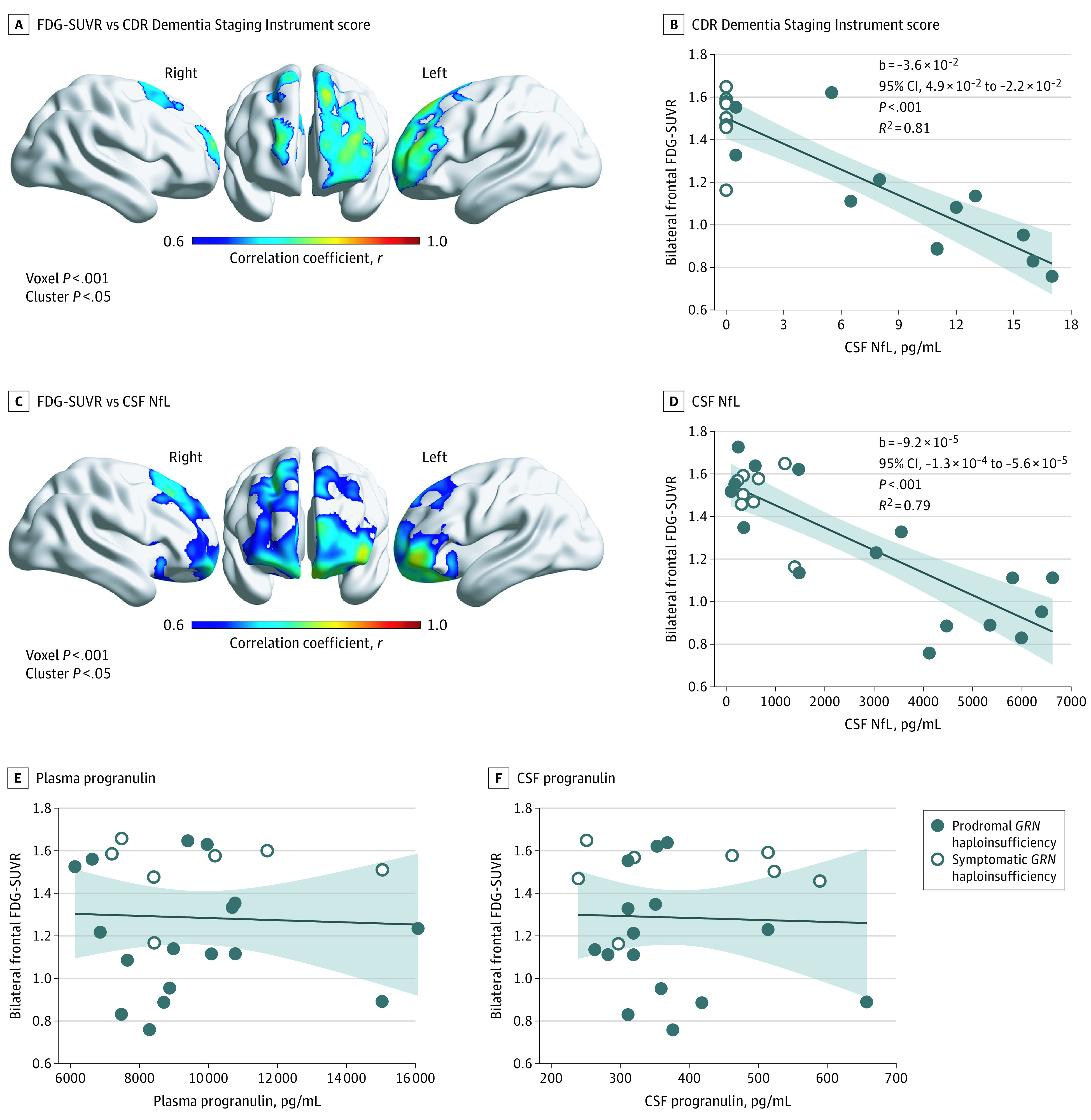
Frontal Fluorodeoxyglucose (FDG) Hypometabolism vs Clinical Severity and Cerebrospinal Fluid (CSF) Biomarkers in *GRN* Gene Variation Carriers In voxelwise analyses, low frontal FDG–standardized uptake value ratio (SUVR) was associated with higher Clinical Dementia Rating (CDR) plus National Alzheimer’s Coordinating Center (NACC) frontotemporal lobar degeneration (FTLD) sum of boxes score (A) and higher CSF neurofilament light chain (NfL) (C). Low bifrontal FDG-SUVR on a bifrontal composite region of interest was also associated with high CDR plus NACC FTLD sum of boxes score (B), and CSF NfL (D) but not plasma progranulin (E) or CSF progranulin (F). The shaded areas in panels B, D, E, and F represent the 95% CIs.

*GRN* variation carrier status, particularly symptomatic disease (but not prodromal carrier status alone), was associated with frontal FDG hypometabolism relative to controls (including left > right dorsal prefrontal, anterior cingulate, orbitofrontal, inferior frontal gyrus, and insular hypometabolism), with additional left-predominant hypometabolism noted in lateral parietal, lateral temporal, posterior cingulate, caudate, and thalamic regions (Figure 2; eTable 4 in Supplement 2). In voxelwise regression analyses including only gene variation carriers with available PET data (n = 26), greater clinical severity (CDR plus NACC FTLD sum of boxes) was associated with left greater than right dorsal and lateral prefrontal FDG hypometabolism (Figure 3; eTable 4 in Supplement 2). High CSF NfL was also associated with dorsal prefrontal and left > right orbitofrontal FDG hypometabolism (Figure 3; eTable 4 in Supplement 2). In a bifrontal ROI, low FDG-SUVR was associated with greater CDR plus NACC FTLD sum of boxes score (b = −3.6 × 10^−2^ SUVR units/CDR units; 95% CI, –4.9 × 10^−2^ to −2.2 × 10^−2^; *P* < .001), greater Clinical Global Impressions Scale severity (b = −1 × 10^−1^ SUVR units/CGI units; 95% CI, –1 × 10^−1^ to −6 × 10^−2^; *P* < .0005), lower Frontotemporal Dementia Rating Scale score (b = −8.8 × 10^−3^ SUVR units/Frontotemporal Dementia Rating Scale units; 95% CI, –3.7 × 10^−3^ to 1.3 × 10^−2^; *P* < .001), elevated CSF NfL (b = −9.2 × 10^−5^ SUVR units/pg NfL/mL; 95% CI, –1.3 × 10^−4^ to −5.6 × 10^−5^; *P* < .001), and high CSF t-tau (−7.2 × 10^−4^ SUVR units/pg t-tau/mL; 95% CI, −1.4 × 10^−3^ to −9.5 × 10^−5^; *P* = .03), but was not linearly related to plasma PGRN, CSF PGRN, CSF Aβ_1-42_, or CSF p-tau_181_ (Figure 3; eTable 3 in Supplement 2). In follow-up sensitivity analyses, including parenchymal volume as a covariate, frontal hypometabolism was still linearly related to clinical severity (b = −1.8 × 10^−2^ sum of boxes units/SUVR units, 95% CI, −3.5 × 10^−2^ to −4.2 × 10^−4^; *R^2^* = 0.88; *P* < .045). Greater clinical severity (CDR plus NACC FTLD sum of boxes) was linearly related to high CSF NfL, but not plasma PGRN, CSF PGRN, CSF Aβ_1-42_, CSF t-tau, or CSF p-tau_181_ (eTable 3 in Supplement 2).

## Discussion

This report describes, to our knowledge, the first international multicenter randomized, placebo-controlled clinical trial in individuals with *GRN* haploinsufficiency and the first clinical exploration of HDAC inhibition in this cohort. Treatment with FRM-0334 was safe and well tolerated but was not associated with improvement in plasma PGRN concentration, CSF PGRN concentration, or exploratory pharmacodynamic measures. Moreover, the plasma PK profile of FRM-0334 did not change in proportion to the oral dose, which suggests inconsistent absorption or oral bioavailability of the formulation used in this study. Although these results halted clinical development of the current formulation of FRM-0334 in *GRN* haploinsufficiency, this trial’s international multicenter cohort enabled novel opportunities for additional biomarker analyses. Specifically, we found that bifrontal cortical FDG hypometabolism was associated with greater clinical disease severity and CSF measures of neurodegeneration including NfL and t-tau levels in patients with PGRN deficiency.

The PD and PK profiles of oral FRM-0334 firmly suggest that further development of the tested formulation should not be pursued for *GRN* haploinsufficiency. Histone acetylation is primarily driven by FRM-0334 C_max_, and within this trial, only a minority of participants in both the low- and high-dose cohorts experienced a C_max_ above 1000 ng/mL (a magnitude equivalent to the minimum C_max_ necessary to drive detectable histone acetylation in preclinical canine studies). Moreover, the observed variance in FRM-0334 C_max_ did not predict change in PGRN (plasma and CSF) or other exploratory PD measures, further suggesting inadequate target engagement. Additionally, given the unreliable relationship between FRM-0334 dose level, plasma C_max_, and plasma AUC, it is unknown whether alternative regimens (including higher or more frequent doses) of the current formulation could reliably improve the bioavailability of FRM-0334 in future trials. Given the relationship between FRM-0334 exposure and age, future early phase trials of other orally available HDAC inhibitors may require design considerations that account for possible unique, age-related differences in drug oral bioavailability in GRN haploinsufficiency. Given the wide distribution of *GRN* expression,^29^ particularly in the liver, it is possible that *GRN* haploinsufficiency could give rise to a wide array of subtle phenotypic differences in a variety of cell types, including peripheral cells mediating drug absorption or metabolism.

Our FDG-PET analyses were consistent with previous clinical correlations^30^ and anatomic descriptions of frontal hypometabolism^30,31^ in *GRN* haploinsufficiency but reveal novel fluid-biomarker relationships to FDG-PET data. Cerebrospinal fluid NfL is interpreted to be a biomarker of axonal injury and neuronal degeneration^32^ and correlates with disease severity^33,34^ and aggressiveness in sporadic FTLD as well as in *GRN* haploinsufficiency.^35^ The observed relationship with NfL level may attest to the synaptic loss and neuronal metabolism documented on FDG-PET (via astrocyte-neuron metabolic coupling^36^). Additionally, although *GRN* haploinsufficiency is not typified by tau deposition on autopsy, our findings support previous observations of elevated CSF t-tau in symptomatic disease.^37^ The relationship of FDG-PET to t-tau may reflect the lysosomal role of PGRN in supporting autophagy pathways,^38^ which may impact tau clearance.^39^ Fluorodeoxyglucose hypometabolism was not associated with CSF p-tau_181_, therefore, comorbid Alzheimer pathology is unlikely to have mediated its relationship to t-tau. It is unclear how much of our FDG-PET fluid-biomarker correlations were mediated by brain atrophy alone, given our lack of complementary harmonized magnetic resonance images. Our sensitivity analyses, accounting for whole-brain parenchymal volume, did provide some evidence that FDG hypometabolism tracks clinical GRN deficiency severity even after the effect of volume is considered.

### Limitations

Our study had several limitations affecting its interpretability. The inconsistent bioavailability of FRM-0334 made it difficult to reliably extrapolate the broader effect of consistent HDAC inhibition in PGRN deficiency or whether higher exposure levels of FRM-0334 may have affected plasma PGRN. The small sample size of patients randomized to placebo and the relatively short duration of this trial also limited our ability to detect modest deflections in biomarker trajectory. However, recent publicly presented open-label trial data do suggest that a similar duration and smaller sample size may be sufficient to demonstrate biomarker evidence of target engagement from alternative PGRN boosting strategies.^40,41^ With regard to our FDG-PET analyses, the heterogeneity of our data (acquired on differing scanners, with differing resolutions and protocols, in the absence of site-specific, healthy controls) may have also limited the quality and resolution of our observations. Moreover, the short duration of our trial provided a relatively short interval to validly discern a true trajectory of change within our heterogeneous data set. Despite these limitations, our novel observations of FDG hypometabolism support further exploration of FDG-PET as an imaging biomarker of FTLD biological severity in future *GRN* haploinsufficiency studies (ideally employing greater homogeneity and harmonization among scanners, acquisition protocols, and contemporaneous magnetic resonance imaging acquisition).

## Conclusions

In conclusion, this randomized clinical trial demonstrated that FRM-0334 did not have a PD effect on CSF or plasma PGRN concentration. However, this study provided useful data to inform future clinical trial methodology in similar cohorts. Given the rarity of patients with *GRN* haploinsufficiency, this trial demonstrates that international multicenter studies are feasible in this indication.^1,2,3,4,5,6^ Such multicenter trials provide a unique opportunity to collect valuable observational data in rare familial forms of FTD.

## Notes

Supplement 1.
Trial Protocol

Supplement 2. **eFigure.** Consort Diagram **eTable 1.** Clinical Sites and Corresponding PET and MRI Scanner Characteristics **eTable 2.** Predicted Change Over Time in Primary, Secondary, and Exploratory Measures **eTable 3.** Baseline Linear Regression Analyses Comparing Among Patient Characteristics and Outcome Measures **eTable 4.** Supplemental FDG-SUVR Sensitivity Analyses Using a More Stringent Voxel Threshold of P_FWE_ < .05
eMethods

eReferences

Supplement 3.
Data Sharing Statement

## References

[1] Sha SJ, Miller ZA, Min SW, . An 8-week, open-label, dose-finding study of nimodipine for the treatment of progranulin insufficiency from *GRN* gene mutations. Alzheimers Dement (N Y). 2017;3(4):507-512. doi:10.1016/j.trci.2017.08.002 29124108 PMC5671622

[2] Alector. Our science. Accessed June 20, 2020. https://alector.com/our-science/

[3] Prevail. Our pipeline. Accessed February 17, 2020. https://www.prevailtherapeutics.com/programs/

[4] Arkuda Therapeutics. Discovery. Accessed June 20, 2020. https://www.arkudatx.com/discovery/

[5] Passage Bio. Pipeline. Accessed June 20, 2020. https://www.passagebio.com/our-science/pipeline/

[6] Denali. Our pipeline. Accessed June 20, 2020. https://denalitherapeutics.com/pipeline

[7] Baker M, Mackenzie IR, Pickering-Brown SM, . Mutations in progranulin cause tau-negative frontotemporal dementia linked to chromosome 17. Nature. 2006;442(7105):916-919. doi:10.1038/nature05016 16862116

[8] Gass J, Cannon A, Mackenzie IR, . Mutations in progranulin are a major cause of ubiquitin-positive frontotemporal lobar degeneration. Hum Mol Genet. 2006;15(20):2988-3001. doi:10.1093/hmg/ddl241 16950801

[9] Shankaran SS, Capell A, Hruscha AT, . Missense mutations in the progranulin gene linked to frontotemporal lobar degeneration with ubiquitin-immunoreactive inclusions reduce progranulin production and secretion. J Biol Chem. 2008;283(3):1744-1753. doi:10.1074/jbc.M705115200 17984093

[10] Kao AW, McKay A, Singh PP, Brunet A, Huang EJ. Progranulin, lysosomal regulation and neurodegenerative disease. Nat Rev Neurosci. 2017;18(6):325-333. doi:10.1038/nrn.2017.36 28435163 PMC6040832

[11] Meeter LHH, Patzke H, Loewen G, . Progranulin levels in plasma and cerebrospinal fluid in granulin mutation carriers. Dement Geriatr Cogn Dis Extra. 2016;6(2):330-340. doi:10.1159/000447738 27703466 PMC5040889

[12] Finch N, Baker M, Crook R, . Plasma progranulin levels predict progranulin mutation status in frontotemporal dementia patients and asymptomatic family members. Brain. 2009;132(pt 3):583-591. doi:10.1093/brain/awn352 19158106 PMC2664450

[13] A phase 3 study to evaluate efficacy and safety of AL001 in frontotemporal dementia (INFRONT-3). Clinicaltrials.gov identifier: NCT04374136. Accessed August 21, 2020. https://clinicaltrials.gov/ct2/show/NCT04374136?term=NCT04374136&draw=2&rank=1

[14] Patzke H, Albayya F, Besterman J. Development of the novel histone deacetylase inhibitor EVP-0334 for CNS indications. Poster presented at: 38th Annual Meeting of the Society for Neuroscience; November 19, 2008; Washington, DC.

[15] Cenik B, Sephton CF, Dewey CM, . Suberoylanilide hydroxamic acid (vorinostat) up-regulates progranulin transcription: rational therapeutic approach to frontotemporal dementia. J Biol Chem. 2011;286(18):16101-16108. doi:10.1074/jbc.M110.193433 21454553 PMC3091219

[16] Alquezar C, Esteras N, de la Encarnación A, Moreno F, López de Munain A, Martín-Requero Á. Increasing progranulin levels and blockade of the ERK1/2 pathway: upstream and downstream strategies for the treatment of progranulin deficient frontotemporal dementia. Eur Neuropsychopharmacol. 2015;25(3):386-403. doi:10.1016/j.euroneuro.2014.12.007 25624003

[17] Bubna AK. Vorinostat-an overview. Indian J Dermatol. 2015;60(4):419. doi:10.4103/0019-5154.160511 26288427 PMC4533557

[18] Hanson JE, La H, Plise E, . SAHA enhances synaptic function and plasticity in vitro but has limited brain availability in vivo and does not impact cognition. *PLoS One**.* 2013;8(7):e69964. doi:10.1371/journal.pone.0069964 23922875 PMC3724849

[19] Knopman DS, Kramer JH, Boeve BF, . Development of methodology for conducting clinical trials in frontotemporal lobar degeneration. Brain. 2008;131(pt 11):2957-2968. doi:10.1093/brain/awn234 18829698 PMC2725027

[20] Miyagawa T, Brushaber D, Syrjanen J, ; ARTFL/LEFFTDS Consortium. Use of the CDR® plus NACC FTLD in mild FTLD: data from the ARTFL/LEFFTDS consortium. Alzheimers Dement. 2020;16(1):79-90. doi:10.1016/j.jalz.2019.05.013 31477517 PMC6949373

[21] Ruikar V. Interactive voice/web response system in clinical research. Perspect Clin Res. 2016;7(1):15-20. doi:10.4103/2229-3485.173781 26952178 PMC4763512

[22] Alzheimer’s Disease Neuroimaging Initiative. Study documents. Accessed May 13, 2021. http://adni.loni.usc.edu/methods/documents/

[23] Posner K, Brown GK, Stanley B, . The Columbia-Suicide Severity Rating Scale: initial validity and internal consistency findings from three multisite studies with adolescents and adults. Am J Psychiatry. 2011;168(12):1266-1277. doi:10.1176/appi.ajp.2011.10111704 22193671 PMC3893686

[24] Busner J, Targum SD. The clinical global impressions scale: applying a research tool in clinical practice. Psychiatry (Edgmont). 2007;4(7):28-37. 20526405 PMC2880930

[25] Mioshi E, Hsieh S, Savage S, Hornberger M, Hodges JR. Clinical staging and disease progression in frontotemporal dementia. Neurology. 2010;74(20):1591-1597. doi:10.1212/WNL.0b013e3181e04070 20479357

[26] Stata.com. Pharmacokinetic (biopharmaceutical) data. Accessed June 13, 2020. https://www.stata.com/manuals13/rpk.pdf

[27] Xia M, Wang J, He Y. BrainNet Viewer: a network visualization tool for human brain connectomics. *PLoS One**.* 2013;8(7):e68910. doi:10.1371/journal.pone.0068910 23861951 PMC3701683

[28] Neuromorphometrics, Inc. Modeling the living human brain. Accessed May 13, 2021. http://www.neuromorphometrics.com

[29] Bhandari V, Giaid A, Bateman A. The complementary deoxyribonucleic acid sequence, tissue distribution, and cellular localization of the rat granulin precursor. Endocrinology. 1993;133(6):2682-2689. doi:10.1210/endo.133.6.8243292 8243292

[30] Jacova C, Hsiung GYR, Tawankanjanachot I, . Anterior brain glucose hypometabolism predates dementia in progranulin mutation carriers. Neurology. 2013;81(15):1322-1331. doi:10.1212/WNL.0b013e3182a8237e 24005336 PMC3806924

[31] Spina S, Murrell JR, Huey ED, . Clinicopathologic features of frontotemporal dementia with progranulin sequence variation. Neurology. 2007;68(11):820-827. doi:10.1212/01.wnl.0000254460.31273.2d 17202431

[32] Petzold A. Neurofilament phosphoforms: surrogate markers for axonal injury, degeneration and loss. J Neurol Sci. 2005;233(1-2):183-198. doi:10.1016/j.jns.2005.03.015 15896809

[33] Scherling CS, Hall T, Berisha F, . Cerebrospinal fluid neurofilament concentration reflects disease severity in frontotemporal degeneration. Ann Neurol. 2014;75(1):116-126. doi:10.1002/ana.24052 24242746 PMC4020786

[34] Ljubenkov PA, Staffaroni AM, Rojas JC, . Cerebrospinal fluid biomarkers predict frontotemporal dementia trajectory. Ann Clin Transl Neurol. 2018;5(10):1250-1263. doi:10.1002/acn3.643 30349860 PMC6186942

[35] Meeter LH, Dopper EG, Jiskoot LC, . Neurofilament light chain: a biomarker for genetic frontotemporal dementia. Ann Clin Transl Neurol. 2016;3(8):623-636. doi:10.1002/acn3.325 27606344 PMC4999594

[36] Bélanger M, Allaman I, Magistretti PJ. Brain energy metabolism: focus on astrocyte-neuron metabolic cooperation. Cell Metab. 2011;14(6):724-738. doi:10.1016/j.cmet.2011.08.016 22152301

[37] Carecchio M, Fenoglio C, Cortini F, . Cerebrospinal fluid biomarkers in Progranulin mutations carriers. J Alzheimers Dis. 2011;27(4):781-790. doi:10.3233/JAD-2011-111046 21891865

[38] Chang MC, Srinivasan K, Friedman BA, . Progranulin deficiency causes impairment of autophagy and TDP-43 accumulation. J Exp Med. 2017;214(9):2611-2628. doi:10.1084/jem.20160999 28778989 PMC5584112

[39] Lee MJ, Lee JH, Rubinsztein DC. Tau degradation: the ubiquitin-proteasome system versus the autophagy-lysosome system. Prog Neurobiol. 2013;105:49-59. doi:10.1016/j.pneurobio.2013.03.001 23528736

[40] Paul R. A phase 1 study of AL001 in healthy volunteers and frontotemporal dementia patients carrying a granulin mutation. Presented at: Alzheimer’s Association International Conference; July 17, 2019; Los Angeles, CA.

[41] Paul R. AL001 phase 1b/2 update. Presented at: Alzheimer’s Association International Conference; July 28, 2020; Amsterdam, the Netherlands.

