# Supplementary material for: Effect of the Histone Deacetylase Inhibitor FRM-0334 on Progranulin Levels in Patients With Progranulin Gene Haploinsufficiency: A Randomized Clinical Trial
Source: JAMA Netw Open. 2021 Sep 24;4(9):e2125584. doi: 10.1001/jamanetworkopen.2021.25584 (PMC8463943; doi:10.1001/jamanetworkopen.2021.25584)
Supplement: Supplement 1. — Trial Protocol [file jamanetwopen-e2125584-s001.pdf]

CLINICAL STUDY PROTOCOL

A Randomized, Double-Blind, Placebo-Controlled, Dose-Escalating, Phase 2a Safety, Tolerability, and Pharmacodynamic Study of Two Doses of an Histone Deacetylase Inhibitor (FRM-0334) in Subjects with Prodromal to Moderate Frontotemporal Dementia with Granulin Mutation

Protocol Number: FRM-0334-002

Study Phase: 2a

EudraCT Number: 2014-001489-85

SPONSOR:

FORUM Pharmaceuticals Inc.
500 Arsenal Street
Watertown, MA 02472 United States

CONTRACT RESEARCH ORGANIZATION:

Worldwide Clinical Trials
401 N. Maple Drive
Beverly Hills, CA 90210 United States

Issue Date: 01 August 2014 (Amendment 1)

Supersedes Protocol Issued: 13 May 2014

CONFIDENTIALITY STATEMENT

The information in this document contains trade secrets and commercial information that is privileged or confidential and may not be disclosed, unless disclosure is required by applicable laws and regulations. In any event, persons to whom the information is disclosed must be informed that the information is privileged or confidential and may not be further disclosed by them. These restrictions on disclosure will apply equally to all future information supplied to you indicated as privileged or confidential.

A Randomized, Double-Blind, Placebo-Controlled, Dose-Escalating, Phase 2a Safety, Tolerability, and Pharmacodynamic Study of Two Doses of an Histone Deacetylase Inhibitor (FRM-0334) in Subjects with Prodromal to Moderate Frontotemporal Dementia with Granulin Mutation

APPROVED BY:

01 - AUG - 14

Hans Moebius, MD, PhD
Vice President Clinical Research Europe
FORUM Pharmaceuticals International B.V.

Date

TABLE OF CONTENTS

CONTACT INFORMATION **6**

INVESTIGATOR AGREEMENT **7**

ABBREVIATIONS **8**

SYNOPSIS **10**

Table 1 Schedule of Study Events for Groups 1 and 2 (FRM-0334-002) **16**

1. INTRODUCTION **18**
1.1 Background 18
1.2 Nonclinical Summary 20
1.3 Clinical Experience 21
1.4 Rationale for Current Study and Study Design 22

2. OBJECTIVES **24**
2.1 Primary Objectives 24
2.2 Secondary Objectives 24
2.3 Exploratory Objectives 24

3. OVERVIEW OF STUDY DESIGN **25**
3.1 Overview 25

4. STUDY POPULATION **28**
4.1 General Considerations and Number of Subjects 28
4.1.1 Support Person Responsibilities 28
4.2 Inclusion Criteria 29
4.3 Exclusion Criteria 30

5. RANDOMIZATION AND BLINDING **33**
5.1 Randomization 33
5.2 Blinding 33
5.3 Premature Unblinding 33

6. DOSE AND STUDY DRUG ADMINISTRATION **34**

7. STUDY DRUG COMPLIANCE **35**

8. PRIOR AND CONCOMITANT MEDICATIONS **35**
8.1 Allowable Medications 35
8.2 Prohibited Medications 36

9. STUDY ASSESSMENTS **36**
9.1 Overview of Study Assessments by Visit 37
9.1.1 Study visits should occur on the designated days, if possible. Screening Period (Days -30 to -8) 37
9.1.2 Screening Period (Day -7 [+3 days]* Visit for Pharmacodynamic Biomarkers) 38
9.1.3 Double-Blind Treatment (Days 1-28), Study Center Discharge (Day 29), Safety Follow-up (Day 38), and Serious Adverse Event Follow-up (Day 58) 38
9.1.3.1 Double-Blind Study Visit on Day 1 38
9.1.3.2 Double-Blind Treatment Study Visits on Days 7 and 14 (±2 days) 39
9.1.3.3 Telephone Contact Visit on Day 21 (±2 days) 40
9.1.3.4 Final Double-Blind Treatment Study Visit on Day 28 and Study Center Discharge on Day 29 40

9.1.3.5 Early Termination Visit ... 41
9.1.3.6 Safety Follow-Up Visits (Days 38 and 58) ... 42
9.1.3.7 Unscheduled Visits ... 42
9.2 Pharmacodynamic Biomarkers ... 43
9.2.1 Plasma Pharmacodynamic Biomarkers and Collection ... 43
9.2.2 Cerebrospinal Fluid Pharmacodynamic Biomarkers and Collection ... 43
9.2.3 18F-Fluorodeoxyglucose Positron Emission Tomography Scan ... 44
9.2.4 Magnetic Resonance Imaging ... 44
9.2.5 Genotyping ... 45
9.3 FRM-0334 Pharmacokinetics ... 45
9.3.1 Plasma Pharmacokinetic Sample Collection and Handling ... 45
9.3.2 Cerebrospinal Fluid Pharmacokinetic Sample Collection and Handling ... 46
9.4 Clinical Rating Scales ... 46
9.5 Safety Assessments ... 49
9.5.1 Medical History and Demographics ... 49
9.5.2 Prior and Concomitant Medications ... 49
9.5.3 Physical Examination ... 49
9.5.4 Vital Signs ... 50
9.5.5 Electrocardiogram ... 50
9.5.6 Clinical Laboratory Tests ... 50
9.5.6.1 Screening Tests ... 50
9.5.6.2 Routine Clinical Laboratory Tests ... 51
9.5.6.3 Total Blood and Cerebrospinal Fluid Volume ... 52
9.5.7 Other Safety Assessments ... 52

10. ADVERSE EVENTS ... 53
10.1 Definitions ... 53
10.1.1 Adverse Events ... 53
10.1.1.1 Protocol-related Adverse Event ... 54
10.1.2 Serious Adverse Events ... 55
10.1.3 Unexpected Adverse Events ... 55
10.2 Evaluation of Adverse Events and Serious Adverse Events ... 56
10.2.1 Severity ... 56
10.2.2 Relationship to Study Drug ... 56
10.2.3 Outcome ... 57
10.2.4 Action Taken Regarding Study Drug ... 57
10.3 Timeframe for Adverse Events and Serious Adverse Events Collection ... 57
10.3.1 General ... 57
10.3.2 Serious Adverse Events Experienced Following Subject Completion of the Study ... 58
10.4 Recording of Adverse Events and Serious Adverse Events ... 58
10.5 Reporting of Serious Adverse Events ... 58
10.6 Follow-Up of Adverse Events and Serious Adverse Events ... 59
10.7 Reporting of Pregnancy ... 60
10.8 Reporting to Institutional Review Boards, Independent Ethics Committees, and Regulatory Authorities ... 60

11. SUBJECT COMPLETION ... 61
11.1 Completion ... 61
11.2 Withdrawal ... 61

12. STATISTICAL METHODS ... 62
12.1 Sample Size Determination ... 62
12.2 Study Subjects ... 62
12.2.1 Analysis Populations ... 62
12.2.2 Subject Disposition ... 63

12.2.3 Demographics and Other Baseline Characteristics 63
12.2.4 Protocol Deviations 63
12.2.5 Prior and Concomitant Medications 63
12.2.6 Study Drug Exposure and Compliance 64
12.3 Pharmacokinetic Analyses 64
12.4 Pharmacodynamic Analyses 64
12.4.1 Primary Endpoint 65
12.4.2 Secondary Endpoints 65
12.5 Exploratory Analyses 65
12.5.1 Clinical Rating Assessments 65
12.5.2 Additional Analyses 66
12.5.2.1 Plasma PGRN 66
12.5.2.2 PD Exploratory Assessments 66
12.6 Safety Analysis 66
12.6.1 Adverse Events 66
12.6.2 Clinical Laboratory Tests 67
12.6.3 Vital Signs 67
12.6.4 Electrocardiogram 67
12.6.5 Other Safety Assessments 68
12.7 Interim Analysis 68

13. STUDY DRUG INFORMATION 69
13.1 Physical Description of Study Drug 69
13.2 Packaging and Labeling 69
13.3 Storage and Return of Study Drug 69
13.4 Drug Accountability 70
13.5 Additional Clinical Supplies 70

14. ETHICAL ASPECTS 71
14.1 Investigator Responsibilities 71
14.2 Institutional Review Board or Independent Ethics Committee 71
14.3 Informed Consent 71
14.4 Confidentiality 72

15. ADMINISTRATIVE REQUIREMENTS 72
15.1 Protocol Modifications 73
15.2 Subject Identification 73
15.3 Record Retention 74
15.4 Case Report Form Completion 74
15.5 Study Completion and Study Termination 75
15.6 Monitoring 75
15.7 Data Quality Assurance 76
15.8 On-Site Audits 76
15.9 Use of Information and Publication 76

16. REFERENCES 78

CONTACT INFORMATION

FORUM Pharmaceuticals Inc. – Sponsor

Main Address:	500 Arsenal Street<br>Watertown, MA 02472 United States

Medical Officer

Name:	Hans Moebius, MD, PhD
Title:	Vice President Clinical Research Europe
Address:	Schipholweg 103, 6th Floor<br>2316XC Leiden<br>The Netherlands
Mobile:	+31 646 135289
Fax:	+1 617 225 4208

Worldwide Clinical Trials - Clinical Research Organization

Medical Monitor

Name:	Manolo Beelke, MD, PhD
Title:	Director Medical Monitoring
Main Address:	Landsberger Str. 63A<br>82110 Germering, Germany
Telephone:	+49 (0) 89 89 05 8422
Fax:	+49 (0) 89 89 05 8423
Mobile:	+49 173 61 89 707

Additional study contact information is included in a study manual, including contact information for reporting a serious adverse event. If any contact information needs to be changed during the study, this will be done with written notification to the investigator, Institutional Review Board (IRB) or Independent Ethics Committee (IEC), and any applicable regulatory authorities, and will not require a protocol amendment.

INVESTIGATOR AGREEMENT

Protocol Title: A Randomized, Double-Blind, Placebo-Controlled, Dose-Escalating, Phase 2a Safety, Tolerability, and Pharmacodynamic Study of Two Doses of an Histone Deacetylase Inhibitor (FRM-0334) in Subjects with Prodromal to Moderate Frontotemporal Dementia with Granulin Mutation

Protocol Number: FRM-0334-002

I have read this protocol and agree that it contains all necessary details for carrying out this study. I will conduct the study as outlined herein and in accordance with current US Food and Drug Administration (FDA) regulations; International Conference on Harmonisation (ICH) guidelines and any other applicable regulatory requirements; as well as Good Clinical Practice (GCP) standards (CPMP/ICH/135/95); the ethical principles that have their origin in the Declaration of Helsinki, and that are consistent with the principles of GCP; all local ethical and legal requirements; and will complete the study within the time designated.

I will provide copies of the protocol and all pertinent information to all individuals responsible to me who assist in the conduct of this study. I will discuss this material with them to ensure they are fully informed regarding the drug and the conduct of the study.

I will use only the Informed Consent Form approved by the Sponsor or its representative and will fulfill all responsibilities for submitting pertinent information to the Institutional Review Board (IRB) or Independent Ethics Committee (IEC) responsible for this study.

I agree that the Sponsor or its representatives shall have access to any source documents from which case report form information may have been generated.

I further agree not to originate or use the name of FORUM Pharmaceuticals Inc. and/or FRM-0334, or any of its employees, in any publicity, news release, or other public announcement, written or oral, whether to the public, press or otherwise, relating to this protocol, to any amendment hereto, or to the performance hereunder, without the prior written consent of FORUM Pharmaceuticals Inc.

____ ________________

Investigator's Signature Date

____

Name of Investigator (typed or printed)

ABBREVIATIONS

Abbreviation	Definition of Term
18F-FDG-PET	18F-fluorodeoxyglucose positron emission tomography
Aβ42	42 amino acid form of β-amyloid
ACE	angiotensin-converting enzyme
ADCS-CGIC	Alzheimer's Disease Cooperative Study – Clinical Global Impression of Change
AE	adverse event
ALT	alanine transaminase
ANCOVA	analysis of covariance
AST	aspartate transaminase
ATC	Anatomical Therapeutic Chemical
AUC	area under the plasma concentration-time curve
$AUC_{(0-t)}$	AUC from time zero to the last quantifiable concentration
C9Orf72	chromosome 9 open reading frame 72 gene
C_{av}	average concentration at steady state
CDR-SB	Clinical Dementia Rating Scale Sum of Boxes (6-item)
CFR	Code of Federal Regulations
CL/F	Apparent clearance following oral administration at steady state
C_{max}	maximum observed plasma concentration
CNS	central nervous system
CSF	cerebrospinal fluid
C-SSRS	Columbia-Suicide Severity Rating Scale
CT	computed tomography
C_{trough}	concentration immediately prior to dosing
CYP	cytochrome P450
DICOM	digital imaging and communications in medicine
DNA	deoxyribonucleic acid
ECG	electrocardiogram
eCRF	electronic case report form
EDC	electronic data capture
EDTA K_2	dipotassium ethylenediaminetetraacetic acid
EU	European Union
ET	early termination
FDA	US Food and Drug Administration
FRS	Frontotemporal Dementia Rating Scale
FTD-CDR-SB	Frontotemporal Dementia Clinical Dementia Rating Scale Sum of the Boxes (8-item)
FTD	frontotemporal dementia
FTLD	frontotemporal lobar degeneration
FTD-GRN	frontotemporal dementia with granulin mutation
GCP	Good Clinical Practice
GRN	granulin gene that encodes progranulin and the granulins
HAT	histone acetyl transferases
HDAC	histone deacetylase
HDACi	histone deacetylase inhibitor
hERG	human ether-a-go-go related gene
IC_{50}	concentration inducing 50% inhibition
ICF	informed consent form
ICH	International Conference on Harmonisation
IEC	Independent Ethics Committee
IRB	Institutional Review Board
IXRS	Interactive Voice/Web Response System
LS	least squares
MedDRA	Medical Dictionary for Regulatory Activities
MMRM	mixed-effects model for repeated measures
MRI	magnetic resonance imaging
mRNA	messenger ribonucleic acid
MTD	maximum tolerated dose

Abbreviation	Definition of Term
n or N	Number
NF-L	neurofilament light chain
NOAEL	no observed adverse effect level
NOR	novel object recognition
NSAIDS	nonsteroidal anti-inflammatory drugs
PD	pharmacodynamic
PGRN	progranulin
PK	pharmacokinetic
p-tau^{181}	tau phosphorylated at threonine 181
QTc	QT interval corrected for heart rate
QTcF	QT interval corrected for heart rate using Fridericia's formula
SAE	serious adverse event
SAHA	suberoylanilide hydroxamic acid
SAP	statistical analysis plan
SOC	system organ class
SUSAR	suspected unexpected serious adverse reaction
$t_{1/2}$	apparent terminal half-life
t-tau	total tau
TEAE	treatment-emergent adverse event
t_{max}	time of the maximum observed plasma concentration
TDP-43	transactive response deoxyribonucleic acid binding protein 43
TSH	thyroid-stimulating hormone
ULN	upper limit of normal
UDS	urine drug screen
WHO	World Health Organization
$\lambda_{z.}$	terminal elimination rate constant at steady state

SYNOPSIS

TITLE

A Randomized, Double-Blind, Placebo-Controlled, Dose-Escalating, Phase 2a Safety, Tolerability, and Pharmacodynamic Study of Two Doses of an Histone Deacetylase Inhibitor (FRM-0334) in Subjects with Prodromal to Moderate Frontotemporal Dementia with Granulin Mutation

PROTOCOL NUMBER

FRM-0334-002

INVESTIGATORS/STUDY CENTERS

This will be a multicenter, multinational study involving approximately 20 sites.

OBJECTIVES

Primary Objectives

- Evaluate the safety and tolerability of 2 fixed doses of FRM-0334 (300 and 500 mg daily in 2 sequential periods) over 28 days in subjects with prodromal to moderate frontotemporal dementia with granulin mutation (FTD-GRN)
- Assess the pharmacodynamic (PD) effects of FRM-0334 on the change from baseline in plasma concentrations of progranulin (PGRN) after 28 days

Secondary Objectives

- Assess the PD effects of FRM-0334 on the change from baseline in cerebrospinal fluid (CSF) concentrations of PGRN after 28 days
- Characterize the plasma and CSF concentrations of FRM-0334 and metabolites following once daily dosing after 28 days
- Characterize the intra- and inter-individual variability in plasma and CSF concentrations of PGRN

Exploratory Objectives

- Assess the effects of FRM-0334 after 28 days on the change from baseline in function using the Frontotemporal Dementia Clinical Dementia Rating Sum of Boxes (FTD-CDR-SB) scale and the Alzheimer's Disease Cooperative Study – Clinical Global Impression of Change (ADCS-CGIC) scale, and activities of daily living using the Frontotemporal Dementia Rating Scale (FRS) in subjects requiring a support person
- Assess the PD effects of FRM-0334 after 28 days on the change from baseline in plasma concentrations of PGRN messenger ribonucleic acid (mRNA) and the CSF concentrations of total tau (t-tau), tau phosphorylated at threonine 181 (p-tau^{181}), 42 amino acid form of β-amyloid (Aβ42), neurofilament light chain (NF-L) and additional CSF biomarkers (eg, transactive response deoxyribonucleic acid [DNA] binding protein 43 [TDP-43]), as technically feasible, and regional cerebral glucose metabolism using 18F-fluorodeoxyglucose positron emission tomography (18F-FDG-PET)

STUDY DESIGN

This is a randomized, double-blind, placebo-controlled, dose-escalating, Phase 2a study to evaluate the safety, tolerability, PD, and pharmacokinetic (PK) effects of FRM-0334 (300 and 500 mg daily during 2 sequential periods with an identical number of subjects per period) for 28 days in subjects with prodromal to moderate FTD-GRN. For this study, the condition of "prodromal" does not require any clinically visible or measurable symptom, but only the presence of the genetic mutation FTD-GRN. Once all Group 1 subjects (300 mg [n=12] or placebo [n=3]) have been randomized and completed (or discontinued) double-blind treatment, safety, tolerability, and any available PK data will be reviewed in a blinded fashion. Randomization of subjects into Group 2 (500 mg [n=12] or placebo [n=3]) will begin only after the 28-day, double-blind safety, tolerability, and available PK data for Group 1 (300 mg FRM-0334 or placebo) subjects are deemed acceptable by the Medical Monitor and Sponsor. Blinded safety, tolerability, and PK data for the 500 mg cohort will be reviewed on an ongoing basis. Enrollment will be competitive. Recruitment will be terminated after randomization of 30 subjects.

NUMBER OF SUBJECTS

Approximately 30 subjects (24 FRM-0334 and 6 placebo) will be randomized during 2 sequential periods. For each period, 15 subjects will be randomized including 12 to FRM-0334 and 3 to placebo.

SUBJECT POPULATION

Inclusion criteria:

1. Informed consent form (ICF) signed by the subject or legally acceptable representative indicating that the subject or legally acceptable representative understands the purpose of and procedures required for the study before any study-specific procedures are performed
2. A support person may be required to participate in the study (in the judgment of the investigator at screening or as required by local regulations). The support person must sign an acknowledgement of responsibilities form at the study center before any study-specific activities required for the support person are performed. The first required activity will be performed at screening. The support person will accompany the subject to the study center at screening and on Days 1 and 28 (or be available by telephone on Days 1 and 28), and if not living in the same household, interacts with the subject approximately 4 times per week, and be able to complete the study
3. Male or female subjects aged ≥21 and ≤75 years
4. Genotyped positive for a FTD-GRN mutation
5. Prodromal to moderate FTD-GRN, and for subjects who require a support person (refer to inclusion criterion No. 2), a Clinical Dementia Rating Sum of the Boxes (CDR-SB) score <16 at screening
6. Fertile, sexually active subjects (men and women) must practice true abstinence or use an effective method of contraception during the study. Female subjects and the female partner of male subjects must be surgically sterile (hysterectomy or bilateral salpingectomy/oophorectomy or bilateral tubal occlusion/ligation), postmenopausal for at least 1 year prior to screening, or willing to consistently and correctly practice adequate methods of contraception if of childbearing potential (defined as consistent use of combined effective methods of contraception [including at least 1 barrier method])
7. Women of childbearing potential must have a negative pregnancy test at screening and Day 1
8. Resides in a stable living situation, living at home, senior residential setting, or an institutional setting without the need for continuous (ie, 24-hour) nursing care
9. Proficiency (oral and written) in the language in which study-related documents, including the ICF and standardized tests, will be administered
10. Able to swallow capsules
11. Be in good general health, willing and able to comply with the protocol requirements, and expected to complete the study as designed (in the judgment of the investigator)

Exclusion Criteria:

Exclusion Criteria – Medical

1. Employees of the investigator or study center or their family members, or employees of FORUM Pharmaceuticals or Worldwide Clinical Trials who are directly involved in the conduct of the study
2. Female subjects who are pregnant, breastfeeding, or planning to become pregnant during the study
3. Unstable medical condition that is clinically significant (in the judgment of the investigator) within 30 days before screening
4. Untreated vitamin B_{12} or folate deficiency (must be stably treated for at least 6 months before screening)
5. Clinically significant untreated hypothyroidism (if treated, thyroid-stimulating hormone level and thyroid supplementation dose must be stable for at least 6 months before screening)
6. Clinically significant abnormal serum electrolytes (sodium, potassium, and magnesium) after repeat testing (in the judgment of the investigator)
7. Alanine transaminase (ALT) or aspartate transaminase (AST) >2.5 times the upper limit of normal
8. Renal insufficiency with serum creatinine >2.0 mg/dL, unless receiving current treatment with an angiotensin-converting enzyme (ACE) inhibitor in which case the Medical Monitor should be contacted
9. Insufficiently controlled diabetes mellitus (in the judgment of the investigator)
10. Clinically significant hematologic abnormalities including thrombocytopenia and leukocytosis (in the judgment of the investigator)
11. Malignant tumor within 3 years before screening with the exception of squamous and basal cell carcinoma or cervical carcinoma in situ or brachytherapy for localized prostate cancer
12. Systemic infection of any kind or any acute, subacute or chronic inflammatory process (eg, rheumatoid arthritis, chronic obstructive pulmonary disease, or gastrointestinal inflammatory diseases)

Exclusion Criteria - Neurological

13. Magnetic resonance imaging (MRI) or computed tomography (CT) scan performed within 12 months

before screening with findings consistent with a clinically significant comorbid pathology other than frontotemporal dementia (FTD). If the MRI or CT scan is unavailable or occurred >12 months before screening, an MRI scan must be completed and findings confirmed before the Day -7 procedures are performed, and a copy of the digital imaging and communications in medicine (DICOM) standard image and report must be available

14. Diagnosis of motor neuron disease, including probable amyotrophic lateral sclerosis
15. History of brain tumor, subdural hematoma, or other clinically significant (in the judgment of the investigator) space-occupying lesion on CT or MRI
16. Stroke within 18 months before screening or history of a stroke concomitant with onset of dementia
17. Head trauma with clinically significant (in the judgment of the investigator) loss of consciousness within 12 months before screening or concurrent with the onset of dementia
18. Onset of dementia within 12 months before screening secondary (in the judgment of the investigator) to cardiac arrest, surgery with general anesthesia, or resuscitation
19. Specific degenerative central nervous system (CNS) disease diagnosis other than FTD (eg, Parkinson's disease, Alzheimer's disease, Huntington's disease, Creutzfeldt-Jakob disease, Down's syndrome)
20. Wernicke's encephalopathy
21. Epilepsy if present antiseizure therapy is required for seizure control

Exclusion Criteria – Psychiatric

22. Current diagnosis of severe major depressive disorder with psychotic features, if the present condition or treatment interferes with the subject's ability to complete the study (in the judgment of the investigator)
23. Significant suicide risk as defined by 1) suicidal ideations as endorsed on items 4 or 5 on the Columbia-Suicide Severity Rating Scale (C-SSRS) within the past year at screening or baseline, 2) suicidal behaviors detected by the C-SSRS within 2 years before screening, or 3) investigator assessment
24. History or current diagnosis of psychosis
25. History within 2 years before screening or current evidence of substance abuse as defined by the Diagnostic and Statistical Manual of Mental Disorders 4th Edition Text Revision
26. Clinically significant urine drug screen (UDS) or serum alcohol test result (in the judgment of the investigator) at screening (may be repeated once)

Exclusion Criteria - Cardiovascular

27. Clinically significant abnormality on screening or baseline electrocardiogram (ECG), including but not necessarily limited to a confirmed corrected QT interval corrected for heart rate (QTc) value ≥450 msec for males or ≥470 msec for females. In subjects with a QRS value >120 msec those with a QTc value >480 msec are NOT eligible
28. History of myocardial infarction or unstable angina within 6 months before screening or history of >1 myocardial infarction within 5 years before screening
29. Clinically significant (in the judgment of the investigator) cardiac arrhythmia (including atrial fibrillation), cardiomyopathy, or cardiac conduction defect (subjects with a pacemaker are acceptable)
30. Cardiovascular disease history including symptomatic hypotension or hypertension (supine diastolic blood pressure >95 mmHg) not stabilized by medical therapy (in the judgment of the investigator)

Exclusion Criteria – Prohibited Prior and Concurrent Medications

31. Investigational drug, biologic or medical device within 30 days before screening or planning to use an investigational drug (other than FRM-0334) during the study
32. Sensitive and/or narrow therapeutic range cytochrome P450 (CYP) 1A2 substrates including alosetron, duloxetine, melatonin, ramelteon, theophylline and tizanidine
33. Warfarin-derived anticoagulants, heparin, NSAIDs, and anti-platelet drugs (clopidogrel and others) 10 days before the CSF sample collection on Day -7 and Day 28
34. Hypersensitivity to histone deacetylase inhibitor (HDACi) medication or currently receiving treatment with an HDACi, including valproic acid and amisulpride
35. Immunosuppressants including systemic corticosteroids and nonsteroidal anti-inflammatory drugs (NSAIDs) (Note: occasional use of NSAIDs is allowed, with the exception of 10 days before the CSF sample collection on Day -7 and Day 28)
36. Chronic intake of opioid containing analgesics
37. Psychostimulants, including amphetamines
38. Clozapine and amisulpride; other antipsychotics are allowed if on a stable dose (no change in the dose or frequency) for at least 30 days before screening and expected to remain stable during the study

DOSE/ROUTE/REGIMEN

Once daily oral dosing of 300 or 500 mg FRM-0334 as a white, opaque, size No. 1, hard gelatin capsule for 28 days, supplied as a 100 mg capsule that will be size- and color-matched to the placebo capsule. Subjects will be instructed to ingest study drug (3 capsules daily for Group 1 and 5 capsules daily for Group 2) with food and water in the morning at approximately the same time each day.

REFERENCE TREATMENT

Once daily oral dosing of placebo for 28 days, supplied as a capsule that will be identical in appearance to the 100 mg FRM-0334 capsule. Subjects will be instructed to ingest study drug (3 capsules daily for Group 1 and 5 capsules daily for Group 2) with food and water in the morning at approximately the same time each day.

DURATION OF TREATMENT

Expected duration of the study for each subject is approximately 88 days. This includes up to 30 days of screening (Days -30 to -8) and Day -7 (PD biomarkers and 18F-FDG-PET performed within approximately 7 days of study drug dosing on Day 1); 28 days of double-blind treatment (Days 1 to 28); 10 days of follow-up (Day 38); and SAE telephone contact at 30 days after the last dose. On Day 1, subjects will remain in the study center until all scheduled procedures have been completed (approximately 8 hours postdose), and on Day 28, subjects will return to the study center. Assessments will be performed through Day 29, after which subjects will be discharged. Maximum duration of study drug exposure for any subject is 28 days.

METHODOLOGY

Site personnel (raters) will be trained in the administration and scoring of the C-SSRS, CDR-SB, FTD-CDR-SB, FRS, and ADCS-CGIC. C-SSRS is required by all subjects. For those subjects requiring a support person in the judgment of the investigator, the rater will administer and score the CDR-SB (screening only), FTD-CDR-SB, FRS, and ADCS-CGIC, using information collected during interviews.

Study centers will have adequately equipped and trained personnel (or access to adequate equipment and trained personnel) to perform lumbar punctures for CSF collection and 18F-FDG-PET scanning technology. The 18F-FDG-PET scan will be uploaded to a secure, central server, and the blinded, external 18F-FDG-PET reviewers will access the central server to assess the standardization and quality of the scans and evaluate the findings. Diagnostic MRI or CT will be assessed at the study center, if applicable. For anatomical mapping of the 18F-FDG-PET imaging analysis, each subject must have an MRI scan with DICOM image available. The MRI scan with DICOM file must be available within 12 months of screening or by the end of the Day 14 (±2 days) visit.

Screening (Days -30 to -8): Subjects (or legally acceptable representative) and support person (if applicable) will provide informed consent and acknowledgement of responsibilities, respectively, before any study-specific procedures are performed. Subjects will be screened for eligibility, as shown in Table 1. Each subject will be assessed, and those who are cognitively and functionally self-sufficient in the judgment of the investigator at screening are not required to have a support person. Subjects who are required to have a support person, in the judgment of the investigator, will have the CDR-SB performed at screening (score <16 is required for study entry).

Screening (Day -7 for the PD Biomarkers and Randomization): In the morning of Day -7 (or within approximately 7 days prior to the first dose of study drug on Day 1), eligible subjects will have procedures performed for the plasma and CSF PD biomarkers, CSF PK for FRM-0334 and metabolites, and the 18F-FDG-PET scan. Subjects will undergo lumbar puncture for the CSF sample collection, and they will be required to remain supine for approximately 2 hours afterwards, or as dictated by local practice. A CSF sample will also be sent for safety laboratory testing. Anxious and/or agitated subjects may receive a sedative in order to undergo the procedure if needed in the judgment of the investigator. This visit may be extended for up to 3 days, after consultation with the Medical Monitor, in order to schedule the CSF sample collection or the 18F-FDG-PET scan.

Subjects who do not qualify for the study based upon the screening criteria and the investigator's assessment regarding subjects' ability to complete all study procedures, including 18F-FDG-PET and lumbar puncture for the CSF sample collection, will be discontinued.

Predose on Day 1 (Baseline), Double-Blind Treatment (Days 1 to 28), and Study Center Discharge (Day 29): In the morning of Day 1 (predose), baseline assessments will be performed to confirm eligibility for continued study participation and study drug dosing, as shown in Table 1. Eligible subjects will be randomized

in the Interactive Voice/Web Response System (IXRS) to receive FRM-0334 or placebo during 2 sequential periods (Group 1: 300 mg or placebo; Group 2: 500 mg or placebo). A 1-week supply of study drug will be dispensed on Days 1 and 7, and a 2-week supply will be dispensed on Day 14; subjects will be instructed to ingest study drug once daily, in the morning at approximately the same time each day, with food and water.

On Day 1, subjects will ingest the first dose of study drug with food and water in the presence of study center personnel after the predose blood sample collection for the PK and PD analysis and laboratory tests, including the urine pregnancy test for women of childbearing potential, have been obtained. All additional doses will be ingested as outpatients, with the exception of study visits on Days 7, 14, and 28, at which study drug will be ingested in the presence of study center personnel. On these days, subjects will return all remaining study drug supplies to study center personnel.

On Day 21 (±2 days), subjects will be contacted by telephone for a safety evaluation (adverse events [AEs], concomitant medications, and suicidality using the C-SSRS). On Day 28, subjects will return for the final double-blind visit. Last dose of study drug will be ingested in the clinic on Day 28; maximum duration of study drug dosing for any subject is 28 days. If needed, subjects may have the 18F-FDG-PET scan only performed on Day 27 ideally in the afternoon, but the scan should not be performed on Day 29

On Day 1, subjects will be required to remain in the study center for approximately 8-10 hours postdose or until all study-specific procedures have been completed. On Day 28, subjects will return to the study center. Assessments will be performed through Day 29, after which subjects will be discharged.. Subjects should bring all applicable concomitant medications to the study visit on Days 1, 7, 14 and 28, and study center personnel may administer these medications to subjects at the appropriate times.

Refer to Table 1 for study assessments that will be completed during double-blind treatment.

Early Termination (ET) Visit: Subjects who prematurely discontinue double-bind treatment will be encouraged to return to the clinic for an ET visit. Refer to Table 1 for ET assessments.

Safety Follow-up: Subjects who complete double-blind treatment will return to the clinic for a safety follow-up visit on Day 38 (+2 days). Refer to Table 1 for safety follow-up assessments. Additional safety assessments (eg, ECG, clinical laboratory tests) may be performed as requested by the investigator.

SAE Follow-up: All subjects, including those who prematurely discontinue, will be contacted by telephone to assess any serious AE (SAE) experienced within 30 days (+2 days) after the last dose of study drug.

Any treatment-emergent clinically significant abnormalities persisting at the end of the study or ET will be followed until resolution or until reaching a clinically stable endpoint.

CRITERIA FOR EVALUATION

Safety Assessments: AEs, clinical laboratory tests (serum chemistry, hematology and urinalysis), vital signs including orthostatic measurements of pulse and blood pressure, 12-lead triplicate ECG, physical examination, and C-SSRS

Pharmacodynamic Biomarkers: Plasma PGRN and PGRN mRNA concentrations, CSF PGRN, t-tau, p-tau[181], Aβ42, and NF-L concentrations (additional CSF biomarkers including TDP-43, as technically feasible), and regional cerebral glucose metabolism using 18F-FDG-PET

Pharmacokinetics: Plasma concentrations and PK parameters and CSF concentration data for FRM-0334 and metabolites

STATISTICAL METHODOLOGY

Statistical analyses will be conducted for the safety, PD, and PK data using appropriate methods. Final analyses will be provided using both groups (Group 1: 300 mg or placebo; Group 2: 500 mg or placebo). A detailed statistical analysis plan (SAP) will be prepared for the final analyses before database lock.

Descriptive statistics will be presented for all analyses unless otherwise specified. For continuous variables, data will be presented as number (n), mean, median, standard deviation, minimum, and maximum. Discrete variables will be presented as frequencies and proportions or percentages. For most summary statistics, data will be analyzed by treatment group (300 and 500 mg FRM-0334 and placebo).

Sample Size Estimation: Sample sizes of 15 subjects per period (n=12 FRM-0334, n=3 placebo) will provide approximately 80% power to detect a 30% increase in plasma PGRN concentration from baseline (based on a 1-sided test, alpha=0.05).

Primary and Secondary Endpoints: The primary hypothesis being tested is that treatment with FRM-0334, 300 or 500 mg daily, increases mean plasma PGRN concentrations (1-sided test, alpha=0.05; no correction for

multiplicity).

The primary PD analysis will be the percent change from baseline to Day 28 in plasma PGRN concentration for FRM-0334 treatment, using a mixed-effects model for repeated measures (MMRM), with fixed effects for treatment, time, treatment by time interaction, baseline as a covariate, and subject as a random effect. Analysis of PGRN mRNA will be performed using a similar method.

Secondary PD endpoints include: the effect FRM-0334 on plasma, PGRN, and PGRN mRNA (comparing Day 1 and 28 postdose values to respective predose values, as well as to baseline); and the percent change from baseline to Day 28 in CSF concentration of PGRN, data will be listed and summarized in tabular format by descriptive statistics as appropriate.

Exploratory Endpoints: The exploratory PD, PK, and clinical ratings scales data will be analyzed in a similar manner to the primary analysis as described above.

Safety Analysis: Descriptive statistics will be used to analyze the safety data using the safety population (all subjects who ingested at least 1 dose of study drug).

TABLE 1 SCHEDULE OF STUDY EVENTS FOR GROUPS 1 AND 2 (FRM-0334-002)

	Screening[a]		Double-Blind Treatment - FRM-0334 (300 or 500 mg daily) or Placebo									Follow-Up	
**Visit**	**1**	**2**	**3**		**4**	**5**	**6**	**7**				**8**	**9 (SAE)[e]**
**Study Day (Visit Window, if applicable)**	**-30 to -8**	**-7[b]**	**1**		**7 (±2)**	**14 (±2)**	**21 (±2)**	**28**		**29**	**ET[c]**	**38[d] (+2)**	**58 (+2)**
**Event/Assessment**			**Pre Dose/ Baseline**	**Post Dose**				**Pre Dose**	**Post Dose**				
Subject informed consent form (ICF)	X												
Support person acknowledgement of responsibilities, if applicable	X												
Medical history and demographics	X												
Inclusion/exclusion criteria[b]	X	X	X										
Randomization			X										
MRI or CT scan[f]	X												
Clinical Dementia Rating Sum of Boxes (CDR-SB)[g]	X												
ADCS-CGIC, FTD-CDR-SB, and FRS[g]			X						X				
Columbia-Suicide Severity Rating Scale (C-SSRS)	X		X		X	X	X		X		X	X	
Clinical laboratory tests[h]	X		X		X	X		X			X	X	
Plasma PK sample for FRM-0334 and metabolites[i]			X	X	X	X		X	X	X			
Plasma PD sample for PGRN and PGRN mRNA[i]	X	X	X	X	X	X		X	X	X	X	X	
18F-FDG-PET scan[j]		X							X				
CSF sample for safety, PGRN, t-tau, p-tau[181], Aβ42, & NF-L (PD analysis), and FRM-0334 & metabolites concentration data[k]		X							X				
Blood sample for genotyping[l]			X										
Vital signs measurements[m]	X	X	X	X	X	X		X	X	X	X	X	
12-lead triplicate electrocardiogram (ECG)[n]	X		X	X	X	X			X		X		
Physical examination[o]	X		X		X	X			X		X		
Body weight	X		X						X		X	X	
Pregnancy test in females (childbearing potential)[p]	X		X		X	X		X			X		
Urine drug screen (UDS) and serum alcohol test[q]	X												
Study drug dispensed[r]			X		X	X							
Study drug administered in the clinic[r]			X		X	X		X					
Study drug accountability/compliance[r]				X	X	X			X		X		
Prior/concomitant medications review[s]	X	X	X		X	X	X	X		X	X	X	
Adverse event (AE) monitoring[s]	X	X	X		X	X	X	X		X	X	X	
Telephone contact							X						X

Abbreviations: Aβ42=42 amino acid form of β-amyloid; ADCS-CGIC=Alzheimer's Disease Cooperative Study – Clinical Global Impression of Change; CSF=cerebrospinal fluid; CT = computed tomography; ET=early termination; 18F-FDG-PET=18F-fluorodeoxyglucose positron emission tomography; FRS=Frontotemporal Dementia Rating Scale; FTD-CDR-SB= Frontotemporal Dementia Clinical Dementia Rating Sum of the Boxes; MRI = magnetic resonance imaging; mRNA=messenger ribonucleic acid; NF-L=neurofilament light chain; PD=pharmacodynamic; PGRN=progranulin; PK=pharmacokinetic; p-tau[181]= tau phosphorylated at threonine 181; SAE=serious adverse event; t-tau=total tau..

Note: Footnotes are located on the next page.

Table 1 Schedule of Study Events (continued)

a. Screening and the baseline visits cannot occur on the same day.
b. Subjects will be assessed for inclusion and exclusion criteria (eligibility) during screening and confirmed on Day -7 and predose on Day 1; Day -7 procedures will be completed within approximately 7 days of study drug dosing on Day 1. The Day -7 visit may be extended for up to 3 days, after consultation with the Medical Monitor, in order to schedule the CSF sample collection or the 18-FDG-PET scan.
c. An ET visit will be performed for subjects who discontinue double-blind treatment.
d. A safety follow-up clinic visit (Day 38) is required for subjects who complete double-blind treatment.
e. A follow-up telephone contact (Day 58) is required for all subjects to assess any SAE experienced within 30 (+2) days after the last dose of study drug.
f. An MRI or CT is required within 12 months of screening to confirm pathology. Findings must be confirmed before Day -7 procedures. For anatomical mapping of the 18F-FDG-PET imaging analysis, each subject must have an MRI scan with DICOM image available. The MRI scan with DICOM file must be available within 12 months of screening or by the end of the Day 14 (±2 days) visit.
g. The CDR-SB will be performed at screening for subjects required to have a support person in the judgment of the investigator; ADCS-CGIC, FTD-CDR-SB, and FRS assessments will be performed only for subjects required to have a support person. For Day 1 (predose), the rater will interview the subject (first) and support person using the ADCS-CGIC source workbook.
h. Predose hematology, serum chemistry, and urinalysis will be performed at screening, on Days 1, 7, 14, and 28, or at ET . Thyroid-stimulating hormone, serum vitamin B_{12}, and serum folate will be performed at screening only. Prothrombin time will be assessed at screening. If a subject takes an anticoagulant at any point during the study, prothrombin time should also be assessed at Day 28 (prior to CSF collection). For urinalysis local urine dipstick analysis will be completed. If positive and considered clinically significant by the investigator, a urine sample will be sent to the central laboratory for microscopic analysis. Clinical laboratory tests will be performed on Day 38 only if clinically relevant changes from baseline are observed on Day 28.
i. On Day 1, a PK and PD sample will be collected predose and 1, 2, 4, 6, and 8 hours postdose; an optional 10 hour postdose PK and PD sample may be collected. On Days 7 and 14, a predose PK and PD sample will be collected. On Day 28, a PK and PD sample will be collected predose and 1, 2, 4, 6, 8, 10, 12 and 24 hours postdose (before discharge on Day 29); an optional 16 hour postdose PK and PD sample may be collected.
j. 18-FDG-PET scan performed on Day 28 postdose will ideally be in the afternoon, but before the CSF sample collection (or if needed, the scan only may be performed on Day 27 ideally in the afternoon, but not on Day 29).
k. CSF sample collection on Day 28 at approximately 7-10 hours postdose. On Day -7 and Day 28, subjects will undergo lumbar puncture for the sample collection, and they will be required to remain supine for at approximately 2 hours afterwards, or as dictated by local practice; samples will be sent for clinical laboratory safety testing.
l. Blood sample for future research to develop a method to detect GRN mutations. The blood sample may also be used for genotyping of drug-metabolizing enzymes. Subjects will indicate on the ICF if they will allow a blood sample to be reserved and used for future research.
m. Vital signs measurements include orthostatic measurements of pulse and supine and standing blood pressure obtained supine (after 5 minutes) and upon standing (after 2 minutes) and supine (after 5 minutes) respiratory rate and body temperature. On Day 1, measurements will be obtained predose and 4 and 6 hours postdose; on Days 7 and 14, measurements will be obtained predose and 4 and 6 hours postdose; and on Day 28, measurements will be obtained predose and 4, 6, and 24 hours postdose (or before discharge on Day 29).
n. ECG will be conducted in triplicate (1 to 3 minutes apart after the subject has rested supine for at least 5 minutes) at predose and 4 hours postdose on Day 1, 4 hours postdose on Days 7 and 14, and 4 hours postdose on Day 28. At predose on Day 1, the ECG must be reviewed by a physician at the study center before the subject is dosed.
o. A complete physical examination will be performed at screening that will include a brief neurological examination and height; a directed physical examination will be performed at all other scheduled visits or ET.
p. Local urine pregnancy dipstick test (females of childbearing potential) will be performed. If positive, a serum pregnancy test will be performed (except on Day 1 predose) by a central laboratory and must be negative in order for the subject to continue study participation.
q. If positive, the urine drug screen and serum alcohol test may be repeated once.
r. Study drug will be dispensed on Days 1, 7, and 14. The first dose will be ingested with food and water in the clinic on Day 1 after all predose assessments are completed and eligibility has been confirmed. Additional doses will be ingested as outpatients, with the exception of study visits on Days 7, 14, and 28. On these days, subjects will ingest study drug with food and water in the presence of study center personnel after the sample collection for the PK and PD analysis and laboratory tests, including the urine pregnancy test for women of childbearing potential has been obtained. Subjects will be instructed to ingest study drug once daily, in the morning at approximately the same time each day, with food and water; they will be asked to return any remaining study drug supplies at visits on Days 7, 14, and 28 or ET.
s. Adverse events and concomitant medications (30 days prior to screening and current) will be recorded after informed consent is obtained at screening until Day 38 or ET.

Notes: For all subjects, 28 days is the maximum number of days of dosing allowed.

1. INTRODUCTION

1.1 Background

The potential clinical indication for FRM-0334 is the treatment of a subset of inherited frontotemporal dementia (FTD) that is due to mutations in the granulin (GRN) gene (FTD-GRN). FTD-GRN is part of the heterogenous frontotemporal lobar degeneration (FTLD) syndrome. The various disease entities of the FTLD syndrome have an early onset (<65 years) and account for 5 to 10% of all dementia and 10 to 20% of early onset dementia (Eriksen and Mackenzie, 2008).

In general, FTD is characterized by changes in personality, cognition (eg, language impairment and executive dysfunction), and behavior (eg, disinhibition, apathy, and compulsivity) (van Swieten and Heutink, 2008). Common FTLD syndromes include: behavioral variant FTD, semantic dementia, progressive apraxia of speech, agrammatic aphasia, progressive supranuclear palsy syndrome, cortico-basal syndrome, amyotrophic lateral sclerosis with dementia, FTD with motor neuron disease, and other less common syndromes. In particular, the clinical phenotype of FTD-GRN is highly variable. The clinical phenotype is usually a combination of behavioral abnormalities and language disturbances that is most often a form of primary progressive aphasia. Mild Parkinsonism is common, but motor neuron disease is notably rare. Marked variation in the disease course and clinical features are common, not only between families with different mutations, but also within individual families. This degree of clinical variability makes it difficult to predict which cases of familial FTD will turn out to have a GRN mutation (Mackenzie, 2007).

Approximately 10 to 50% of FTD cases are inherited in an autosomal dominant fashion, with mutations in several genes causing pathogenesis (Eriksen and Mackenzie, 2008; Gass et al, 2012). These include mutations in the genes encoding microtubule-associated protein tau, progranulin (PGRN), valosin containing protein, charged multivesicular body protein 2B, transactive response deoxyribonucleic acid (DNA) binding protein 43 (TDP-43), and fused in sarcoma, and most recently in the repeat expansion of the chromosome 9 open reading frame 72 gene (C9Orf72) (Dobson-Stone et al, 2012).

Neuropathologically, the FTLD syndrome can be divided into 2 major groups that have a clear correlation with their genetic background, including those with tau-positive inclusions and those with ubiquitin- and TDP-43-positive inclusions. Recently, mutations in GRN on chromosome 17q21 were found to cause an FTLD variant with ubiquitin- and TDP-43-positive inclusions (Cruts et al, 2006; Baker et al, 2006; Gass et al, 2012). Seventy pathogenic GRN mutations in over 230 families have been described for FTD-GRN to date. These mutations include frameshift, splice-site, and nonsense mutations that are predicted to produce a premature stop codon. The mutations result in haploinsufficiency and a greater

than 50% reduction in expression of PGRN in plasma (Ghidoni et al, 2012). At present, there are no drugs available to treat GRN haploinsufficiency and the resulting neurodegeneration and dementia of FTD-GRN.

In the adult brain, GRN messenger ribonucleic acid (mRNA) and PGRN immunoreactivity are located in certain neuronal populations (eg, pyramidal cells of cortex and hippocampus and cerebellar Purkinje cells) and in microglia (Eriksen and Mackenzie, 2008). In neuroinflammatory and neurodegenerative diseases, microglia appear to produce and secrete the majority of PGRN, which is taken up from the extracellular space by neurons (Sun and Eriksen, 2011). In FTD-GRN, GRN haploinsufficiency and decreased PGRN levels may result in an exaggerated neuroinflammatory response and/or loss of neurotrophic activities, leading to neurodegeneration.

A suggested approach for the treatment of FTD-GRN caused by GRN haploinsufficiency has been to increase PGRN levels. The histone deacetylase (HDAC) inhibitor (HDACi) suberoylanilide hydroxamic acid (SAHA) increased GRN mRNA transcription and PGRN levels (Cenik et al, 2011). While SAHA increased PGRN levels, it has limited central nervous system (CNS) penetration.

In the cell nucleus, DNA is tightly wound around proteins called histones, and this DNA/histone association is called chromatin (de Ruijter et al, 2003). Gene transcription depends on how tightly histones and DNA are associated; a looser association promotes gene transcription. Acetylation of histones results in a looser association of histone with DNA and therefore increases gene transcription. Addition of an acetyl group onto histones is catalyzed by a family of enzymes called histone acetyl transferases (HATs). Removal of acetyl groups from histones, with subsequent decreases in gene transcription, is catalyzed by a family of enzymes called HDACs. Thus inhibition of HDACs will result in retention of acetyl groups on histones, thereby maintaining a loose association between histone and DNA and increasing gene transcription. Although histone deacetylation is the best understood function of HDACs, other proteins in the cell can be deacetylated (Spange et al, 2009).

Furthermore, several lines of evidence indicate that HDACi may be useful for the treatment of memory loss in patients with dementia (Tully et al, 2003; Sweatt, 2007; Abel and Zukin, 2008; Fischer et al, 2010). In a transgenic mouse model that displays substantial atrophy in brain regions involved in cognition and memory, such as the hippocampus and cerebral cortex, HDACi restored memory function tested in associative learning and spatial learning memory tasks (Fischer et al, 2007). These results implicate HDACi as potential treatments for cognitive disorders that arise from neurodegeneration (Sweatt, 2007).

In conclusion, the effects of SAHA on GRN mRNA and PGRN levels suggest that a brain penetrant HDACi may be useful for treating FTD-GRN. FRM-0334 is a brain penetrant

HDACi and has shown the potential to elevate GRN mRNA and PGRN levels in a variety of *in vitro* and *in vivo* models relevant to FTD-GRN due to GRN haploinsufficiency and to enhance cognition in animal models. Addressing the GRN haploinsufficiency associated with GRN mutations by increasing PGRN expression may offer a method to potentially treat these patients.

1.2 Nonclinical Summary

Nonclinical Pharmacology: FRM-0334 was evaluated in numerous *in vitro* and *in vivo* models of HDAC activity, and is an HDACi with high affinity for several closely related HDAC subtypes.

Several studies have evaluated the effects of FRM-0334 on GRN mRNA and PGRN levels in rodents. In the cerebral cortex of young, male mice, a single 100 mg/kg oral dose of FRM-0334 significantly increased GRN mRNA by 1.13- to 1.39-fold ($p<0.01$) up to 8 hours postdose compared with control. The effects of age and FRM-0334 treatment on GRN mRNA levels in mice were studied. GRN mRNA levels in the frontal cortex of 42-week-old mice vs. 9-week-old mice were reduced by 23% ($p<0.05$). At 8 hours postdose, a single, oral 100 mg/kg dose of FRM-0334 significantly increased GRN mRNA in 41 to 43-week-old mice vs. age-matched, vehicle-treated mice by 17% ($p<0.05$) in the frontal cortex and 42% ($p<0.001$) in the hippocampus.

Overall, *in vitro* and *in vivo* acetylation data suggest that FRM-0334 induces acetylation in CNS and peripheral tissues at total plasma concentrations of about 1 to 2.5 μM. This corresponds to approximately 30 to 75 nM (10 to 25 ng/mL) of free drug in human plasma based on 97% protein plasma binding of FRM-0334.

Several studies evaluated the effects of FRM-0334 in animal models of cognition. In the Morris water maze memory task in mice, there was a trend toward improved performance on the hidden platform task with FRM-0334, especially with the higher dose (30 mg). In the novel object recognition (NOR) task, FRM-0334 significantly increased exploration of the unfamiliar object at 1.5 and 24 hours after single oral dosing of 10 and 3 mg/kg, respectively, suggesting that FRM-0334 enhanced both short- and long-term memory in mice.

Nonclinical Pharmacokinetics (PK): In the mouse and dog PK studies, FRM-0334 was rapidly cleared from plasma with a mean apparent terminal half-life ($t_{1/2}$) value of approximately 0.65 to 1.9 hours with a moderate to high volume of distribution at steady-state. Oral bioavailability was 38% in mice and 45% in dogs. Mean time to occurrence of the maximum observed plasma concentration (t_{max}) values ranged from 0.25 to 1.6 hours. Protein-binding studies suggest that FRM-0334 is highly bound to plasma proteins (~97 to 99%) for all species tested. Metabolism studies demonstrated that FRM-0334 had high intrinsic clearance in human, rat, dog, and mouse hepatocytes, and

moderate intrinsic clearance in monkey hepatocytes. Two potential metabolites were observed in human hepatocytes, both of which were observed in dog hepatocytes and one of which was observed in mouse hepatocytes. FRM-0334 was a moderately potent inhibitor of cytochrome P450 (CYP) 1A (concentration inducing 50% inhibition [IC_{50}]: 3.84 µM) and a weak inhibitor (IC_{50} >25 µM) of other evaluated CYP isozymes (CYP2C9, CYP2C19, CYP2D6, and CYP3A4). No CYP induction studies with FRM-0334 have been performed.

Nonclinical Toxicology: FRM-0334 had no noteworthy effect in *in vivo* cardiovascular and CNS safety pharmacology studies and the FRM-0334 IC_{50} value for inhibition of the human ether-a-go-go related gene (hERG) current was >20 µM. The maximum tolerated doses (MTDs) following a single oral dose of FRM-0334 in mice and dogs were 1000 mg/kg and 500 mg/kg, respectively. The no observed adverse effect level (NOAEL) in the pivotal 28-day repeat-dose study in mice was 50 mg/kg/day, based on reversible changes in hematology (primarily decreased leukocyte count) and clinical chemistry (primarily increased triglycerides) profiles and decreased thymic organ weights. Reversible increased liver weight and structural changes in the liver were also observed, but these were considered adaptive and non-adverse. The NOAEL in the pivotal 28-day repeat-dose study in Beagle dogs was 100 mg/kg/day, based on a reversible decrease in food consumption and corresponding decrease in body weight gain, and reversible microscopic lesions in multiple organs at 250 mg/kg/day. Area under the plasma concentration-time curve (AUC) based therapeutic index in mice was 2.3, using the lowest observed exposure at the NOAEL (1670 h•ng/mL; 50 mg/kg; Day 28; female) and exposure for induction of global striatal histone acetylation (731 h•ng/mL; 10 mg/kg; Day 0; female).

1.3 Clinical Experience

Safety Effects in Humans: One Phase 1 study with FRM-0334, in 87 healthy male and female subjects (18-65 years), was completed; 70 subjects received FRM-0334 (10 to 400 mg) and 17 subjects received placebo (EVP-0334-001).

During the single-ascending phase, subjects received a single-dose of 10 (fasted), 20 (fasted), 50 (fasted), 100 (fed and fasted), 200 (fasted), or 400 mg (fed) FRM-0334 or placebo during separate admission periods with a washout of at least 7 days between doses. During the multiple-ascending dose phase, subjects received 10, 20, 50, 200 mg or 400 mg doses of FRM-0334 or placebo under the fed condition once daily for 14 days.

During the single-dose phase, the only individual treatment-emergent adverse events (TEAEs) experienced by more than 1 subject dosed with FRM-0334 were headache and myalgia. Headache was experienced by 2 (40%) subjects after the 20 mg (fasted) dose and 2 (33%) subjects after the 100 mg (fasted) dose vs. one (8%) subject after placebo, and

myalgia was experienced by 2 (33%) subjects after the 400 mg (fed) dose vs. no subject after placebo.

During the multiple-dose phase, the most common TEAE across the FRM-0334 groups was headache experienced by 5 (50%) subjects in the 400 mg group vs. 2 (13%) subjects in the placebo group, 2 (22%) in the 50 mg group, 1 (11%) per group in the 10 and 20 mg groups, and no subject in the 200 mg group. Headache was the only TEAE that appeared to be related to increasing dose of FRM-0334.

No serious TEAE or event rated as severe was experienced, while one subject after dosing with placebo discontinued the single-dose phase due to bursitis.

Pharmacokinetics in Humans: The PK properties of FRM-0334 and the metabolite, FRM-0334 glucuronide, were determined after single- and multiple doses of 10 to 400 mg. Maximum observed plasma concentration (C_{max}) of FRM-0334 and total systemic exposure (AUC) increased approximately proportional to dose over the 10 to 400 mg single- and multiple-dose range, while there was a less than dose-proportional increase observed for FRM-0334 glucuronide. Median maximum plasma concentrations for FRM-0334 were reached on average between 2.5 and 4 hours postdose; similar results were observed for FRM-0334 glucuronide.

The geometric mean elimination $t_{1/2}$ values ranged between 3.5 and 5.2 hours during the single-dose phase and between 4.6 and 10.4 hours during the multiple-dose phase, and $t_{1/2}$ was independent of dose. Slightly greater $t_{1/2}$ values were observed for FRM-0334 glucuronide. Absorption of FRM-0334 was substantially enhanced with food.

Renal excretion of FRM-0334 and FRM-0334 glucuronide was only a minor contributor to the elimination of orally administered FRM-0334.

Additional information is provided in the Investigator's Brochure.

1.4 Rationale for Current Study and Study Design

This placebo-controlled, double-blind, 28-day, Phase 2a study is a proof of mechanism study designed to provide preliminary evidence of the safety, tolerability, pharmacodynamic (PD) and PK effects in plasma and cerebrospinal fluid (CSF) of FRM-0334 in subjects with prodromal to moderate FTD-GRN. For this study, the condition of "prodromal" does not require any clinically visible or measurable symptom, but only the presence of the genetic mutation FTD-GRN. Subjects will receive FRM-0334 or placebo during 2 sequential periods (Group 1: 300 mg or placebo; Group 2: 500 mg or placebo [identical number of subjects per period]) for 28 days. Randomization of subjects into Group 2 (500 mg or placebo) will begin only after the blinded 28-day double-blind, safety, tolerability, and available PK data for Group 1 (300 mg or placebo) are deemed acceptable by the Medical Monitor and FORUM

Pharmaceuticals Inc. (ie, the Sponsor). Blinded safety, tolerability, and PK data for the 500 mg cohort will be reviewed on an ongoing basis.

Currently, no drugs are approved for the treatment of FTD or the various disease entities, including FTD-GRN. In the absence of effective treatments, development of medications to treat FTD-GRN is a high public health priority.

The current study is the first study of FRM-0334 in subjects with prodromal to moderate FTD-GRN. Subjects' safety will be closely monitored and exposure will be limited to 28 days. The study design includes a clinic follow-up safety visit approximately 10 days after the last dose of study drug and a telephone assessment for the occurrence of any serious adverse events (SAEs) approximately 30 days after the last dose of study drug. This is a sequential dose escalation study.

Two doses of FRM-0334 will be studied during 2 dosing periods (28 days each) of identical study design. Group 1 subjects will be randomized first and receive 300 mg FRM-0334 or placebo. After all subjects in Group 1 have been randomized and completed (or discontinued) double-blind treatment, safety, tolerability, and any available PK data will be reviewed in a blinded fashion. Randomization of subjects into Group 2 (500 mg or placebo) will begin only after the 28-day, double-blind safety, tolerability, and available PK data for Group 1 are deemed acceptable by the Medical Monitor and the Sponsor. Blinded safety, tolerability, and PK data for the 500 mg cohort will be reviewed on an ongoing basis

No major concerns were identified in the previous study (EVP-0334-001) of single ascending doses (10 to 400 mg) and multiple ascending doses (10 to 400 mg for 14 days) that would preclude the initial clinical evaluation of orally administered doses of FRM-0334 (300 and 500 mg) for up to 28 days. These doses were based on preclinical study results and results of one Phase 1 study in 87 healthy female and male subjects aged ≥18 to ≤65 years. Additional information is provided in the Investigator's Brochure.

A dosing duration of 28 days is considered adequate to assess the safety and tolerability of FRM-0334 and FRM-0334-induced changes on the PD biomarkers. The plasma PK sample collection schedule is considered appropriate to describe the PK profile of FRM-0334 given the present understanding of the PK profile of this compound.

Placebo is included as a comparator. Bias arising from the assignment of subjects to a treatment group as well as from the expectations of subjects, investigators, and individuals collecting data will be minimized by randomization, double-blinding, and the use of a placebo control group. A screening period is included to ensure that laboratory test results (related to inclusion/exclusion criteria) and other safety data are available for medical review

before subject randomization. These design characteristics are accepted methods of controlling bias and providing a basis for statistical inference in clinical trials.

In addition to plasma biomarkers, CSF biomarkers will be evaluated. The CSF has major advantages in the study of neurologic conditions, although sampling CSF is more invasive than sampling blood or urine. Because of its close proximity to the CNS, the CSF may more accurately reflect ongoing pathology/biology of the brain, spinal cord, and meninges, and therefore may provide important and novel information.

In addition to standard safety assessments, the Columbia-Suicide Severity Rating Scale (C-SSRS) is included as a standard, required instrument to assess suicidality. The electrocardiogram (ECG) recordings will be reviewed centrally to establish continuity and uniformity of cardiac assessment.

The safety monitoring practices employed in this protocol are adequate to protect the subjects' safety.

Overall, this study design will address key clinical and scientific areas of interest in treating subjects with FTD-GRN and will attempt to gain further understanding of the effects of FRM-0334 in this population. Results of the current study will be used to identify the most appropriate doses and endpoints to use in future studies in FTD-GRN.

2. OBJECTIVES

2.1 Primary Objectives

- Evaluate the safety and tolerability of 2 fixed doses of FRM-0334 (300 and 500 mg daily in 2 sequential periods) over 28 days in subjects with prodromal to moderate FTD-GRN
- Assess the PD effects of FRM-0334 on the change from baseline in plasma concentrations of PGRN after 28 days

2.2 Secondary Objectives

- Assess the PD effects of FRM-0334 on the change from baseline in CSF concentrations of PGRN after 28 days
- Characterize the plasma and CSF concentrations of FRM-0334 and metabolites following once daily dosing after 28 days
- Characterize the intra- and inter-individual variability in plasma and CSF concentrations of PGRN

2.3 Exploratory Objectives

- Assess the effects of FRM-0334 after 28 days on the change from baseline in function using the Frontotemporal Dementia Clinical Dementia Rating Sum of Boxes (FTD-CDR-SB) scale and the Alzheimer's Disease Cooperative Study – Clinical Global Impression of Change (ADCS-CGIC) scale, and activities of daily living using the Frontotemporal Dementia Rating Scale (FRS) in subjects requiring a support person

- Assess the PD effects of FRM-0334 after 28 days on the change from baseline in plasma concentrations of PGRN mRNA and the CSF concentrations of total tau (t-tau), tau phosphorylated at threonine 181 (p-tau^{181}), 42 amino acid form of β-amyloid (Aβ42), neurofilament light chain (NF-L) and additional CSF biomarkers (eg, TDP-43), as technically feasible, and regional cerebral glucose metabolism using 18F-FDG-PET

3. OVERVIEW OF STUDY DESIGN

3.1 Overview

This is a randomized, double-blind, placebo-controlled, dose-escalating, Phase 2a study to evaluate the safety, tolerability, PD, and PK of FRM-0334 for 28 days in subjects with prodromal to moderate FTD-GRN. For this study, the condition of "prodromal" does not require any clinically visible or measurable symptoms, but only the presence of the genetic mutation FTD-GRN. Subjects will receive FRM-0334 or placebo during 2 sequential periods (Group 1: 300 mg or placebo; Group 2: 500 mg or placebo [identical number of subjects per period]). After all subjects in Group 1 (300 mg [n=12] or placebo [n=3]) have been randomized and completed (or discontinued) double-blind treatment, safety, tolerability, and any available PK data will be reviewed in a blinded fashion. Randomization of subjects into Group 2 (500 mg [n=12] or placebo [n=3]) will begin only after the 28-day, double-blind safety, tolerability, and available PK data for Group 1 (300 mg or placebo) are deemed acceptable by the Medical Monitor and the Sponsor. Blinded safety, tolerability, and PK data for the 500 mg cohort will be reviewed on an ongoing basis. Enrollment will be competitive. Recruitment will be terminated after randomization of 30 subjects.

Approximately 30 subjects (24 to FRM-0334 and 6 to placebo) will be randomized during 2 sequential periods; 15 subjects will be randomized into each period including 12 subjects to receive FRM-0334 and 3 subjects to receive placebo.

The eligibility of subjects will be determined during screening and confirmed on Day -7 and predose on Day 1 before the first dose of study drug is administered in the study center. In general, male and female subjects, ≥21 and ≤75 years of age, with prodromal to moderate FTD-GRN are eligible. Both symptomatic and non-symptomatic subjects are eligible. Each subject will be assessed, and those who are cognitively and functionally self-sufficient (do not require support) in the judgment of the investigator at screening are not required to have a support person. Subjects required to have a support person must have a Clinical Dementia Rating Scale Sum of Boxes (CDR-SB) score <16 at screening.

The support person will accompany the subject to study visits on Days 1 and 28 or be available by telephone, and if not living in the same household, interacts with the subject approximately 4 times per week. Additional eligibility (inclusion and exclusion) criteria are listed in Sections 4.2 and 4.3.

Only site personnel (rater) who have received training and certification, if applicable, in the administration and scoring of the rating scales will administer these scales. Whenever possible, for consistency of ratings, each rating scale should be administered by the same rater throughout the study. Only subjects with a support person will have the ADCS-CGIC, FTD-CDR-SB, and FRS scales completed; these scales require support person input.

Subjects will be instructed to ingest study drug (3 capsules daily for Group 1 and 5 capsules daily for Group 2) once daily, in the morning at approximately the same time each day, with food and water. On Day 1, subjects will ingest the first dose of study drug with food and water in the presence of study center personnel. All additional doses will be ingested as outpatients, with the exception of study visits on Days 7, 14, and 28. On these days, subjects will return all remaining study drug supplies to study center personnel; subjects will ingest study drug with food and water in the presence of study center personnel, after the predose blood sample collection for the PK and PD analysis and laboratory tests, including the pregnancy test for women of childbearing potential, has been obtained.

Screening Period (Days -30 to -8): Subjects (or legally acceptable representative) and support person (if applicable) will provide informed consent and acknowledgement of responsibilities, respectively, before any study-specific procedures are performed. Subjects will be screened for eligibility, as shown in Table 1. Each subject will be assessed, and those who are cognitively and functionally self-sufficient in the judgment of the investigator at screening are not required to have a support person. Subjects, who are required to have a support person in the judgment of the investigator, will have the CDR-SB performed at screening (score <16 is required for study entry). The assessments and procedures to be completed during the screening period are described in Section 9.1.1 and listed in Table 1.

Day -7 Assessments for the PD Biomarkers and Randomization: In the morning of Day -7 (or within approximately 7 days prior to the first dose of study drug on Day 1), eligible subjects will have procedures performed for the plasma and CSF PD biomarkers, CSF PK for FRM-0334 and metabolites, and the 18F-FDG-PET scan. Subjects will undergo lumbar puncture for the CSF sample collection, and they will be required to remain supine for approximately 2 hours afterwards, or as dictated by local practice. A CSF sample will also be sent for safety laboratory testing. Anxious and/or agitated subjects may receive a sedative in order to undergo the procedure if needed in the judgment of the investigator. This visit may be extended for up to 3 days, after consultation with the Medical Monitor, in order to schedule the CSF sample collection or the 18F-FDG-PET scan.

Subjects, who do not qualify for the study based upon the screening criteria and the investigator's assessment regarding subjects' ability to complete all study procedures,

including 18F-FDG-PET and lumbar puncture for the CSF sample collection, will be discontinued.

The assessments and procedures to be completed on Day -7 for the PD biomarkers are discussed in Section 9.1.2 and listed in Table 1.

Predose on Day 1 (Baseline), Double-Blind Treatment (Days 1 to 28) and Study Center Discharge on Day 29: In the morning of Day 1 (predose), baseline assessments will be performed to confirm eligibility for continued study participation and study drug dosing, as shown in Table 1. Eligible subjects will be randomized in the Interactive Voice/Web Response System (IXRS) to receive FRM-0334 or placebo during 2 sequential periods (Group 1: 300 mg or placebo; Group 2: 500 mg or placebo). A 1-week supply of study drug will be dispensed on Days 1 and 7, and a 2-week supply will be dispensed on Day 14; subjects will be instructed to ingest study drug once daily, in the morning at approximately the same time each day, with food and water.

On Day 1, subjects will ingest the first dose of study drug with food and water in the presence of study center personnel after the predose blood sample collection for the PK and PD analysis and laboratory tests, including the urine pregnancy test for women of childbearing potential, have been obtained. All additional doses will be ingested as outpatients, with the exception of study visits on Days 7, 14, and 28, at which study drug will be ingested in the presence of study center personnel. On these days, subjects will return all remaining study drug supplies to study center personnel.

On Day 21 (±2 days), subjects will be contacted by telephone for a safety evaluation (adverse events [AEs], concomitant medications, and suicidality using the C-SSRS). On Day 28, subjects will return for the final double-blind visit. Last dose of study drug will be ingested in the clinic on Day 28; maximum duration of study drug dosing for any subject is 28 days. If needed, subjects may have the 18F-FDG-PET scan only performed on Day 27 ideally in the afternoon, but the scan should not be performed on Day 29.

On Day 1, subjects will be required to remain in the study center for approximately 8-10 hours postdose or until all study-specific procedures have been completed. On Day 28, subjects will return to the study center. Assessments will be performed through Day 29, after which subjects will be discharged. Subjects should provide all applicable concomitant medications to study center personnel on Days 1, 7, 14 and 28, and study center personnel may administer these medications to subjects at the appropriate times.

The assessments and procedures to be completed during the double-blind period are discussed in Section 9.1.3 and listed in Table 1.

Early Termination (ET) Visit: Subjects who prematurely discontinue double-bind treatment will be encouraged to return to the clinic for an ET visit. Assessments and procedures to be completed are discussed in Section 9.1.3.5 and listed in Table 1.

Safety Follow-up : Subjects, who complete double-blind treatment will return to the study center for a safety follow-up on Day 38 (+2 days). Assessments and procedures to be completed are discussed in Section 9.1.3.6 and listed in Table 1. Additional safety assessments (eg, ECG, clinical laboratory tests) may be performed as requested by the investigator.

SAE Follow-up: Subjects, including those who prematurely discontinue, will be contacted by telephone to assess any serious adverse event (SAE) until 30 days (+2 days) after the last dose of double-blind study drug.

Any treatment-emergent clinically significant abnormalities persisting at the end of the study or ET will be followed until resolution or until reaching a clinically stable endpoint.

Unscheduled Visits: Unscheduled visits are allowed, and assessments will be performed as needed and at the discretion of the investigator. Any treatment-emergent clinically significant abnormalities persisting at the end of the study or ET will be followed until resolution or until reaching a clinically stable endpoint.

4. STUDY POPULATION

4.1 General Considerations and Number of Subjects

The study population will include male and female subjects, aged ≥21 to ≤75 years, with prodromal to moderate FTD-GRN. For this study, the condition of “prodromal” does not require any clinically visible or measurable symptom, but only the presence of the genetic mutation FTD-GRN. Subjects who require a support person, as determined by the investigator, must have a CDR-SB score <16 at screening. Subjects with severe disease or a score ≥16 will be excluded. Subjects must meet all of the inclusion and exclusion criteria for eligibility, as included in Sections 4.2 and 4.3. Additionally, in the judgment of the investigator, subjects who cease to meet any inclusion criterion, or meet one or more exclusion criterion during the study, may be ineligible to continue participating in the study.

4.1.1 Support Person Responsibilities

In the judgment of the investigator, or as required by local regulations, subjects may be required to have a support person who is willing to participate in the study. The support person will accompany the subject to study visits at screening (first study-specific activity for the support person) and on Days 1 and 28 (or be available for a telephone interview on Days 1 and 28).

The support person agrees to the minimum requirements for participation, as follows:

- Willing to interact with the subject at least 4 times a week
- Willing to sign an acknowledgment of responsibilities form in the study center before any study-specific procedures for the support person begin indicating that he/she understands the purpose of and the procedures required, and agrees to comply with these procedures (first study-specific procedure will be performed at screening)

The support person should remain consistent throughout the study, whenever possible.

4.2 Inclusion Criteria

Subjects must meet all of the following inclusion criteria for eligibility to participate in the study:

1. Informed consent form (ICF) signed by the subject or legally acceptable representative indicating that the subject or legally acceptable representative understands the purpose of and procedures required for the study before any study-specific procedures are performed
2. A support person may be required to participate in the study (in the judgment of the investigator at screening or as required by local regulations). The support person must sign an acknowledgement of responsibilities form at the study center before any study-specific activities required for the support person are performed. The first required activity will be performed at screening. The support person will accompany the subject to the study center at screening and on Days 1 and 28 (or be available by telephone on Days 1 and 28), and if not living in the same household, interacts with the subject approximately 4 times per week, and be able to complete the study
3. Male or female subjects aged ≥21 and ≤75 years
4. Genotyped positive for a FTD-GRN mutation
5. Prodromal to moderate FTD-GRN, and for subjects who require a support person (refer to inclusion criterion No. 2), a Clinical Dementia Rating Sum of the Boxes (CDR-SB) score <16 at screening
6. Fertile, sexually active subjects (men and women) must practice true abstinence or use an effective method of contraception during the study. Female subjects and the female partner of male subjects must be surgically sterile (hysterectomy or bilateral salpingectomy/oophorectomy or bilateral tubal occlusion/ligation), postmenopausal for at least 1 year prior to screening, or willing to consistently and correctly practice adequate methods of contraception if of childbearing potential (defined as consistent use of combined effective methods of contraception [including at least 1 barrier method])
7. Women of childbearing potential must have a negative pregnancy test at screening and Day 1
8. Resides in a stable living situation, living at home, senior residential setting, or an institutional setting without the need for continuous (ie, 24-hour) nursing care

9. Proficiency (oral and written) in the language in which study-related documents, including the ICF and standardized tests, will be administered
10. Able to swallow capsules
11. Be in good general health, willing and able to comply with the protocol requirements, and expected to complete the study as designed (in the judgment of the investigator)

4.3 Exclusion Criteria

Subjects who meet any of the following exclusion criteria are ineligible to participate:

Exclusion Criteria – Medical

1. Employees of the investigator or study center or their family members, or employees of FORUM Pharmaceuticals or Worldwide Clinical Trials who are directly involved in the conduct of the study
2. Female subjects who are pregnant, breastfeeding, or planning to become pregnant during the study
3. Unstable medical condition that is clinically significant (in the judgment of the investigator) within 30 days before screening
4. Untreated vitamin B_{12} or folate deficiency (must be stably treated for at least 6 months before screening)
5. Clinically significant untreated hypothyroidism (if treated, thyroid-stimulating hormone level and thyroid supplementation dose must be stable for at least 6 months before screening)
6. Clinically significant abnormal serum electrolytes (sodium, potassium, and magnesium) after repeat testing (in the judgment of the investigator)
7. Alanine transaminase (ALT) or aspartate transaminase (AST) >2.5 times the upper limit of normal
8. Renal insufficiency with serum creatinine >2.0 mg/dL, unless receiving current treatment with an angiotensin-converting enzyme (ACE) inhibitor in which case the Medical Monitor should be contacted
9. Insufficiently controlled diabetes mellitus (in the judgment of the investigator)
10. Clinically significant hematologic abnormalities including thrombocytopenia and leukocytosis (in the judgment of the investigator)
11. Malignant tumor within 3 years before screening with the exception of squamous and basal cell carcinoma or cervical carcinoma in situ or brachytherapy for localized prostate cancer

12. Systemic infection of any kind or any acute, subacute or chronic inflammatory process (eg, rheumatoid arthritis, chronic obstructive pulmonary disease, or gastrointestinal inflammatory diseases)

Exclusion Criteria – Neurological

13. Magnetic resonance imaging (MRI) or computed tomography (CT) scan performed within 12 months before screening with findings consistent with a clinically significant comorbid pathology other than FTD. If the MRI or CT scan is unavailable or occurred >12 months before screening, an MRI scan must be completed and findings confirmed before the Day -7 procedures are performed and a copy of the digital imaging and communications in medicine (DICOM) standard image and report must be available
14. Diagnosis of motor neuron disease, including probable amyotrophic lateral sclerosis
15. History of brain tumor, subdural hematoma, or other clinically significant (in the judgment of the investigator) space-occupying lesion on CT or MRI
16. Stroke within 18 months before screening or history of a stroke concomitant with onset of dementia
17. Head trauma with clinically significant (in the judgment of the investigator) loss of consciousness within 12 months before screening or concurrent with the onset of dementia
18. Onset of dementia within 12 months before screening secondary (in the judgment of the investigator) to cardiac arrest, surgery with general anesthesia, or resuscitation
19. Specific degenerative CNS disease diagnosis other than FTD (eg, Parkinson's disease, Alzheimer's disease, Huntington's disease, Creutzfeldt-Jakob disease, Down's syndrome)
20. Wernicke's encephalopathy
21. Epilepsy if present antiseizure therapy is required for seizure control

Exclusion Criteria – Psychiatric

22. Current diagnosis of severe major depressive disorder with psychotic features, if the present condition or treatment interferes with the subject's ability to complete the study (in the judgment of the investigator)
23. Significant suicide risk as defined by 1) suicidal ideations as endorsed on items 4 or 5 on the C-SSRS within the past year at screening or baseline, 2) suicidal behaviors detected by the C-SSRS within 2 years before screening, or 3) investigator assessment
24. History or current diagnosis of psychosis
25. History within 2 years before screening or current evidence of substance abuse as defined by the Diagnostic and Statistical Manual of Mental Disorders 4th Edition Text Revision

26. Clinically significant urine drug screen (UDS) or serum alcohol test result (in the judgment of the investigator) at screening (may be repeated once)

Exclusion Criteria – Cardiovascular

27. Clinically significant abnormality on screening or baseline ECG, including but not necessarily limited to a confirmed corrected QT interval corrected for heart rate (QTc) value ≥450 msec for males or ≥470 msec for females. In subjects with a QRS value >120 msec those with a QTc value >480 msec are NOT eligible
28. History of myocardial infarction or unstable angina within 6 months before screening or history of >1 myocardial infarction within 5 years before screening
29. Clinically significant (in the judgment of the investigator) cardiac arrhythmia (including atrial fibrillation), cardiomyopathy, or cardiac conduction defect (subjects with a pacemaker are acceptable)
30. Cardiovascular disease history including symptomatic hypotension or hypertension (supine diastolic blood pressure >95 mmHg) not stabilized by medical therapy (in the judgment of the investigator)

Exclusion Criteria – Prohibited Prior and Concomitant Medications

31. Investigational drug, biologic or medical device within 30 days before screening or planning to use an investigational drug (other than FRM-0334) during the study
32. Sensitive and/or narrow therapeutic range CYP1A2 substrates including alosetron, duloxetine, melatonin, ramelteon, theophylline and tizanidine
33. Warfarin-derived anticoagulants, heparin, NSAIDs, and anti-platelet drugs (clopidogrel and others) 10 days before the CSF sample collection on Day -7 and Day 28
34. Hypersensitivity to HDACi medication or currently receiving treatment with an HDACi, including valproic acid and amisulpride
35. Immunosuppressants including systemic corticosteroids and nonsteroidal anti-inflammatory drugs (NSAIDs) (Note: occasional use of NSAIDs is allowed, with the exception of 10 days before the CSF sample collection on Day -7 and Day 28)
36. Chronic intake of opioid containing analgesics
37. Psychostimulants, including amphetamines
38. Clozapine and amisulpride; other antipsychotics are allowed if on a stable dose (no change in the dose or frequency) for at least 30 days before screening and expected to remain stable during the study

5. RANDOMIZATION AND BLINDING

5.1 Randomization

Central randomization will be used to avoid bias in the assignment of subjects to double-blind treatment (FRM-0334 or placebo) and to increase the likelihood that known and unknown subject characteristics will be evenly distributed across the treatment groups. Eligible subjects will be randomly assigned to FRM-0334 or placebo during 2 sequential periods (Group 1: 300 mg or placebo; Group 2: 500 mg or placebo [identical number of subjects per period]).

After Day 1 predose procedures are completed, subjects who qualify will be assigned a randomization number and treatment group. A randomization schedule will be used to program the IXRS. After a subject is randomized in the IXRS; the next sequentially available randomization number will be assigned to that subject. The IXRS will also assign the appropriate bottle(s) of double-blind study drug, which will be dispensed during double-blind treatment on Day 1 after the predose procedures are completed and on Days 7 and 14 (but not on Day 28).

An assigned randomization number cannot be re-assigned even if the subject prematurely discontinues the study or withdraws prior to receiving any double-blind study drug. Subjects previously randomized will not be re-screened.

5.2 Blinding

During treatment, subjects, support persons, and family members, as well as, the investigators and other study center personnel and clinical staff will remain blinded to the treatment assignment (Group 1: 300 mg or placebo; Group 2: 500 mg or placebo). The Medical Monitor, study center monitors, and other Sponsor representatives involved in the clinical aspects of the study also will remain blinded to the double-blind treatment assignment.

All study drug capsules (FRM-0334 100 mg and placebo) will be identical in appearance (size- and color-matched). Furthermore, study drug capsules will be packaged in bottles that are indistinguishable from one another, except for a unique study bottle number that will be included on the label affixed to each bottle.

5.3 Premature Unblinding

Under normal circumstances, the identity of double-blind study drug will remain unknown, until all subjects have completed the study and the database is final and locked. If the treatment assignment is essential for emergent medical management of the subject, the investigator will contact the Medical Monitor to discuss the rationale for unblinding, unless any potential delay in providing emergency treatment places the subject at greater risk.

If the investigator is unable to contact the Medical Monitor, the investigator may, in an emergency, contact the IXRS to determine the identity of the treatment. If unblinding occurs without prior approval, the investigator should promptly communicate the circumstances leading to the unblinding by telephone and in writing to the Medical Monitor.

Any subject whose study drug treatment is unblinded will be discontinued and documented on the appropriate page (study completion information) of the electronic case report form (eCRF).

Please note that blinded safety and PK data for Group 1 (300 mg [n=12] or placebo [n=3]) will be reviewed during the study. After all subjects in Group 1 have completed (or discontinued) double-blind treatment, safety, tolerability, and any available PK data will be reviewed in a blinded fashion. Randomization of subjects into Group 2 will begin only after the 28-day, double-blind safety, tolerability, and available PK data for Group 1 are deemed acceptable by the Medical Monitor and the Sponsor. Blinded safety, tolerability, and PK data for the 500 mg cohort will be reviewed on an ongoing basis.

6. DOSE AND STUDY DRUG ADMINISTRATION

On Day 1 (predose), eligible subjects will be randomly assigned to a treatment group during 2 sequential periods (Group 1: 300 mg or placebo; Group 2: 500 mg or placebo [identical number of subjects per period]) in the IXRS. Subjects will ingest an oral dose of study drug once daily, in the morning at approximately the same time each day, with food and water for 28 days of double-blind treatment. The maximum duration of study drug exposure for any subject is 28 days.

On Day 1, subjects will ingest the first dose of study drug with food and water in the study center in the presence of study center personnel. All additional doses will be ingested as outpatients, with the exception of study visits on Days 7, 14, and 28. On these days, subjects will return all remaining study drug supplies to study center personnel. Subjects will ingest study drug with food and water in the presence of study center personnel, after the predose blood sample collection for the PK and PD analysis and laboratory tests, including a urine pregnancy test for women of childbearing potential has been obtained. When study drug is ingested in the presence of study center personnel, a hand and mouth check will be performed.

Subjects will be instructed to ingest study drug once daily in the morning, at approximately the same time each day, with food and water.

7. STUDY DRUG COMPLIANCE

The investigator or designee will maintain a log of study drug capsules dispensed and returned. Study drug supplies for each subject will be inventoried and accounted for throughout the study.

Subjects will be asked to return all remaining study drug supplies to study center personnel at double-blind visits on Days 7, 14, and 28 or ET and inform study staff of any missed doses. Percentage compliance will be calculated by dividing the number of doses (capsules) taken by the number of days between visits and by subject interview. Subjects in Group 1 are to ingest 3 capsules of study drug daily, and those in Group 2 are to ingest 5 capsules of study drug daily.

If a subject fails to return unused study drug, this subject will be asked to do so at the next visit. Study staff will remind the subject of the importance of taking study drug as directed and all study drug supplies at each visit. Final collection will occur no later than the final double-blind visit (Day 28) or ET.

8. PRIOR AND CONCOMITANT MEDICATIONS

All prescription and over-the-counter medications, including dietary and herbal supplements, taken by subjects within 30 days before screening and during the study (starting with the signing of the ICF) will be recorded on the eCRF with the indication, total daily dose, and dates of drug administration. Any additions, deletions, or changes in the dose of concomitant medications will be recorded. Subjects will be reminded to inform the investigator before taking any new medications.

8.1 Allowable Medications

Allowable medications include those used to control acceptable chronic medical conditions, if the doses and conditions being treated are stable (no change in the dose or frequency of dosing) for at least 30 days before screening and are expected to remain stable during the study. All coexistent diseases or conditions will be treated in accordance with prevailing medical practice.

Clozapine and amisulpride should not be administered during the study. Other antipsychotic medications are allowed if the subject has been on a stable dose (no change in the dose or frequency) for at least 30 days before screening and expected to remain stable during the study. However, antipsychotic medications, anxiolytics, and sedative-hypnotic medications are not permitted within 8 hours prior to the administration of the clinical rating scales on Days 1 and 28.

Anxiolytics or sedative-hypnotic medications are allowed, if needed, in the judgment of the investigator (but not within 8 hours before the clinical rating scales, if applicable, on Days 1 and 28).

Any medication not specifically prohibited in the protocol is allowed during the study provided the dose of the drug and the condition being treated have been stable for at least 30 days before screening, and the condition and the medication are expected to remain stable during the study.

8.2 Prohibited Medications

The exclusion criteria for prohibited prior and concomitant medications include a list of medications prohibited during the study (Section 4.3). In general, these include medication classes or medications considered likely to impact the evaluation of the PD biomarkers, PK parameters, or complicate the assessment of safety.

Medications prohibited during this study include the following (additional guidance provided in Section 4.3):

- The following CYP1A2 substrate medications: alosetron, duloxetine, melatonin, ramelteon, theophylline and tizanidine
- Warfarin-derived anticoagulants, heparin, NSAIDs, and anti-platelet drugs (clopidogrel and others) are prohibited 10 days before the CSF sample collection on Day -7 (from Days -17 to -7), as well as 10 days before the CSF sample collection on Day 28 (from Days 18 to 28)
- Immunosuppressants including systemic corticosteroids and NSAIDs (occasional NSAIDs are approved, but prohibited 10 days before the CSF collection on Day -7 (from Days -17 to -7), as well as 10 days before the CSF sample collection on Day 28 (from Days 18 to 28)

The Medical Monitor must be notified, in advance, if any prohibited medication is to be administered; if advanced notification does not occur, the Medical Monitor should be contacted as soon as possible thereafter and the management of the medication and continuation of the subject will be discussed.

Unless otherwise specified, a minimum washout period of 14 days or 5 half-lives, whichever is longer or as noted in the eligibility criteria, is required for all subjects receiving prohibited medications (prescription and over-the-counter) and must be completed before study drug dosing on Day 1.

9. STUDY ASSESSMENTS

The schedule of events (Table 1) summarizes the frequency and timing of assessments.

9.1 Overview of Study Assessments by Visit

9.1.1 Study visits should occur on the designated days, if possible. Screening Period (Days -30 to -8)

Screening procedures include the following:

- Signed ICF from the subject before any study-specific procedures for the subject are performed
- Signed acknowledgment of responsibilities from the support person (if applicable) at the study center before any study-specific activities required for the support person are performed (first study-specific support person activity will be performed at screening)
- Medical history review with the subject and, if possible, obtain the subject's current medical records
- Demographic information collection
- Inclusion and exclusion criteria review
- Prior (previous) and concomitant (current) medications review
- Complete physical examination, including height and a brief neurological examination
- Body weight and vital signs measurements, including orthostatic measurements of pulse and diastolic and systolic blood pressure obtained supine (after 5 minutes) and upon standing (after 2 minutes), and supine (after 5 minutes) respiratory rate and body temperature
- 12-lead ECG in triplicate (after 5 minutes supine)
- Urine pregnancy test (women of childbearing potential), using a local urine dipstick test. If positive, a serum test will be performed by the central laboratory, and the result must be negative before a subject may be randomized to treatment
- Clinical laboratory tests: Collection of blood samples for hematology and serum chemistry (including thyroid stimulating hormone [TSH], vitamin B_{12}, and folate), and urine sample for urinalysis, using local urine dipstick test; if the urine test is positive and judged clinically significant by the investigator, a urine sample will be submitted to the central laboratory for microscopic analysis
- Drug screen (central laboratory): collect a urine sample for the UDS and a blood sample for the alcohol test (if positive, may be repeated once)
- Blood sample for PD biomarkers, PGRN, and PGRN mRNA
- CDR-SB (if applicable)
- C-SSRS
- MRI or CT within 12 months of screening to confirm pathology; findings must be confirmed before Day -7 procedures. For anatomical mapping of the 18F-FDG-PET imaging analysis, each subject must have an MRI scan with DICOM image available. The MRI scan with DICOM file must be available within 12 months of screening or by the end of the Day 14 (±2 days) visit (Section 9.2.4).

- AEs (collected from signing of the ICF through the follow-up visit or ET)

9.1.2 Screening Period (Day -7 [+3 days]* Visit for Pharmacodynamic Biomarkers)

- Inclusion and exclusion criteria review
- Concomitant medications recorded
- Vital signs including orthostatic measurements of pulse and diastolic and systolic blood pressure obtained supine (after 5 minutes) and upon standing (after 2 minutes) and supine (after 5 minutes) respiratory rate and body temperature
- AEs assessed and recorded
- Blood sample for the PD biomarkers, PGRN, and PGRN mRNA
- CSF sample for the PD biomarkers, including PGRN, t-tau, p-tau^{181}, Aβ42, NF-L, and additional biomarkers, including TDP-43, as technically feasible, concentration of FRM-0334 and metabolites, and safety laboratory testing; subjects will be required to remain supine for approximately 2 hours after the lumbar puncture, or as dictated by local practice

- 18F-FDG-PET scan

** This visit may be extended for up to 3 days, after consultation with the Medical Monitor, in order to schedule the CSF sample collection or the 18F-FDG-PET scan.*

9.1.3 Double-Blind Treatment (Days 1-28), Study Center Discharge (Day 29), Safety Follow-up (Day 38), and Serious Adverse Event Follow-up (Day 58)

9.1.3.1 Double-Blind Study Visit on Day 1

Adverse events will be assessed and recorded, and concomitant medications will be recorded.

In the morning of Day 1 (predose), baseline assessments will be completed to confirm eligibility for continued study participation as described below.

Predose Day 1 (Baseline) Procedures and Assessments

- Inclusion and exclusion criteria review
- Body weight and vital signs, including orthostatic measurements of pulse and diastolic and systolic blood pressure obtained supine (after 5 minutes) and upon standing (after 2 minutes) and supine (after 5 minutes) respiratory rate and body temperature
- Directed physical examination
- 12-lead ECG in triplicate (after 5 minutes supine) and reviewed by a physician at the study center before study drug is dispensed
- Clinical laboratory tests: Collection of blood samples for hematology and serum chemistry and urine sample for urinalysis, using local urine dipstick test; if the urine test is positive and judged clinically significant by the investigator, a urine sample will be submitted to the central laboratory for microscopic analysis
- Urine pregnancy test (women of childbearing potential), using a local urine dipstick test.
- Blood sample for the PD biomarkers, PGRN, and PGRN mRNA, and PK for FRM-0334 and metabolites

- ADCS-CGIC (source workbook), FTD-CDR-SB, and FRS (if applicable)
- C-SSRS
- Blood sample for future research to develop a method to detect GRN mutations and potentially genotyping drug-metabolizing enzymes

Study Drug Dosing on Day 1

- Randomization to double-blind treatment and study drug assignment by the IXRS
- Study drug instructions and study drug dispensed
- Administration of the first dose of study drug in the presence of site personnel with food and water

Postdose Day 1

- Record time of administration and complete study drug accountability and record compliance
- Vital signs within 4 and 6 hours postdose, including orthostatic measurements of pulse and diastolic and systolic blood pressure obtained supine (after 5 minutes) and upon standing (after 2 minutes) and supine (after 5 minutes) respiratory rate and body temperature
- 12-lead ECG in triplicate (after 5 minutes supine) at 4 hours postdose
- Blood sample for the PD biomarkers, PGRN, and PGRN mRNA, and PK of FRM-0334 and metabolites at 1, 2, 4, 6, and 8 hours postdose; an optional 10 hour postdose PK and PD sample may be collected

9.1.3.2 Double-Blind Treatment Study Visits on Days 7 and 14 (±2 days)

- Clinical laboratory tests samples predose: Collection of blood samples for hematology and serum chemistry and urine sample for urinalysis, using local urine dipstick test; if the urine test is positive and judged clinically significant by the investigator, a urine sample will be submitted to the central laboratory for microscopic analysis
- Urine pregnancy test (women of childbearing potential) obtained predose, using a local urine dipstick test. If positive, a serum pregnancy test must be performed by the central laboratory, and the result must be negative in order for the subject to continue treatment
- Blood sample obtained predose for PK of FRM-0334 and metabolites and PD biomarkers, PGRN and PGRN mRNA
- In the presence of the site personnel, administer study drug (with food and water), record the time of study drug administration, and complete study drug accountability and record compliance (collect remaining study drug supplies)
- Directed physical examination
- Vital signs (predose and 4 and 6 hours postdose), including orthostatic measurements of pulse and diastolic and systolic blood pressure obtained supine (after 5 minutes) and upon standing (after 2 minutes) and supine (after 5 minutes) respiratory rate and body temperature
- 12-lead ECG in triplicate (after 5 minutes supine), obtained 4 hours postdose
- C-SSRS

- AEs observed, assessed, and recorded
- Concomitant medications recorded
- Study drug compliance assessed
- Study drug instructions and study drug dispensed

9.1.3.3 Telephone Contact Visit on Day 21 (±2 days)

Subjects will be contacted by telephone for a safety assessment on Day 21 (±2 days).

- AEs assessed and recorded
- Concomitant medications recorded
- C-SSRS

A visit to the study center is not required, but may be substituted for the telephone contact, if deemed necessary by the investigator.

9.1.3.4 Final Double-Blind Treatment Study Visit on Day 28 and Study Center Discharge on Day 29

Adverse events will be observed, assessed, and recorded, and concomitant medications will be recorded.

Predose Day 28 Procedures and Assessments

- Clinical laboratory tests: Collection of blood samples for hematology and serum chemistry and urine sample for urinalysis, using local urine dipstick test; if the urine test is positive and judged clinically significant by the investigator, a urine sample will be submitted to the central laboratory for microscopic analysis
- Urine pregnancy test (women of childbearing potential), using a local urine dipstick test. If positive, a serum pregnancy test must be performed by the central laboratory.
- Blood sample for PD biomarkers, PGRN and PGRN mRNA, and PK of FRM-0334 and metabolites
- Vital signs, including orthostatic measurements of pulse and diastolic and systolic blood pressure obtained supine (after 5 minutes) and upon standing (after 2 minutes) and supine (after 5 minutes) respiratory rate and body temperature

Study Drug Dosing on Day 28

- Administration of study drug in the presence of site personnel

Postdose Day 28 Procedures and Assessments

- Record the time of administration, and complete study drug accountability and record compliance
- Collect remaining study drug supplies
- ADCS-CGIC, FTD-CDR-SB, and FRS (if applicable)
- Blood sample for the PD biomarkers, PGRN and PGRN mRNA, and PK of FRM-0334 and metabolites at 1, 2, 4, 6, 8, 10, and 12 hours postdose: an optional 16 hour postdose PK and PD sample may be collected

- Vital signs, including orthostatic measurements of pulse and diastolic and systolic blood pressure obtained supine (after 5 minutes) and upon standing (after 2 minutes) and supine (after 5 minutes) respiratory rate and body temperature, at 4 and 6 hours postdose
- 12-lead ECG in triplicate at 4 hours postdose (after 5 minutes supine)
- Directed physical examination
- Body weight
- C-SSRS
- 18F-FDG-PET scan ideally in the afternoon, but before the CSF sample collection (or if needed, the scan only may be performed on Day 27 ideally in the afternoon, but not on Day 29)
- CSF sample collection at approximately 7-10 hours postdose for the PD biomarkers, including PGRN, t-tau, p-tau^{181}, Aβ42, NF-L, and additional biomarkers (eg, TDP-43) as technically feasible, concentration of FRM-0334 and metabolites, and safety laboratory testing; subjects will be required to remain supine for approximately 2 hours after the lumbar puncture, or as dictated by local practice

Subjects will remain in the study center or other location (per institutional practice) overnight. If the subject stays overnight at a location other than the study center, every effort must be made to collect all required data points.

Day 29 Procedures, Assessments, and Study Center Discharge

- Vital signs, including orthostatic measurements of pulse and diastolic and systolic blood pressure obtained supine (after 5 minutes) and upon standing (after 2 minutes) and supine (after 5 minutes) respiratory rate and body temperature
- Blood sample for the PD biomarkers, PGRN and PGRN mRNA, and PK of FRM-0334 and metabolites (24 hours post-Day 28 dose)
- AEs observed, assessed, and recorded
- Concomitant medications recorded
- Discharge from the study center after all Day 29 procedures are performed upon approval by the investigator and reminded to return to the study center for the Day 38 follow-up visit

9.1.3.5 Early Termination Visit

Subjects, who prematurely discontinue double-blind treatment, will be encouraged to return to the study center for an ET visit. If a subject does not return for a scheduled visit or is lost to follow-up, every effort should be made to contact the subject, as described in Section 11.2. The following assessments will be completed:

- Body weight and vital signs, including orthostatic measurements of pulse and diastolic and systolic blood pressure obtained supine (after 5 minutes) and upon standing (after 2 minutes) and supine (after 5 minutes) respiratory rate and body temperature
- Directed physical examination
- 12-lead ECG in triplicate

- Blood sample for the PD biomarkers, PGRN and PGRN mRNA
- Clinical laboratory tests: Collection of blood samples for hematology and serum chemistry and urine sample for urinalysis, using local urine dipstick test; if the urine test is positive and judged clinically significant by the investigator, a urine sample will be submitted to the central laboratory for microscopic analysis
- Urine pregnancy test (women of childbearing potential), using a local urine dipstick test. If positive, a serum pregnancy test must be performed by the central laboratory.
- C-SSRS
- AEs assessed and recorded
- Concomitant medications recorded
- Complete study drug accountability and record compliance
- Collect remaining study drug supplies

9.1.3.6 Safety Follow-Up Visits (Days 38 and 58)

Day 38 Safety Follow-up

Subjects who complete double-blind treatment (Day 28) will return to the study center for safety follow-up on Day 38 (+2 days), and the following assessments will be performed:

- Body weight and vital signs, including orthostatic measurements of pulse and diastolic and systolic blood pressure obtained supine (after 5 minutes) and upon standing (after 2 minutes) and supine (after 5 minutes) respiratory rate and body temperature
- C-SSRS
- Blood sample for the PD biomarkers, PGRN and PGRN mRNA
- Clinical laboratory tests: If clinically relevant changes from baseline are observed at Day 28, collection of blood samples for hematology and serum chemistry and urine sample for urinalysis, using local urine dipstick test; if the urine test is positive and judged clinically significant by the investigator, a urine sample will be submitted to the central laboratory for microscopic analysis
- AEs assessed and recorded
- Concomitant medications recorded

Day 58 Serious Adverse Event Follow-up

All subjects, including those who prematurely discontinue, will be contacted by telephone to assess any SAE until 30 days (+2 days) after the last dose of study drug.

9.1.3.7 Unscheduled Visits

Unscheduled visits are allowed, and assessments will be performed as needed and at the discretion of the investigator.

9.2 Pharmacodynamic Biomarkers

9.2.1 Plasma Pharmacodynamic Biomarkers and Collection

A blood sample for the PD biomarkers, PGRN and PGRN mRNA, will be collected at screening, Day -7, during double-blind treatment (Days 1, 7, 14, 28, and 29), or ET, and follow-up (Day 38). On Day 1, a PD sample will be collected predose and 1, 2, 4, 6, and 8 hours postdose; an optional 10 hour postdose PD sample may be collected. On Days 7 and 14, a predose and PD sample will be collected. On Day 28, a PD sample will be collected predose and 1, 2, 4, 6, 8, 10, 12 and 24 hours postdose (before discharge on Day 29); an optional 16 hour postdose PD sample may be collected.

At the specified time points, approximately 14 mL of venous blood will be collected from an antecubital vein. For the PD analysis, a 6 mL sample for PGRN will be placed into a tube containing dipotassium ethylenediaminetetraacetic acid (EDTA K_2) and an 8 mL sample for PGRN mRNA will be placed into PAXgene blood DNA tubes. Procedures for the collection, storage, and shipping of samples for bioanalysis are located in a study manual.

9.2.2 Cerebrospinal Fluid Pharmacodynamic Biomarkers and Collection

Subjects will undergo a lumbar puncture for the CSF collection on Day -7 and approximately 7 to 10 hours postdose on Day 28 for the PD biomarkers and the CSF concentration of FRM-0334 and metabolites. The PD biomarkers include PGRN, t-tau, p-tau^{181}, Aβ42, and NF-L. Since biomarker research is constantly evolving, the samples obtained in this study may be analyzed for different or additional markers (eg, TDP-43), as technically feasible, with the aim of ensuring state-of-the-art analysis that will reflect the latest advances in biomarker research. Cerebrospinal fluid PK collection and handing information is provided in Section 9.3.2.

The risks of a standard lumbar puncture, including discomfort or pain around the procedure, bleeding into the spinal cord, headache after the test, and infection, will be explained to each subject. The investigator should also confirm the subject has not received an anticoagulant medication within 10 days of the CSF sample collection (Section 8.2). If needed in the judgment of the investigator, anxious and/or agitated subjects may receive a sedative in order to undergo the procedure. Subjects will be required to remain supine for approximately 2 hours after the collection of the CSF sample, or as dictated by local practice.

The total CSF fluid collected at each timepoint will be limited to 16 mL (32 mL total for the study). The initial 2 mL will be discarded, and the second 2 mL sample collected will be sent to a central laboratory for safety testing (total protein, glucose, and cell count in 2 aliquots). The remainder of the sample will be pooled before being divided into aliquots and will be used for the PK and PD analyses, and the future analysis of additional biomarkers.

Two 0.5 to 1 mL aliquots from each CSF sample collection will be reserved for each of the following analysis: PGRN concentration determination, and t-tau, p-tau[181], Aβ42, and NF-L concentration determination. Unless prohibited by local regulations, subjects (or their legally acceptable representative) will be asked to indicate on the consent form whether they will allow an additional sample (6 mL) to be reserved for potential future biomarker analysis (divided into 1 mL aliquots). Handling and processing of CSF samples will be detailed in a study manual. Briefly, all CSF aliquots will be placed into collection tubes and stored at approximately –70°C until shipped for analysis, with the exception of the aliquots for safety testing that will be sent to a central laboratory.

9.2.3 18F-Fluorodeoxyglucose Positron Emission Tomography Scan

The effects of FRM-0334 on the normalization of cortical metabolic activity will be studied using 18F-FDG-PET performed on Day -7 and postdose on Day 28 ideally in the afternoon, but before the CSF sample collection (or if needed, the scan may be performed on Day 27 ideally in the afternoon, but not on Day 29).

The 18F-FDG-PET scan will be obtained according to acceptable procedure guidelines and local hospital standards and the 18F-FDG will be administered as appropriate for a radiopharmaceutical (Boellaard et al, 2010). The subject preparation will include the following: subjects will not be allowed to consume any food or sugar for at least 6 hours before injection of 18F-FDG and adequate prehydration should be ensured.

The 18F-FDG-PET scan will be uploaded to a secure, central server within approximately 24 hours after the scan. The uploaded 18F-FDG-PET scan cannot be copied and can only be viewed by the blinded, external, 18F-FDG-PET scan reviewers or Sponsor-designated representatives. The external reviewer will assess the standardization and quality of the scans and evaluate the findings.

9.2.4 Magnetic Resonance Imaging

Magnetic resonance imaging may be used to confirm pathology at screening. In addition, each subject must have an MRI scan with a DICOM image available for anatomical mapping of the 18F-FDG-PET imaging analysis. The MRI scan with DICOM file must be available within 12 months of screening or by the end of the Day 14 (±2 days) visit.

Dependent on the information available at screening, an MRI scan may need to be performed during the study, as defined below:

- If a subject has an MRI scan with a DICOM image available from within the last 12 months, no additional scans are necessary.

- If a subject has an MRI scan without a DICOM image available from within the last 12 months, the MRI scan may be used to confirm pathology; however, an MRI scan with DICOM image must be performed prior to or during the Day 14 (±2 days) visit.
- If a subject has a CT scan available from within the last 12 months, the CT scan may be used to confirm pathology; however, an MRI scan with DICOM image must be performed prior to or during the Day 14 (±2 days) visit.
- If a subject has neither an MRI nor CT scan available, an MRI scan must be performed to confirm pathology prior to the Day -7 procedures and a DICOM image must be obtained.

9.2.5 Genotyping

A single blood sample (8.5 mL) will be reserved at baseline (Day 1 predose) for future research to develop a method to detect GRN mutations. The blood sample may also be used for genotyping of drug-metabolizing enzymes.

Unless prohibited by local regulations subjects (or their legally acceptable representative) will be asked to indicate on the consent form whether they will allow this sample to be reserved for potential future research. Subjects may still participate in the clinical trial if they elect not to allow this blood sample to be reserved.

9.3 FRM-0334 Pharmacokinetics

9.3.1 Plasma Pharmacokinetic Sample Collection and Handling

Blood samples (approximately 6 mL) for the PK analysis of FRM-0334 and metabolites will be collected during double-blind treatment. On Day 1, a PK sample will be collected predose and 1, 2, 4, 6, and 8 hours postdose; an optional 10 hour postdose and PK sample may be collected. On Days 7 and 14, a predose PK sample will be collected to determine trough plasma concentrations. On Day 28, a PK sample will be collected predose and 1, 2, 4, 6, 8, 10, 12 and 24 hours postdose (before discharge on Day 29); an optional 16 hour postdose PK sample may be collected.

The date and time of the PK sample collection and the date and time of the last dose of study drug, as reported by the subject, prior to sample collection will be recorded. Subjects may have an indwelling cannula inserted for the collection of PK blood samples. In these cases, about 1 mL of fluid will be removed from the cannula and discarded before each procedure.

Handling of Biological Specimens: At the specified time points, approximately 6 mL of venous blood will be collected from an antecubital vein into EDTA K_2 containing tubes for determination of the plasma concentrations of FRM-0334 and metabolites.

Procedures for the collection, storage and shipping of the plasma samples for bioanalysis are located in a study manual provided to the sites.

Analytical Methods: Plasma samples will be analyzed by a central laboratory to determine concentrations of FRM-0334 and, if relevant, metabolites, using a validated method. The study randomization will be provided to the bioanalytical laboratory to enable assay of the plasma samples for subjects randomized to FRM-0334.

Plasma PK Parameters: The plasma PK parameters for FRM-0334 and metabolites will be determined following single- and multiple-dose administration as shown in Table 2 and Table 3, respectively. The PK parameters will be determined using noncompartmental methods using WinNonlin version 5.1 or later (or equivalent).

Table 2 Pharmacokinetic Parameters of FRM-0334 after Single Dose Administration

Parameter	Definition
C_{max}	Maximum observed concentration
t_{max}	Time corresponding to occurrence of C_{max}
$AUC_{(0-t)}$	AUC from time zero to the last quantifiable concentration

Table 3 Pharmacokinetic Parameters of FRM-0334 after Multiple Dose Administration

Parameter	Definition
C_{trough}	Concentration immediately prior to dosing
C_{max}	Maximum observed concentration at steady state
t_{max}	Time corresponding to occurrence of C_{max} at steady state
$t_{½}$	Apparent terminal elimination half-life at steady state
$\lambda_{z.}$	Terminal elimination rate constant at steady state
$AUC_{(0-\tau)}$	AUC over the dosing interval at steady state
CL/F	Apparent clearance following oral administration at steady state
C_{av}	Average concentration at steady state

9.3.2 Cerebrospinal Fluid Pharmacokinetic Sample Collection and Handling

Cerebrospinal fluid will be collected on Day -7 and Day 28 as previously described in Section 9.2. Procedures for the collection, storage and shipping of the CSF samples for bioanalysis are provided in a study manual provided to the sites. Briefly, all CSF aliquots will be placed into collection tubes and stored at approximately -70°C until shipped for analysis, with the exception of the aliquots for safety testing that will be sent to a central laboratory. Two 1 mL aliquots from each CSF sample collection will be reserved for FRM-0334 and metabolite concentration determination.

9.4 Clinical Rating Scales

In order to ensure the collection of high quality data, all study raters will be trained, if applicable, in the proper administration of the clinical rating and safety scales. Training and certification, if applicable, will be documented and updated as necessary. Raters must be

approved by the Sponsor or designee before administering the scales to subjects. Refer to the study manuals for further details on training methodology.

Alzheimer's Disease Cooperative Study – Clinical Global Impression of Change (ADCS-CGIC): The ADCS-CGIC (Schneider et al, 1997) is a global rating scale that is derived through a series of independent, comprehensive interviews with the subject and their support person. A trained, independent, rater who is not caring for or managing the subject will administer and score the ADCS-CGIC. The ADCS-CGIC rating assesses the subject across 4 domains, including general (relevant history, clinical observation), mental/cognitive state, behavior, and activities of daily living (including instrumental and social activities). This assessment will be performed ≥8 hours after dosing with an antipsychotic and/or anxiolytic or sedative, if applicable.

At Day 1 (predose), the rater will interview the subject (first) and the support person and will have access to other baseline information as needed in order to develop a comprehensive impression of the subject's cognitive abilities and deficits, behavior, and activities of daily living. At subsequent evaluations, the rater will not have access to any study information for the subject other than the baseline ADCS-CGIC source workbook.

On Day 28 postdose, the rater's assessment of the magnitude of overall change in the subject's condition relative to baseline will be based solely on an interview with the subject (first) and the support person. The rater will score the ADCS-CGIC using a 7-point scale from 1 (marked improvement) through 7 (marked worsening).

Clinical Dementia Rating Scale Sum of Boxes (CDR-SB): The 6-item standard CDR-SB scale (Hughes et al, 1982) was developed to provide a global clinical measure of disease in subjects with dementia (this scale is referred to as the CDR-SB throughout this protocol). The CDR-SB will be administered at screening to subjects who require a support person for study entry in order to identify and exclude subjects with severe disease (those with a CDR-SB score ≥16).

The CDR-SB score ranges from 0 to 18, with a higher score indicating more severe impairment. The guidelines for administering and scoring the CDR-SB are identical to the FTD-CDR-SB as described below.

Frontotemporal Dementia Clinical Dementia Rating Sum of Boxes (FTD-CDR-SB): The 6-item CDR-SB (described above) was modified by Knopman et al, 2008 specifically for use in subjects with FTLD to capture key characteristics of the disease that are not explicitly measured by the CDR-SB. This 8-item modified scale is referred to as the FTD-CDR-SB throughout this protocol. The FTD-CDR-SB scale will be administered during double-blind

treatment only to subjects requiring a support person in the judgment of the investigator at screening, or as required by local regulations.

The FTD-CDR-SB includes the following 8 items:

- Six items from the CDR-SB: cognitive and functional domain of memory, orientation, judgment and problem solving, involvement in community affairs (eg, job, shopping, business, and financial responsibilities), home and hobbies (eg, household chores and recreational interests), personal care (eg, dressing, personal hygiene, and toileting) (Morris, 1993)
- Two additional items specific to FTD: language and behavior, comportment and personality were added by Knopman et al, 2008

The rating for each domain is scored using a scale of 0 (none) to 3 (severe) based on the subject's function in relation to cognitive ability (not impairment due to other factors) and past performance (or baseline level of functioning) and is assigned after an interview with both the support person and subject. The overall rating for each domain ("boxes") is summed to provide a global clinical measure of the disease. The FTD-CDR-SB score ranges from 0 to 24, with a higher score indicating more severe impairment.

A trained rater will administer and score the FTD-CDR-SB (and the CDR-SB at screening), using information collected during separate semi-structured interviews with the support person (interviewed first) and the subject. If possible, the same rater should complete the FTD-CDR-SB at these visits and the CDR-SB at screening. On Days 1 and 28, the FTD-CDR-SB will be performed ≥8 hours after dosing with an antipsychotic and/or anxiolytic or sedative, if applicable.

Frontotemporal Dementia Rating Scale (FRS): The FRS is a severity staging tool for subjects with FTD based upon functional dependence and behavioral changes and can be used to assess change in behavior and loss of ability over time (Mioshi et al, 2010); the scale is completed by the rater after a brief interview with the support person.

The FRS is a 30-item questionnaire that evaluates the areas of behavior (7 items), outing and shopping (2 items), household chores and telephone (3 items), finances (4 items), medications (2 items), meal preparation and eating (8 items), and self-care and mobility (4 items). Using the FRS scoring guide, each item is scored in comparison to the subject's premorbid functioning, as all the time, sometimes, or never.

A trained rater will administer and score the FRS at predose on Day 1 and postdose on Day 28 using support person input, which is only for subjects required to have a support person for study entry in the judgment of the investigator, or as required by local regulations.

9.5 Safety Assessments

Abnormal vital signs measurements, clinical laboratory tests results, physical examination, and ECG findings assessed as clinically significant by the investigator may be repeated. Any abnormal physical examination findings, ECG parameters, vital signs values, or laboratory tests results that meet the definition of an AE (Section 10.1.1) will be recorded on the AE eCRF.

Additionally any treatment-emergent clinically significant abnormalities persisting at the end of the study or ET will be followed until resolution or until reaching a clinically stable endpoint.

9.5.1 Medical History and Demographics

A general medical history will be obtained at the screening visit. Investigator assessment of past medical history at screening should include information regarding any significant medical, surgical, and psychiatric and/or neurological conditions and treatments.

The date of the first diagnosis of FTD-GRN, treatments received, and other details about this condition will be recorded. Support of the diagnosis of FTD-GRN may be obtained from additional clinical sources (eg, medical records review, telephone contact with the treating clinician, or written confirmation from the treating clinic).

Demographic data will include date of birth, sex, ethnic categorization (Hispanic or Latino, or not Hispanic or Latino), and race (ie, white, black or African American, Asian, American Indian/Alaskan Native, Native Hawaiian/other Pacific Islander, or other).

9.5.2 Prior and Concomitant Medications

All medications taken within 30 days before screening and throughout the study will be recorded on the prior and concomitant medication page of the eCRF, and any changes will be recorded. Concomitant medication use will be assessed at each study visit and the telephone contact visit on Day 21.

9.5.3 Physical Examination

A complete physical examination, including a brief neurological examination and height, will be performed at screening. The physical examination will include (but is not limited to) an examination of general appearance, skin, head, eyes, ears, nose, throat, neck (including thyroid), lymph nodes, chest, heart, lungs, abdomen, and extremities.

A directed physical examination will be performed at predose on Day 1, and postdose on Days 7, 14, and 28 or ET, and will include a general inspection of the subject. A more detailed examination may occur at these times if indicated by results of other safety assessments, general inspection, or signs or symptoms reported by the subject.

9.5.4 Vital Signs

Vital signs measurements will be recorded at each study visit, with the exception of Day 21 and the SAE Follow-up. On Days 1 and 28, vital signs will be measured at predose, and 4 and 6 hours postdose, and additionally on Day 28 at 24 hours postdose (or before discharge on Day 29). On Days 7 and 14, vital signs will be measured predose and 4 and 6 hours postdose. Vital signs will include orthostatic measurements for pulse and blood pressure (diastolic and systolic) and will be obtained before any scheduled blood sample collection and after the subject has been supine for approximately 5 minutes and again upon standing (after 2 minutes), and respiratory rate and body temperature will be measured after the subject has been supine for approximately 5 minutes.

Body weight will be measured at screening, predose on Day 1, postdose on Day 28, on Day 38, or ET.

9.5.5 Electrocardiogram

Standard 12-lead ECG tracings will be conducted in triplicate (1 to 3 minutes apart) after the subject has rested supine for at least 5 minutes at screening, predose, and 4 hours postdose on Day 1, 4 hours postdose on Days 7 and 14, 4 hours postdose on Day 28, or at ET. The ECG tracings will be recorded using an appropriately maintained and calibrated 12-lead electrocardiograph machine.

The ECG will be transmitted and interpreted by a central ECG facility. A physician at the study center who is proficient in reading ECG recordings will review the predose Day 1 ECG and indicate if the subject is appropriate to proceed and receive the first dose of study drug in the clinic on that day. Additionally, a physician at the study center who is proficient in reading ECG recordings will review each ECG as a safety assessment.

9.5.6 Clinical Laboratory Tests

The clinical laboratory tests will be analyzed by a central laboratory that will provide laboratory kits to the study centers before study initiation and instructions will be provided in a study manual. Specimens will be appropriately processed by the central laboratory and laboratory reports will be made available to the investigator in a timely manner to ensure appropriate clinical review. The laboratory test battery will include both screening and routine laboratory tests. A CSF safety laboratory sample obtained on Day -7 and Day 28 will be sent to a central laboratory to assess total protein, glucose, and cell count (Section 9.2.2).

9.5.6.1 Screening Tests

Urine Drug Screen and Serum Alcohol Test: A UDS and serum alcohol test will be performed at screening and may be repeated once if positive. Positive test results are not necessarily exclusionary and may not require that a subject be withdrawn. However, if the

UDS and/or serum alcohol test results are judged clinically significant in the judgment of the investigator, the subject will be excluded from the study.

The UDS will test for the presence of amphetamines, barbiturates, benzodiazepines, cocaine, opiates, phencyclidine, cannabinoids, and tricyclic antidepressants.

Thyroid-Stimulating Hormone, Serum Vitamin B_{12}, and Serum Folate: Thyroid dysfunction will be detected by measuring TSH and a deficiency of vitamin B_{12} and folate will be detected by measuring serum vitamin B_{12} and folate at screening.

9.5.6.2 Routine Clinical Laboratory Tests

The routine clinical laboratory tests will be performed at screening and predose on Days 1, 7, 14, and 28 or ET. If clinically relevant changes from baseline are observed on Day 28, clinical laboratory tests will be performed on Day 38.

Hematology Panel: The hematology panel will include white blood cell count with differential (absolute and percentage values for lymphocytes, monocytes, neutrophils, eosinophils, and basophils), red blood cell count, hemoglobin, hematocrit, and platelet count. Prothrombin time will be assessed at screening. If a subject takes an anticoagulant at any point during the study, prothrombin time should also be assessed at Day 28 (prior to CSF collection).

Chemistry Panel: The chemistry panel will include serum glucose (random), sodium, potassium, chloride, bicarbonate, calcium, magnesium, blood urea nitrogen, creatinine, uric acid, inorganic phosphorus, total protein, albumin, alkaline phosphatase, AST, ALT, gamma-glutamyl transferase, total bilirubin, and creatine phosphokinase.

Urinalysis: Urinalysis, performed using dipstick urine test by study center personnel, will include ketones, protein, glucose, leukocytes, nitrites, hematuria, color, specific gravity, and pH. If results of the urinalysis dipstick evaluation are positive and judged clinically significant by the investigator, a urine sample will be submitted to the central laboratory for microscopic analysis.

Pregnancy Test (females of childbearing potential): A urine dipstick pregnancy test will be performed by study center personnel. If positive, a serum pregnancy test (except Day 1 predose) will be performed by the central laboratory; this can be the same sample submitted for the chemistry panel. The result must be negative in order for the subject to continue study participation. Any subject with a confirmed positive pregnancy test will be discontinued and reported to the Sponsor or designee as described in Section 10.7.

9.5.6.3 Total Blood and Cerebrospinal Fluid Volume

The number of blood samples and the estimated maximum total blood volume obtained from each subject during the study for the clinical laboratory tests, PK and PD analysis is approximately 458.5 mL (498.5 mL with optional PK/PD samples) as presented in Table 4.

The total CSF volume collected during the study for the PD biomarkers, concentration of FRM-0334 and metabolites, and safety laboratory tests is approximately 32 mL.

Table 4 Total Blood and CSF Volume Collected per Subject (FRM-0334-002)

Assessment	Number of Samples X Amount of Blood/CSF	Total Volume of Blood/CSF (mL)
Clinical laboratory (screening), includes serum alcohol test	1 x 15.5 mL	15.5
Clinical laboratory visits (plus 1 extra for follow-up, if needed)	5 x 10.5 mL	52.5
Blood sample for genotyping[a]	1 x 8.5 mL	8.5
PK (FRM-0334 and metabolites)	17 (19)[b] x 6 mL	102 (114)[b]
PD sample (PGRN)	20 (22)[b] x 6 mL	120 (132)[b]
PD samples (PGRN mRNA)	20 (22)[b] x 8 mL	160 (176)[b]
**Total volume of blood**		**458.5 (498.5)[b]**
CSF fluid collected for PD biomarkers, concentration of FRM-0334 and metabolites, and safety laboratory tests	2 x 16 mL	32
**Total volume of CSF fluid**		**32**

Abbreviations: CSF = cerebrospinal fluid; ICF = informed consent form; mRNA = messenger ribonucleic acid; PD = pharmacodynamic; PK = pharmacokinetic; PGRN = progranulin.

[a] Subjects will indicate on the ICF if they will allow this sample to be reserved and used for future research.

[b] Parenthetical values represent the number of samples and sample volume if optional PK and PD samples are collected

9.5.7 Other Safety Assessments

Columbia-Suicide Severity Rating Scale: A measurement of suicidality, a term referencing suicidal ideas or suicidal behaviors, is required by regulatory agencies in psychiatric subjects participating in clinical studies. The C-SSRS is a measure of suicidal ideations and suicidal behaviors (Posner et al, 2007), and will serve as an ongoing assessment of suicidality (ie, suicidal thinking and behavior) during the study.

The C-SSRS consists of 2 forms: a form measuring symptoms at screening (baseline/screening version) that includes a lifetime history version and a form measuring symptoms since the last study visit (since the last visit version). Subjects with a significant suicide risk as defined by a suicidal ideation as endorsed on items 4 or 5 on the C-SSRS within the past year, at screening or Day 1 predose (baseline), having suicidal behaviors detected by the C-SSRS within 2 years before screening, or investigator assessment are ineligible for the study.

The C-SSRS rater will complete training and will be certified in the administration of the scale to facilitate standardized administration and data collection. Raters who can produce verification of prior training (certificate) within the past 2 years are not required to be re-certified. Upon 2 years expiration of documented certification, raters will be required either to retake C-SSRS training or resubmit valid, current, documented certification.

A trained, certified C-SSRS rater will complete this scale at screening (using the baseline/screening version), predose on Day 1, postdose on Days 7, 14, and 28, telephone contact on Day 21, and follow-up (Day 38), or ET (using since the last visit version). Any change in the C-SSRS score indicating the presence of suicidality should be evaluated by the investigator for clinical significance, as described in Section 10.1.1.

10. ADVERSE EVENTS

The investigator is required to report to the Sponsor or the designee all AEs occurring during the study (21 Code of Federal Regulations [CFR] §312.64[b] and International Conference on Harmonisation (ICH) Good Clinical Practice (GCP) E6 [R1]). Adverse events observed at any time after the subject signs the ICF through the safety follow-up visit (Day 38 or ET, as applicable) are to be recorded. At each study visit, subjects will be evaluated for new AEs and the status of existing AEs. The investigator may elicit symptoms using an open-ended question, followed by appropriate questions that clarify the subject’s verbatim description of AEs or change in concomitant medications. The date and time of onset and resolution, determination of seriousness, severity, corrective treatment, outcome, and relationship to study drug will be recorded for all AEs.

Serious AEs must be reported to the Sponsor or the Sponsor representative within 24 hours of knowledge of their occurrence. Any SAE experienced by a subject that occurs after the subject signed the ICF and within 30 days of the last dose of study drug must be documented and reported. Subjects must be contacted by telephone 30 days (+2 days) after the last dose of study drug to collect SAE information. Completion of the 30-day safety telephone contact will be recorded in the eCRF. Any SAE information collected during the 30-day safety telephone contact or proactively reported to the site within 30 days after the last dose of study drug will be recorded, evaluated, and processed as described below.

Any AE that results in permanent discontinuation of study drug, whether serious or non-serious, should also be documented.

10.1 Definitions

10.1.1 Adverse Events

An AE is an untoward or medical occurrence associated with the use of study drug (FRM-0334 or placebo drug) in clinical investigation subjects, which does not necessarily have a causal relationship with the study drug (investigational product). An AE can be any

unfavorable and unintended symptom, sign, disease or condition, or test abnormality whether or not considered related to study drug. Adverse events that do not meet the definition for an SAE are considered non-serious events.

Adverse events include:

- Symptoms described by the subject or signs observed by the investigator or medical staff
- Test abnormalities (ie, laboratory tests, ECGs) that result in an alteration in medical care (diagnostic or therapeutic)

An AE may be reported by the subject (eg, a symptom or a disease associated with that symptom) or may be indicated by any test abnormalities (diagnostic or therapeutic). However, a test abnormality should be considered an AE only if it is assessed by the investigator as clinically significant and/or meets certain criteria, as indicated below:

- Physical examination: An abnormal physical examination finding, or adverse change in clinical status, should be considered as an AE, if the investigator assesses such an abnormality to be clinically significant; a subject complaint associated with such an abnormal finding should also be recorded as an AE
- 12-lead ECG: An abnormal 12-lead ECG finding should be recorded as an AE only if the investigator assesses such an abnormality to be clinically significant
- Laboratory tests: Abnormal laboratory test results are to be considered AEs if they are associated with clinical signs or symptoms assessed by the investigator to be clinically significant
- Vital signs: An abnormal vital sign value should be considered an AE only if the investigator assesses such an abnormality to be clinically significant

Note: Clinical significance of the physical examination, ECG, laboratory tests, and vital signs findings include those that are directly responsible for study discontinuation, require treatment or other therapeutic intervention, require further monitoring, and/or require further diagnostic evaluation (excluding a repetition of the same procedure to confirm the abnormality). All clinically significant findings will be recorded as AEs by the investigator.

Changes in safety evaluation scores (eg, C-SSRS) indicating worsening or exacerbation should be evaluated by the investigator for clinical significance, and if clinically significant (ie, some type of intervention required), an associated AE should be recorded. The AE recorded will be the primary underlying clinical manifestation deemed clinically significant (for which some action or intervention was required), not the change in the score itself.

10.1.1.1 Protocol-related Adverse Event

A protocol-related AE is an AE occurring during a clinical study that is not product related (either investigational or control), but is considered by the investigator to be related to the research conditions (ie, related to the fact that a patient is participating in the study). For example, a protocol-related AE may be an untoward event occurring during a washout period

of a treatment other than the study drug or an event related to a medical procedure required by the protocol.

10.1.2 Serious Adverse Events

An SAE is an AE that results in any of the following:

- Death: Subject died as the result of the event
- Life-threatening AE: An AE that places the subject, in the view of the investigator, at immediate risk of death from the AE as it occurred (ie, does not include an AE that had it occurred in a more severe form, might have caused death)
- Required or prolonged inpatient hospitalization: The AE resulted in an initial inpatient hospitalization or prolonged an existing hospitalization of the subject.
- Persistent or significant disability/incapacity: An AE that results in a substantial disruption of a person's ability to conduct normal life functions
- Congenital anomaly/birth defect: A congenital anomaly/birth defect that occurs in the offspring of a subject exposed to study drug
- Important medical events: An AE that may not result in death, be life-threatening, or require hospitalization may be considered an SAE when, based upon appropriate medical judgment, the event may jeopardize the subject and may require medical or surgical intervention to prevent one of the outcomes listed above

Note: Planned hospital admissions or surgical procedures for elective procedures or an illness or disease that existed before signing the ICF or before the subject was randomized will not be captured as SAEs. If planned admissions or procedures occur at a time other than what was planned (eg, due to an exacerbation in the preexisting illness or disease), they should be reported as SAEs.

Examples of such "important medical events" include allergic bronchospasm requiring intensive treatment in an emergency room or at home, blood dyscrasias or convulsions that do not result in inpatient hospitalization, or the development of drug dependency or drug abuse.

Medical and scientific judgment should be used in deciding whether expedited reporting is appropriate in other situations, such as with important medical events described above.

Events that meet SAE criteria must be recorded and reported regardless of expectedness or assessed association with study drug.

10.1.3 Unexpected Adverse Events

Any AE that is not consistent in specificity or severity with the current Investigator's Brochure, including all amendments, is considered unexpected.

10.2 Evaluation of Adverse Events and Serious Adverse Events

10.2.1 Severity

The investigator will assess the severity of each AE/SAE as mild, moderate, or severe, based on the following definitions. Note that severity is not the same as "seriousness," which is defined in Section 10.1.2 (and which serves as a guide for defining regulatory reporting obligations).

- Mild: Event that is usually transient and may require only minimal treatment or therapeutic intervention. The event does not generally interfere with usual activities of daily living
- Moderate: Event that is usually alleviated with additional specific therapeutic intervention. The event interferes with usual activities of daily living, causing discomfort, but poses no significant or permanent risk of harm to the subject
- Severe: Event that interrupts usual activities of daily living, or significantly affects clinical status, or may require intensive therapeutic intervention

10.2.2 Relationship to Study Drug

For each AE/SAE, the investigator will determine whether there is a reasonable possibility demonstrated by evidence, which suggests a causal relationship between study drug or study procedures and the AE. The relationship of adverse events to study drug and/or study procedures is categorized as Not Related, Possibly Related and Related. Definitions of each of these terms are below:

- Not related: There is no suspicion of a causal relationship between study drug and the AE; another cause of the event has been identified, no temporal association with study drug has been identified, or the study drug cannot be implicated
- Possibly related: There is some evidence supporting the possibility of a causal relationship between study drug and the AE; an alternative explanation (eg, concomitant drug or concomitant disease) is inconclusive, the temporal association with study drug is reasonable, and the causal relationship cannot be excluded
- Related: There is strong evidence that there is a causal relationship between study drug and the AE; AE cannot be reasonably explained by an alternative explanation (eg, concomitant drug or concomitant disease) and for which the temporal association with study drug is suggestive of a causal relationship
- Protocol related: Events believed to the related to study participation or a procedure required by the protocol

A relationship to study drug must be indicated for each AE/SAE recorded on the eCRF, even if there is only limited information at the time. The investigator may change his/her opinion of causality in light of follow-up information, amending the AE/SAE report accordingly. An AE is considered associated with study drug if the attribution is "possibly related" or "related."

10.2.3 Outcome

Outcome describes the status of the AE/SAE. The investigator will provide information regarding the subject outcome of each AE, and the options include:

- Fatal: Termination of life as a result of an AE
- Not recovered/not resolved: Subject has not recuperated or the AE has not improved
- Recovering/resolving: Subject is recuperating or the AE is improving
- Recovered/resolved: Subject has recuperated or the AE has resolved
- Recovered with sequelae/resolved with sequelae: AE has resolved, but the subject has symptoms or pathology
- Unknown: Unknown, not observed, not recorded, or refused

10.2.4 Action Taken Regarding Study Drug

The investigator will provide the action taken regarding study drug in response to each AE/SAE, and the options include:

- Dose not changed: No change in the administration of study drug
- Drug (study drug) interrupted: Temporary interruption (termination) in the administration of study drug
- Drug (study drug) withdrawn: Administration of study drug is terminated (no further dosing)
- Unknown: Unknown, not observed, not recorded, or refused

10.3 Timeframe for Adverse Events and Serious Adverse Events Collection

10.3.1 General

The investigator is required to report to the Sponsor or Sponsor representative all AEs occurring during the study (21 CFR §312.64[b] and ICH GCP E6 [R1]). An SAE, as defined in Section 10.1.2, must be reported to the Sponsor or Sponsor representative within 24 hours of knowledge of their occurrence according to the procedures outlined in Section 10.5. All AEs that result in permanent discontinuation of study drug, whether serious or non-serious must be reported.

Adverse events will be collected at each study visit from the time of signing of the ICF up to the safety follow-up visit on Day 38.

All SAEs occurring after the subject signs the ICF until 30 days after the last dose of study drug will be collected. All subjects must be contacted via telephone at 30 days (+2 days) after the last dose of study drug to collect SAE information. Completion of the 30-day safety telephone contact will be recorded in the eCRF. Any SAE information collected during the 30-day safety telephone contact or proactively reported by the subject to the site within 30 days after the last dose of study drug will be recorded, evaluated, and processed as described below.

10.3.2 Serious Adverse Events Experienced Following Subject Completion of the Study

If, at any time after the subject has completed the study (including the 30-day SAE telephone contact), the investigator or study staff becomes aware of an SAE assessed as possibly related or related to study drug (Section 10.2.2), the SAE and any known details must be reported promptly to the Sponsor or designee, as detailed in Section 10.5.

10.4 Recording of Adverse Events and Serious Adverse Events

All AEs/SAEs experienced by the subject will be recorded using medical terminology on the eCRF. Information including a concise description of the event; date and time of event onset and resolution; determination of seriousness, severity, corrective treatment, outcome, relationship to study drug; and action taken regarding study drug will be recorded. Abnormal results for vital signs, laboratory tests, and other safety assessments noted in Section 9.5 that meet the definition of an AE (Section 10.1.1) will be recorded. When possible, a diagnosis should be recorded as an AE, rather than symptoms or isolated laboratory abnormalities related to that diagnosis. A medical or surgical procedure is not an AE; rather the condition leading to the procedure should be recorded as the AE. If the condition is unknown, the procedure must be reported as an AE instead. Similarly, death is not an AE, but is rather the outcome of the AE(s) that resulted in death. If the AE(s) leading to death are unknown, then death must be reported as an AE/SAE.

All SAEs experienced by the subject will be recorded on an SAE report form and reported to the Sponsor or designee according to Section 10.5.

10.5 Reporting of Serious Adverse Events

The necessity and time requirements for reporting of SAEs to the Sponsor or designee and/or regulatory agencies are as follows:

- All SAEs and associated source documents must be reported in English within 24 hours of the investigator’s first knowledge of the event by fax or e-mail regardless of relationship to the study procedures or study drug. The investigator is requested to supply detailed information regarding the event at the time of the initial report. An SAE report form will be provided to the investigator for this purpose, and this form should be used to record pertinent information regarding the SAE.
- A completed SAE report form containing a detailed written description of the event along with additional supporting documents (eg, discharge letters, autopsy reports, and other documents) will be faxed within 48 hours of the investigator’s first knowledge of the event. (If faxed within 24 hours of the investigator’s first knowledge, this form may serve as the initial notification.)
- For the initial SAE notification report, the investigator must provide, at minimum, basic information such as the protocol number, subject's initials and date of birth (unless prohibited by local privacy laws), subject identification number, period of study drug

intake, event term, nature of the event, and the investigator's attribution regarding relatedness to study drug.

- In addition, the initial SAE report should include all pertinent known information about the SAE and the affected subject, such as the following: subject’s sex, weight, height, and ethnicity; description of the AE including reason for assessment as serious, severity, relationship to study drug, and potential alternative causes; relevant medical and surgical history and concomitant medication information; as well as study drug information including dates of dosing, rechallenge information, and action taken with study drug, as applicable. In addition, the investigator should provide a narrative to describe the course of events, including any treatments or relevant procedures.
- The fax is available for SAE reporting on a 24-hour basis and reviewed during normal business hours.
- Follow-up information, which may include copies of any relevant records and other documents for the subject not available at the time the initial SAE report form was completed, must be sent to as soon as available. Follow-up SAE reports may describe the evolution of the reported events and any new assessment of their outcome and/or relationship to study drug. Full supporting documentation will be solicited by the investigative site even if the SAE occurred at another institution. Such documentation may include copies of relevant subject/hospital records, and pathology or autopsy reports. For subject deaths, the cause of death is to be considered an SAE.
- Contact information for reporting an SAE is provided in a study manual.

10.6 Follow-Up of Adverse Events and Serious Adverse Events

All AEs/SAEs documented at a previous visit/contact that are designated as not recovered/not resolved or recovering/resolving will be reviewed by the investigator at subsequent visits/ contacts.

The investigator will provide follow-up information for any SAE as soon as it is available. The Sponsor or designee, or regulatory authorities, may request additional information regarding an SAE.

All AEs will be followed until resolution of the AE, completion of the subject’s participation, or study termination, whichever occurs first. Serious AEs will be followed until resolution, the condition stabilizes, or the investigator and Sponsor agree that follow-up is no longer necessary. Follow-up reports from the investigator must be provided as indicated using the SAE report form. Additional information (eg, hospital records, laboratory, or other diagnostic test results) should be provided if requested and/or indicated. Rules for AE/SAE follow-up apply to all subjects, including those who prematurely withdrew to the extent stated in the ICF. The investigator will ensure that follow-up includes further investigations consistent with appropriate medical management and subject consent to elucidate the nature and/or causality of the AE/SAE.

10.7 Reporting of Pregnancy

Female subjects will be instructed to notify the investigator immediately if they discover they are pregnant. Upon confirmation of pregnancy, female subjects will be discontinued from the study and followed for outcome. Male subjects will be instructed to notify the investigator immediately if they discover that their sexual partner is pregnant.

If the investigator learns of a report of pregnancy at any time after the subject signs the informed consent through 30 days after the last dose of study drug, the investigator will contact the Sponsor or designee within 24 hours, as instructed in Section 10.5; however, the investigator will complete the Pregnancy forms rather than the SAE forms, because healthy pregnancy is not an AE. The subject will be followed until the outcome of the pregnancy is known (eg, live birth or stillbirth).

The investigator will inform the subject that the Sponsor or designee is required to gather information regarding the course and outcome of the pregnancy after exposure to study drug. The progress of the pregnancy must be followed until the outcome of the pregnancy is known (ie, delivery, live birth or stillbirth, elective termination, or spontaneous abortion). If the pregnancy results in the birth of a child, additional follow-up information may be requested.

The investigator is requested to obtain follow-up information no later than 2 months after the gestational period to obtain maternal/fetal/neonatal outcome and any other relevant information. Follow-up information may be requested at additional time points. All study related visits/contacts involving a known pregnancy should include pregnancy status assessment until the pregnancy outcome is known.

Please note that pregnancy in and of itself is not an AE or an SAE. Pregnancy should not be entered into the eCRF as an AE unless the investigator suspects an interaction between study drug and the contraceptive method. Additionally, all information received will be assessed for any AEs and SAEs and processed per study guidelines. When a subject is discontinued because of pregnancy, pregnancy will be documented as the reason for study discontinuation.

Spontaneous abortions, elective terminations due to complications with the mother or fetus, maternal or fetal complications during labor or delivery that meet serious criteria, congenital anomalies, and stillbirths must be reported as SAEs.

10.8 Reporting to Institutional Review Boards, Independent Ethics Committees, and Regulatory Authorities

Investigators will receive copies of initial and follow-up expedited safety reports (unexpected SAEs that are determined to be associated with the use of study drug) from the Sponsor or designee. The investigator is responsible for fulfilling applicable local reporting

requirements to their Institutional Review Board/Independent Ethics Committee (IRB/IEC). Investigators must forward copies of the IRB/IEC notification to the Sponsor or designee.

In the US, the Sponsor will be responsible for notifying the Food and Drug Administration (FDA) of any suspected unexpected serious adverse reaction (ie, SUSAR); in the European Union (EU), the Sponsor or designee is responsible for notifying the European Medicines Agency, IEC, and competent authorities. In regions/countries other than the US/EU, reporting of events to IECs or local authorities will be performed by the investigator/ Sponsor or designee and in accordance with local procedures/regulations.

11. SUBJECT COMPLETION

11.1 Completion

A subject will be considered to have completed the study if he/she completes the study through Day 28 of double-blind treatment. The maximum number of days of exposure allowed for any subject is 28 days.

11.2 Withdrawal

In accordance with the Declaration of Helsinki and other applicable regulations, subjects are free to withdraw from the study at any time and without penalty or loss of future medical care, or any other benefits to which they are otherwise entitled. The investigator must discontinue any subject who requests to be withdrawn. The investigator may discontinue the subject's participation from the study for any of the following reasons:

- Adverse events (eg, intolerable AE or SAE)
- Occurrence of an exclusion criterion that is clinically relevant and affects the subject's safety, if discontinuation is considered necessary by the investigator and/or Sponsor
- Non-compliance with study procedures/inability to meet the study requirements
- Medication prohibited by the protocol
- Lost to follow-up
- Administrative or other reasons (ie, study cancellation or premature termination)

Female subjects with confirmed pregnancy must be withdrawn from the study and followed in accordance with Section 10.7 and as described in a study manual.

In case of premature withdrawal of a subject, the investigation should schedule an ET visit (Section 9.1.3.5 and Table 1); the 30-day SAE telephone contact should also be completed.

If a randomized subject is withdrawn or prematurely discontinues, the reason for withdrawal must be captured in the source document and eCRF. A study completion/termination eCRF must be completed for all randomized subjects only.

Lost to Follow-Up: If a subject is lost to follow-up, every effort must be made by study center personnel to contact the subject, inquire about the reason for discontinuation, and

follow-up any unresolved AEs/SAEs. All measures taken to contact the subject and information received during these attempts must be documented in the subject's medical record.

12. STATISTICAL METHODS

Statistical analyses will be conducted for safety, PD, PK, and other data using appropriate methods. A detailed statistical analysis plan (SAP) will be prepared for the final analyses.

Descriptive statistics will be presented for all analyses unless otherwise specified. For categorical variables, summary tabulations of the number and percentage within each category (with a category for missing data, if applicable) of the parameter will be presented. For continuous variables, data will be presented as number (n), mean, median, standard deviation, minimum, and maximum. Least squares (LS) means and geometric means will be provided for the appropriate tables.

Unless specified otherwise, all hypothesis testing will be 1-sided at an alpha level of 0.05, with no adjustments made for multiplicity. Missing data will not be imputed, unless specified otherwise for specific analyses. Statistical analyses will be performed using SAS® software version 9.3 or higher.

12.1 Sample Size Determination

A sample size of 15 subjects per sequential period (Group 1: 300 mg or placebo; Group 2: 500 mg or placebo) (n=12 FRM-0334 and n=3 placebo) will provide approximately 80% power to detect a 30% increase in plasma PGRN concentration (based on a 1-sided test, alpha=0.05).

12.2 Study Subjects

12.2.1 Analysis Populations

The following study populations will be evaluated:

- Safety population will include all randomized subjects who ingest at least 1 dose of study drug
- PD population will include all subjects in the safety population who have at least 1 postbaseline sample or clinical assessment
- PK population will include all subjects in the safety population who have sufficient plasma concentration data, as determined by the pharmacokineticist, for inclusion in the descriptive statistical analysis

The safety population will be used for the safety analysis, demographics and other baseline subject characteristics, PD population will be used for the PD and clinical assessments analyses, and the PK population will be used for the PK analysis.

12.2.2 Subject Disposition

The number and percentage of subjects screened, randomized, included in the safety, PD, and PK populations, and those who complete the study with assessments through Day 28 and through Day 38 (follow-up) will be presented by treatment group. The number and percentage of randomized subjects who discontinue the study and the primary reason for discontinuation will be summarized by treatment group. In addition, a separate enrollment and disposition summary table will be generated by country.

Study completion information, including the reason for premature study withdrawal, if applicable, will be presented by-subject in a data listing.

12.2.3 Demographics and Other Baseline Characteristics

Summary statistics for demographic and other baseline characteristics data will be provided by treatment group for the safety population.

Medical history will be summarized by treatment group and overall using system organ class (SOC) and preferred term. Events will be summarized by subject incidence rates, therefore, in any tabulation, a subject contributes only once to the count for a given preferred term.

Medical history findings including the verbatim term will be presented by-subject in a data listing. Demographic and baseline data will be provided by-subject in data listings.

12.2.4 Protocol Deviations

Protocol deviations will be finalized before unblinding and final database lock, and may include subjects who do not meet inclusion/exclusion criteria, ingest prohibited medications, do not complete the final Day 28 assessments, <75% compliance with double-blind study drug, or other significant protocol deviation as determined by the Medical Monitor and Sponsor. Protocol deviations will be presented by-subject in a data listing.

12.2.5 Prior and Concomitant Medications

Prior and concomitant medications will be separately summarized as the number and percentage of subjects by World Health Organization (WHO) drug class, preferred term, and treatment. Medications will be coded using the WHO drug dictionary, Anatomical Therapeutic Chemical (ATC) classes, and preferred term. Subjects will be counted only once for a given prior or concomitant medication.

Prior medications will be distinguished from concomitant medications by a stop date before the first dose of study drug. Any medication that stopped on the same day as the first dose of study drug will be considered a prior medication. If the stop date of a given medication is missing, then the medication will be classified as concomitant. Any medication that a subject started before the first dose of study drug and continued to take during the study and any

medication that the subject began taking after the first dose of study drug will be classified as concomitant.

Concomitant and prior medications will be presented by-subject in data listings.

12.2.6 Study Drug Exposure and Compliance

Exposure to study drug and treatment compliance will be summarized by treatment group for the safety population. Exposure to study drug will be calculated as (number of capsules dispensed less the number returned). Duration of exposure will be defined as the total number of days a subject was exposed to study drug, calculated as (date of last dose – date of first dose plus one day). If the date of the last dose of study drug is unknown, the last study drug return date will be used, if available; otherwise, the date of the last visit will be used.

Overall compliance will be calculated as:

- Group 1 FRM-0334 300 mg or placebo: study drug exposure in capsules/[duration of exposure in days x 3 capsules per day]) x 100, as a percentage
- Group 2 FRM-0334 500 mg or placebo: study drug exposure in capsules/[duration of exposure in days x 5 capsules per day]) x 100, as a percentage

Exposure to study drug, duration of exposure, and overall treatment compliance will be summarized. Study drug dosing information will be presented by-subject in data listings.

12.3 Pharmacokinetic Analyses

The plasma and CSF FRM-0334 and metabolite concentration data will be summarized for subjects in the PK population.

The individual subject plasma concentration-time data will be listed and displayed graphically on linear and log scales. The plasma concentration-time data will be summarized descriptively in tabular and graphical formats (linear and log scales). The noncompartmental PK parameters listed in Table 2 (after single dose) and Table 3 (after multiple dose) will be calculated. The PK parameter data will be listed and summarized descriptively in tabular format. The CSF concentration data will be summarized in tabular and graphical formats.

12.4 Pharmacodynamic Analyses

The plasma PD and CSF data will be summarized for subjects in the PD population. Baseline for the plasma PGRN and PGRN mRNA concentration will be the average of the samples obtained at screening, Day -7, and Day 1 predose. Baseline for PD CSF data and the 18F-FDG-PET scan will be the value obtained on Day -7.

The individual subject plasma PD data will be listed and displayed graphically on linear and log scales. The plasma PD concentration-time data will be summarized descriptively in tabular and graphical formats (linear and log scales). The plasma PD parameter data will be

listed and summarized descriptively in tabular and graphical formats. The CSF PD data will be summarized in tabular and graphical formats.

12.4.1 Primary Endpoint

The primary hypothesis being tested is that treatment with FRM-0334 300 or 500 mg daily, increases mean plasma PGRN concentrations over 28 days. The percent change from baseline to Day 28 (predose) will be descriptively summarized by treatment group. An analysis of the within-treatment group percent change from baseline to Day 28 (predose) in plasma PGRN will be performed using a mixed-effects model for repeated measures (MMRM), with fixed effects for treatment, time, treatment by time interaction, baseline as a covariate, and subject as a random effect.

For analysis purposes, ET visits will be assigned to the next scheduled visit.

12.4.2 Secondary Endpoints

The effect of FRM-0334 on plasma PGRN and PGRN mRNA will be assessed by comparing Day 1 and Day 28 postdose values to respective predose values, as well as to baseline:

- Day 7, Day 14, and Day 28 predose values will be compared to baseline
- Day 1 and Day 28 postdose values will be compared to respective Day 1 and Day 28 predose values
- Day 1 and Day 28 postdose values will be compared to baseline
- Average Day 1 and Day 28 postdose values (Day 1: AUC_{0-8})/8; Day 28 AUC_{0-24}/24) will be compared to baseline, as well as to the predose each day, and compared across treatments
- Baseline–corrected Day 1 and Day 28 AUC (AUC of individual baseline-corrected timepoints) will be determined and contrasted across treatments

Full details for the analyses for the above comparisons will be provided in the SAP.

The ratios of the Day 28 to Day -7 CSF PGRN levels will be determined and compared within and across treatment groups, using methods similar to the primary analysis.

12.5 Exploratory Analyses

12.5.1 Clinical Rating Assessments

Absolute change and percent change from baseline (Day 1 predose) to Day 28 for the FTD-CDR-SB and FRS will be summarized descriptively. The Day 28 ADCS-CGIC will be summarized descriptively. If sample size permits, within-group change from baseline for the FTD-CDR-SB, FRS and the Day 28 ADCS-CGIC will be analyzed using a one-sample t-test, and the comparison between treatment groups will be analyzed using an analysis of covariance (ANCOVA) model for the FTD-CDR-SB and FRS, with baseline score as a covariate, and with an ANOVA for the ADCS-CGIC.

12.5.2 Additional Analyses

12.5.2.1 Plasma PGRN

Sample size permitting, the comparison of the percent change from baseline in plasma PGRN between treatment groups will be analyzed as an exploratory endpoint, using an MMRM similar to above. This analysis will be performed for each active dose level compared to placebo, as well as combined active dose groups compared to placebo.

A "responder" analysis will also be conducted, with responders defined as subjects who experience any increase in plasma PGRN after baseline. The proportion of responders will be descriptively summarized, and compared between treatment groups using Fisher's exact test.

To explore the relationship between plasma PGRN and CSF PGRN, the correlation between plasma PGRN and CSF PGRN at Day -7 and Day 28 will be assessed using Pearson's correlation coefficient.

12.5.2.2 PD Exploratory Assessments

Assessments for CSF concentrations of t-tau, p-tau^{181}, Aβ42, NF-L and additional CSF biomarkers (eg, TDP-43), and regional cerebral glucose metabolism using 18F-FDG-PET and their respective change from baseline will be descriptively summarized. Magnetic resonance imaging with DICOM image will be used for anatomical mapping of the 18F-FDG-PET imaging analysis.

12.6 Safety Analysis

Safety data will be analyzed descriptively overall and by FRM-0334 dose, and no formal statistical comparisons will be performed. Data from unscheduled visits (laboratory tests, vital signs, physical examinations, and ECGs) will not be included in the tables, but will be included in the by-subject listings.

For the laboratory tests, vital signs, and ECG results, data for subjects who terminate early and have their ET visit recorded on Day 28 will have this visit recoded to the closest non-missing visit to the actual visit date.

12.6.1 Adverse Events

Adverse events will be collected and recorded from the time the subject signs the ICF to the safety follow-up visit on Day 38. Any SAE that occurs up to 30 days after the last dose of study drug will also be documented and reported. The verbatim AE term will be coded to the preferred term and SOC using terminology from the Medical Dictionary for Regulatory Activities (MedDRA).

A TEAE is defined as an AE that is first identified, or is identified to worsen in intensity, at a time point occurring after the first dose of study drug. The number and percentage of

subjects with any TEAE, with any TEAEs assessed by the investigator as related to study drug or to study procedures (not related, possibly related, or related), and severity (mild, moderate, severe), and with any treatment-emergent SAE will be summarized by treatment group and overall. In these tabulations, each subject will contribute only once (ie, the most related occurrence or the most intense occurrence) to each of the incidence rates in the descriptive analysis, regardless of the number of episodes.

All AEs and SAEs occurring during the study (pretreatment, treatment and follow-up) will be presented by-subject in data listings. By-subject listings will also be provided for deaths, SAEs, and AEs leading to study drug withdrawal.

12.6.2 Clinical Laboratory Tests

Laboratory tests values and changes from baseline over time will be summarized for each continuous clinical laboratory parameter. Shift tables showing the pattern of change from baseline versus each postbaseline visit will be presented.

All laboratory data will be presented by-subject in data listings. For hematology and chemistry laboratory data, the laboratory normal ranges will be provided, and individual abnormal laboratory values will be flagged and clinical significance will be indicated. Microscopic examination urinalysis data will be provided for subjects with a clinically significant positive result from the urinalysis dipstick evaluation.

12.6.3 Vital Signs

The actual value and change from baseline to each postbaseline assessment will be summarized for vital signs, including orthostatic measurements of systolic and diastolic blood pressure and pulse rates as well as respiration rate , temperature and weight. Height and body mass index will also be presented. Vital signs measurements will be presented by-subject in data listings.

12.6.4 Electrocardiogram

The following ECG results will be summarized:

- ECG values and changes from baseline for heart rate, PR interval, QRS interval, QT interval, QTc interval, and QTcF interval
- Number and percentage of subjects with overall assessment of normal, abnormal but not clinically significant, or abnormal and clinically significant
- Number and percentage of subjects with a QTcF interval of the following threshold limits: 451 to 480 msec, 481 to 499 msec, and ≥500 msec, and with a QTcF increase from baseline to the postbaseline visit within the categories of change of 31 to 60 msec and ≥60 msec

A listing of subjects with abnormal QTcF interval values (451 to 499 msec and ≥500 msec) and those with QTcF interval increase from baseline values (31 to 60 msec and >60 msec) will be provided. All ECG data will be presented by-subject in data listings.

12.6.5 Other Safety Assessments

Data from the C-SSRS will be descriptively summarized by treatment group. By-subject listings of the results will be provided.

12.7 Interim Analysis

No formal interim analysis is planned. A Safety Review Committee has been established for this study. It is composed of FORUM's Medical Officer, Head of Clinical Pharmacology, and Head of Pharmacovigilance, as well as the study Medical Monitor. The Medical Monitor will review accumulating safety data for both dose groups on an ongoing basis and will escalate findings to the Safety Review Committee if they meet the following criteria:

- white blood count <3000/mm^3
- platelet count <75,000/mm^3
- hemoglobin <8 g/dL
- ALT, AST, alkaline phosphatase, or GGT >3 x the upper limit of normal (ULN)
- bilirubin >1.5 x ULN
- creatinine >3 x the baseline value or >3 x ULN
- weight loss >10% between Day 1 and Day 28

An external pharmacokineticist will summarize unblinded plasma concentration data and associated non-compartmental PK parameters at designated times during the study, in accordance with the Safety Review Plan. However, all data provided by the external pharmacokineticist to the Sponsor will be provided in a manner that prevents unintentional unblinding of individual subject treatment assignments.

In addition to the ongoing review, after all subjects in Group 1 have completed (or discontinued) double-blind treatment, all safety (including AEs, clinical laboratory tests, ECGs, vital signs), tolerability, and any available PK data will be reviewed by the Safety Review Committee in a blinded fashion., Randomization of subjects into Group 2 (500 mg or placebo) will begin only after the 28-day, double-blind safety, tolerability, and available PK data for Group 1 (300 mg [n=12] or placebo [n=3]) are deemed acceptable by the Medical Monitor and the Safety Review Committee.

13. STUDY DRUG INFORMATION

13.1 Physical Description of Study Drug

The formulation of FRM-0334 used in this study will be a white, opaque, size No. 1, hard gelatin capsule. Study drug supplies will be provided to the investigator by the Sponsor or designee. Study drug capsules, FRM-0334 100 mg and placebo, will be identical in appearance (size- and color-matched) to facilitate blinding.

13.2 Packaging and Labeling

Capsule formulation and bulk packaging was conducted under current good manufacturing conditions. Study drug will be packaged in 60 mL high-density polyethylene bottles with child-resistant polyethylene caps, desiccant and a heat induction foil seal. Each study drug bottle will contain 30 capsules (100 mg FRM-0334 or matching placebo).

Affixed to each bottle of study drug will be a single-panel label or booklet label that may include the following information in the appropriate language: study number, unique bottle number, storage requirements, instructions for use, Investigational New Drug statement, expiration date, and name of the Sponsor.

Labels may have additional information or modification as required to meet local regulations. Bottles of study drug will be indistinguishable from each other, except for the unique study drug number included on each label.

On Day 1, the IXRS system will assign a randomization number and a study drug bottle number to that subject. This number will be used to identify the appropriate bottle(s) of study drug to be dispensed to that subject at each double-blind visit on Day 1 after the predose procedures, and Days 7 and 14 (except Day 28).

For Group 1 (FRM-0334 300 mg and placebo), a single bottle of study drug (30 capsules or a 1-week supply plus 9 extra capsules) will be dispensed to subjects on Days 1 and 7 and on Day 14, 2 bottles of study drug (60 capsules or a 2-week supply plus 18 extra capsules) will be dispensed. Subjects will ingest 3 x 100 mg capsules daily.

For Group 2 (FRM-0334 500 mg and placebo), 2 bottles of study drug (60 capsules or a 1-week supply plus 25 extra capsules) will be dispensed to subjects on Days 1 and 7 and on Day 14, 3 bottles of study drug (90 capsules or a 2-week supply plus 20 extra capsules) will be dispensed. Subjects will ingest 5 x 100 mg capsules daily.

13.3 Storage and Return of Study Drug

Study drug must be stored in a locked area or cabinet accessible only to appropriate study personnel at room temperature (59 to 77°F or 15 to 25°C).

Study drug bottles, including unused study drug, will be stored at the study center and subsequently returned to the Sponsor or designee. The investigator must provide an explanation for any missing bottles or unaccounted for capsules.

13.4 Drug Accountability

The investigator or designated study personnel will ensure study drug received is inventoried and accounted for throughout the study and that this information is recorded on a drug accountability log form, which will be maintained at the study center.

An accurate record of study drug supplies received will be maintained and include dates of receipt, shipment contents, and condition. The dispensing and return of study drug to and from subjects will be recorded on a dispensing log and on the appropriate drug accountability page of each subject's eCRF.

Subjects will be asked to return all study drug bottles dispensed, whether empty or containing unused study drug at their next study visit. The investigator will store the returned study drug bottles, and the contents of the bottles will not be combined. The returned bottles will not be re-dispensed.

Unless otherwise instructed by the Sponsor, the investigator will return all study drug bottles, whether empty or containing study drug, to the Sponsor or designee after inventory by the study monitor. The Sponsor or designee will ensure and document the proper disposition, according to local regulations, of study drug supplies, empty or full, with returned or unused drug, while maintaining the appropriate documentation to establish chain of custody.

A study monitor will verify study drug accountability during on-site monitoring visits. At the completion of the study, a study monitor must verify and reconcile that each subject received the correct study drug assignment (according to the randomization schedule) and that study drug bottles were correctly labeled.

The investigator will agree to store and/or dispense study drug only from location(s) agreed upon with the Sponsor. The investigator will agree to supply study drug only to sub-investigators, designated staff, and subjects participating in the study. Study drug will not be relabeled or reassigned to other subjects.

13.5 Additional Clinical Supplies

The investigator will be provided all required study-related supplies (including study manuals) before study initiation.

14. ETHICAL ASPECTS

14.1 Investigator Responsibilities

This study will be conducted in compliance with the protocol and in accordance with current US FDA regulations, ICH guidelines, and any other applicable regulatory requirements, as well as, GCP standards (CPMP/ICH/135/95); ethical principles that have their origin in the Declaration of Helsinki, and that are consistent with the principles of GCP; and, local ethical and legal requirements.

The investigator must adhere to the protocol as detailed in this document and must agree that any protocol changes must be approved by the Sponsor before seeking approval from the IRB/IEC. The investigator will be responsible for screening only those subjects who provide informed consent and enrolling only those subjects who meet all protocol eligibility criteria.

14.2 Institutional Review Board or Independent Ethics Committee

Screening of subjects (study initiation) will begin only after full approval of the protocol in its entirety has been obtained from a local IRB/IEC, and a copy of the approval has been received by the Sponsor.

A copy of the this protocol, ICF, relevant supporting information, and all subject recruitment or advertisement information must be submitted to the IRB/IEC for review and must be approved before the study is initiated. Any amendment(s) to the protocol must also be approved by the IRB/IEC before implementing study changes, unless for the welfare of the subject.

The investigator is responsible for informing the IRB/IEC of the progress of the study and any changes made to the protocol as deemed appropriate, at intervals stipulated in their guidelines, and at least once per year. The investigator agrees to make any required progress reports to the IRB/IEC, as well as reports of SAEs, life-threatening problems, or death.

14.3 Informed Consent

Each subject or legally acceptable representative must provide voluntary written informed consent (and sign other locally required documents) according to local requirements after the nature of the study has been fully explained. Each subject or legally acceptable representative must sign the ICF before any study-related activities are performed and before participating in the study. A copy of the signed ICF must be provided to the subject, and the original signed ICF must remain in each subject's study file and must be available for verification by the study monitor at any time.

Each subject's support person, if applicable, must sign an acknowledgement of responsibilities form in the study center before any study-related activities are performed for the support person, and a copy of the signed form must be provided to the support person

(first study-related activity for the support person will be performed at screening). The original signed acknowledgment of responsibilities document must remain in the subject's study file and must be available for verification by the study monitor at any time.

The ICF should be in accordance with the current revision of the Declaration of Helsinki and current ICH and GCP guidelines and local regulations. The Sponsor or designee will provide a draft sample ICF to the investigator. The Sponsor or designee must be involved in the review process before finalization of the draft version of the ICF. The final ICF must be approved by both the Sponsor and reviewing IRB/IEC. The final IRB/IEC-approved document must be provided to the Sponsor. Additionally, the Sponsor or designee will provide a draft sample acknowledgment of responsibilities form for the support person to the investigator. The Sponsor or designee must be involved in the review process before finalization of this form. The support person acknowledgment of responsibilities must be approved by the Sponsor and by the reviewing IRB/IEC. The final IRB/IEC-approved document must be provided to the Sponsor for regulatory purposes.

During the informed consent process, the investigator must explain to potential subjects the aims, methods, reasonably anticipated benefits, and potential hazards of the study and any discomfort it may entail. Subjects will be informed that they are free to not participate in the study and that they may withdraw consent to participate at any time. Subjects will be told which alternative treatments are available, if they refuse to participate and that such refusal will not prejudice future treatment. Subjects will also be told that their records may be examined by competent authorities and authorized persons, but that personal information will be treated as strictly confidential and will not be publicly available. Subjects must be given the opportunity to ask questions and a reasonable amount of time for consideration. After this explanation and before entry into the study, consent should be appropriately recorded by means of the subject's dated signature on the approved ICF. The subject should receive a signed and dated copy of the ICF.

14.4 Confidentiality

The information obtained during the conduct of this study is confidential, and disclosure to third parties other than those identified and involved in the study is strictly prohibited.

15. ADMINISTRATIVE REQUIREMENTS

Information obtained during the study will be used by the Sponsor in connection with the development of FRM-0334. The investigator is obliged to provide the Sponsor or designee with complete test results and all data collected during this study. This information may be disclosed to other physicians who are conducting similar studies and the US FDA or other regulatory authorities as deemed necessary by the Sponsor. Subject-specific information (eg,

laboratory test results) may be provided to other appropriate medical personnel (eg, primary care physician) only with the subject's permission.

Source documents are defined as the subject's medical records, results of original observations, and activities of clinical investigation. Source documents may include (but not limited to) consent forms, computer printouts, laboratory data, screening logs, and recorded data from automated instruments. All source documents produced in this study will be maintained by the investigator and made available for inspection by representatives of the Sponsor, US FDA, or other regulatory authorities. The signed ICF for each subject shall be filed with records kept by the investigator, and a copy will be given to each subject.

To ensure compliance with current Federal regulations, data generated by this study must be available for inspection upon request by representatives of the US FDA, national and local health authorities, the Sponsor and its designee, and the IRB/IEC.

15.1 Protocol Modifications

All protocol amendments must be issued by the Sponsor, signed and dated by the investigator, and should not be implemented without prior IEC or IRB approval, except where necessary to eliminate immediate hazards to subjects or when the change(s) involves only logistical or administrative aspects of the study (eg, change in monitor[s] or telephone number[s]). Responsibilities for reporting protocol amendments to any regulatory authority (if applicable) and/or IEC or IRB are described in the Ethical Aspects section (Section 14).

In situations requiring a departure from the protocol, the investigator or other physician in attendance will contact the Medical Monitor. If possible, this contact will occur before implementing any departure from the protocol. In all cases, contact with the Medical Monitor must occur as soon as possible in order to discuss the situation and agree on an appropriate course of action. The eCRF and source document will describe any departure from the protocol and the associated circumstances.

15.2 Subject Identification

The subject's number will be recorded on all source documentation and eCRF pages. Throughout screening, baseline, double-blind treatment, and follow-up, each subject will be identified by a unique subject number. Unique subject numbers will not be reassigned in the event of screen failures.

The investigator agrees to complete a subject identification register form, which will be used for the purpose of long-term follow-up, if needed. The form must be approved by the Sponsor. This form is confidential, and will be filed by the investigator in the study center file. Otherwise, all reports and communications relating to the study will identify subjects by their assigned number only.

15.3 Record Retention

The investigator must retain all study records according to ICH guidelines and according to the record retention policies of the country where the study is being conducted.

In compliance with ICH/GCP guidelines, the investigator/institution will maintain all CRFs and all source documents that support the data collected from each subject, and all study documents (essential documents for the conduct of a clinical study) as specified in applicable US FDA guidance (E6: GCP) or other applicable regulatory requirements.

The investigator/institution will take measures to prevent accidental or premature destruction of these documents. Essential documents must be retained, as directed by local regulations. At a minimum, essential documents must be retained for at least 2 years after the last approval of a marketing application in an US FDA or ICH region, or at least 2 years have elapsed since the formal discontinuation (FDA notification) of clinical development of FRM-0334 (21 CFR §312.57). These documents will be retained for a longer period if required by the applicable regulatory requirements or by an agreement with the Sponsor.

The investigator must consult with the Sponsor before disposal of any study records or of any change in the location, disposition, or custody of study records.

It is the responsibility of the Sponsor to inform the investigator/institution as to when these documents no longer need to be retained. If the responsible investigator retires, relocates, or for other reasons withdraws from the responsibility of keeping the study records, custody must be transferred to a person who will accept the responsibility. The Sponsor must be notified in writing of the name and address of the new custodian.

15.4 Case Report Form Completion

As used in this protocol, the term CRF should be understood to refer to either a paper form or an electronic data record or both, depending on the data collection method used in this study.

A CRF is required and should be completed for each included subject. The completed original CRFs are the sole property of FORUM Pharmaceuticals Inc. and should not be made available in any form to third parties without written permission from FORUM Pharmaceuticals Inc.

The investigator has ultimate responsibility for the collection and reporting of all clinical, safety and laboratory data entered on the CRFs and any other data collection forms (source documents) and ensuring that they are accurate, authentic / original, attributable, complete, consistent, legible, timely (contemporaneous), enduring and available when required. The CRFs must be signed by the investigator or by an authorized staff member to attest that the data contained on the CRFs is true. Any corrections to entries made in the CRFs, source

documents must be dated, initialed and explained (if necessary) and should not obscure the original entry.

In most cases, the source documents are the hospital's or the physician's subject chart. In these cases data collected on the CRFs must match the data in those charts. In some cases, the CRF, or part of the CRF, may also serve as source documents. In these cases, a document should be available at the investigator’s site as well as at FORUM Pharmaceuticals Inc. and clearly identify those data that will be recorded in the CRF, and for which the CRF will stand as the source document.

15.5 Study Completion and Study Termination

Study Completion: It is anticipated that each investigator will complete the last study visit for the last subject and submit all eCRFs in satisfactory compliance with the protocol and direction from the Sponsor or designee.

Study Termination: The Sponsor may terminate this study for any reason at any time without prior notice. Health Authorities, IECs or IRBs will be informed about the discontinuation of the study in accordance with applicable regulations. The investigator may terminate his/her participation in the study at any time.

15.6 Monitoring

An authorized representative (site monitor) of the Sponsor or designee will conduct study center visits to inspect study data, subjects’ medical records, and eCRFs in accordance with current US FDA regulations, ICH/GCP standards, and respective local, national government, and international regulations and guidelines. The investigator will permit authorized representatives of the Sponsor or designee, the US FDA, and appropriate health authorities to inspect facilities and records relevant to this study.

Study monitor visits may occur before study initiation, during the conduct of the study, and at the end of the study. The Sponsor or representative will perform on-site monitoring visits as frequently as necessary. Monitoring reports will be generated for each monitoring visit.

Monitors will confirm which documents will be considered source documents with the investigator before study start. During interim monitoring visits, the monitor will compare the data entered onto the eCRFs with the hospital or clinic records. At a minimum, source documentation must be available to substantiate proper informed consent procedures, adherence to protocol procedures, adequate reporting and follow-up of AEs, administration of concomitant medication, drug receipt/dispensing/return records, and study drug administration information. Findings from this review of eCRFs and source documents will be discussed with the investigator. The Sponsor expects that, during monitoring visits, the investigator (and as appropriate the study coordinator) will be available, the source

documentation will be available, and a suitable environment will be provided for review of study-related documents.

15.7 Data Quality Assurance

Steps will be taken to assure the accuracy and reliability of data. Such steps will include the selection of qualified investigators and appropriate study centers, review of protocol procedures and study-specific processes with investigators and associated personnel before study start, and periodic study center monitoring visits by the Sponsor or designee. Before study initiation, investigators and study center personnel will receive protocol- and study-specific training. Data management representatives will be available to provide assistance to study center personnel regarding entering subject data. The EDC system may include the use of protected fields and automatic checking functions for values with established parameters (eg, acceptable or exclusionary age) that are entered into certain eCRF fields. The Sponsor or designee will review data contained within eCRFs for accuracy and completeness during on-site monitoring visits and after submission to the Sponsor or designee and entry into the database. Identified discrepancies will be queried and resolved with the investigator or designee as indicated, and study center monitors and data management representatives will be involved in the query process.

15.8 On-Site Audits

Quality assurance representatives from the Sponsor or designee may visit the study center to conduct an audit in compliance with regulatory guidelines and company policy. Such audits will require access to all study records, including source documents, for inspection and comparison with data recorded in eCRFs. Subject privacy must be respected during such audits. Sufficient prior notice will be provided to allow the investigator to prepare properly for such an audit.

Similar auditing procedures may also be conducted by agents of any regulatory body reviewing the results of this study in support of a licensing application. The investigator should immediately notify the Sponsor upon contact with a regulatory agency concerning an upcoming inspection.

15.9 Use of Information and Publication

All information concerning FRM-0334, Sponsor operations, patent application, formulas, manufacturing processes, basic scientific data, and formulation information, supplied by the Sponsor to the investigator and not previously published, is confidential and remains the sole property of the Sponsor. The investigator agrees to use this information only to complete this study and not for other purposes without the Sponsor written consent.

The institution and investigator understand that the information developed in this study will be used by the Sponsor in connection with the continued development of FRM-0334, and

thus may be disclosed as required to other investigators, government regulatory agencies, or other scientific groups. To permit the information derived from this study to be used, the investigator is obligated to provide the Sponsor with all data obtained in the study. The Sponsor does not object to publication by investigator/institution of the results of the study based on information collected or generated by the investigator/institution, whether or not the results are favorable to the study drug, provided that such publication or other disclosure shall not occur until after completion of this study. However, to ensure against inadvertent disclosure of confidential information or unprotected inventions, the institution and investigator will provide to the Sponsor a copy of the proposed publication or other type of disclosure for review and comment at least 60 days before submission or otherwise disclosed.

At the Sponsor's request, the institution and investigator will delete from such publication or disclosure any confidential information other than study data generated by the institution. In addition, at the Sponsor's request, the institution and investigator will delay any publication or other disclosure for up to 90 days to enable the Sponsor to file a patent application on any invention described in such publication or other disclosure.

For this multicenter study, the institution agrees that the first publication will be a joint publication based on the full data set involving all centers, and, except as expressly set forth below, the institution and investigator will not make any publication or other disclosure of the results it has generated until after such joint publication. The investigator is free to decline to participate or be listed as an author in a joint publication. If a joint manuscript has not been submitted for publication within 24 months of the completion or termination of the study at all participating sites, the institution and investigator will be free to publish separately, subject to the other requirements of this Section and this Agreement.

16. REFERENCES

Abel T, Zukin RS. Epigenetic targets of HDAC inhibition in neurodegenerative and psychiatric disorders. Curr Opin Pharmacology 2008; 8(1): 57-64.

Baker M, Mackenzie IR, Pickering-Brown SM, et al. Mutations in progranulin cause tau-negative frontotemporal dementia linked to chromosome 17. Nature 2006; 442(7105): 916-919.

Boellaard R, O'Doherty MJ, Weber WA, et al. FDG PET and PET/CT: EANM procedure guidelines for tumour PET imaging: version 1.0. Eur J Nucl Med Mol Imaging 2010; 37: 181-200.

Cenik B, Sephton CF, Dewey CM, et al. Suberoylanilide hydroxamic acid (vorinostat) up-regulates progranulin transcription: rational therapeutic approach to frontotemporal dementia. J Biol Chem 2011; 286(18): 16101-16108.

Cruts M, Gijselinck I, van der Zee J, et al. Null mutations in progranulin cause ubiquitin-positive frontotemporal dementia linked to chromosome 17q21. Nature 2006; 442(7105): 920-924.

de Ruijter AJM, van Gennip AH, Caron HN, et al. Histone deacetylases (HDACs): characterization of the classical HDAC family. Biochem J 2003; 370(Pt 3): 737-749.

Dobson-Stone C, Hallupp M, Bartley L, et al. C9ORF72 repeat expansion in clinical and neuropathologic frontotemporal dementia cohorts. Neurology 2012; 79(10): 995-1001.

Eriksen JL, Mackenzie IR. Progranulin: normal function and role in neurodegeneration. J Neurochem 2008; 104(2): 287-297.

Fischer A, Sananbenesi F, Wang X, et al. Recovery of learning and memory is associated with chromatin remodelling. Nature 2007; 447(7141): 178-182.

Fischer A, Sananbenesi F, Mungenast A, et al. Targeting the correct HDAC(s) to treat cognitive disorders. Trends Pharmacol Sci 2010; 31(12): 605-617.

Gass J, Prudencio M, Stetler C, et al. Progranulin: an emerging target for FTLD therapies. Brain Res 2012; 1462: 118-128.

Ghidoni R, Stoppani E, Rossi G, et al. Optimal plasma progranulin cutoff value for predicting null progranulin mutations in neurodegenerative diseases: a multicenter Italian study. Neurodegener Dis 2012; 9(3): 121-127.

Hughes CP, Berg L, Danziger WL, et al. A new clinical scale for the staging of dementia. Br J Psychiatry 1982;140:566–572.

Knopman DS, Kramer JH, Boeve BF, et al. Development of methodology for conducting clinical trials in frontotemporal lobar degeneration. Brain 2008; 131(Pt 11): 2957-2968.

Mackenzie IR. The neuropathology and clinical phenotype of FTD with progranulin mutations. Acta Neuropathol 2007; 114(1): 49-54.

Mioshi E, Hsieh S, Savage S, et al. Clinical staging and disease progression in frontotemporal dementia. Neurology 2010; 74(20): 1591-1597.

Morris JC. The Clinical Dementia Rating (CDR): current version and scoring rules. Neurology 1993; 43(11): 2412-2414.

Posner K, Oquendo MA, Gould M, et al. Columbia Classification Algorithm of Suicide Assessment (C-CASA): Classification of suicidal events in the FDA's pediatric suicidal risk analysis of antidepressants. Am J Psychiatry 2007; 164(7):1035-1043.

Schneider LS, Olin JT, Doody RS, et al. Validity and reliability of the Alzheimer's Disease Cooperative Study - Clinical Global Impression of Change. The Alzheimer's Disease Cooperative Study. Alz Dis Assoc Disord 1997; 11(Suppl 2):S22-S32.

Spange S, Wagner, T, Heinzel, T, et al. Acetylation of non-histone proteins modulates cellular signaling at multiple levels. Int J Biochem Cell Biology 2009; 41(1):185-198.

Sun L, Eriksen JL. Recent insights into the involvement of progranulin in frontotemporal dementia. Curr Neuropharmacol 2011; 9(4): 632-642.

Sweatt JD. Behavioural neuroscience: Down memory lane. Nature 2007; 447(7141): 151-152.

Tully T, Bourtchouladze R, Scott R, et al. Targeting the CREB pathway for memory enhancers. Nat Rev Drug Discov 2003; 2(4): 267-277.

van Swieten JC, Heutink P. Mutation in progranulin (GRN) within the spectrum of clinical and pathological phenotypes of frontotemporal dementia. Lancet Neurol 2008; 7(10): 965-974.
