# Supplementary material for: Effect of the Histone Deacetylase Inhibitor FRM-0334 on Progranulin Levels in Patients With Progranulin Gene Haploinsufficiency: A Randomized Clinical Trial
Source: JAMA Netw Open. 2021 Sep 24;4(9):e2125584. doi: 10.1001/jamanetworkopen.2021.25584 (PMC8463943; doi:10.1001/jamanetworkopen.2021.25584)
Supplement: Supplement 2. — eFigure. Consort Diagram eTable 1. Clinical Sites and Corresponding PET and MRI Scanner Characteristics eTable 2. Predicted Change Over Time in Primary, Secondary, and Exploratory Measures eTable 3. Baseline Linear Regression Analyses Comparing Among Patient Characteristics and Outcome Measures eTable 4. Supplemental FDG-SUVR Sensitivity Analyses Using a More Stringent Voxel Threshold of PFWE < .05 eMethods eReferences [file jamanetwopen-e2125584-s002.pdf]

Supplementary Online Content

Ljubenkov PA, Edwards L, Iaccarino L, et al. Effect of the histone deacetylase inhibitor FRM-0334 on progranulin levels in patients with progranulin gene haploinsufficiency: a randomized clinical trial. *JAMA Netw Open*. 2021;4(9):e2125584. doi:10.1001/jamanetworkopen.2021.25584

eFigure. Consort Diagram

eTable 1. Clinical Sites and Corresponding PET and MRI Scanner Characteristics

eTable 2. Predicted Change Over Time in Primary, Secondary, and Exploratory Measures

eTable 3. Baseline Linear Regression Analyses Comparing Among Patient Characteristics and Outcome Measures

eTable 4. Supplemental FDG-SUVR Sensitivity Analyses Using a More Stringent Voxel Threshold of $P_{FWE} < .05$

eMethods

eReferences

This supplementary material has been provided by the authors to give readers additional information about their work.

© 2021 Ljubenkov PA et al. *JAMA Network Open.*

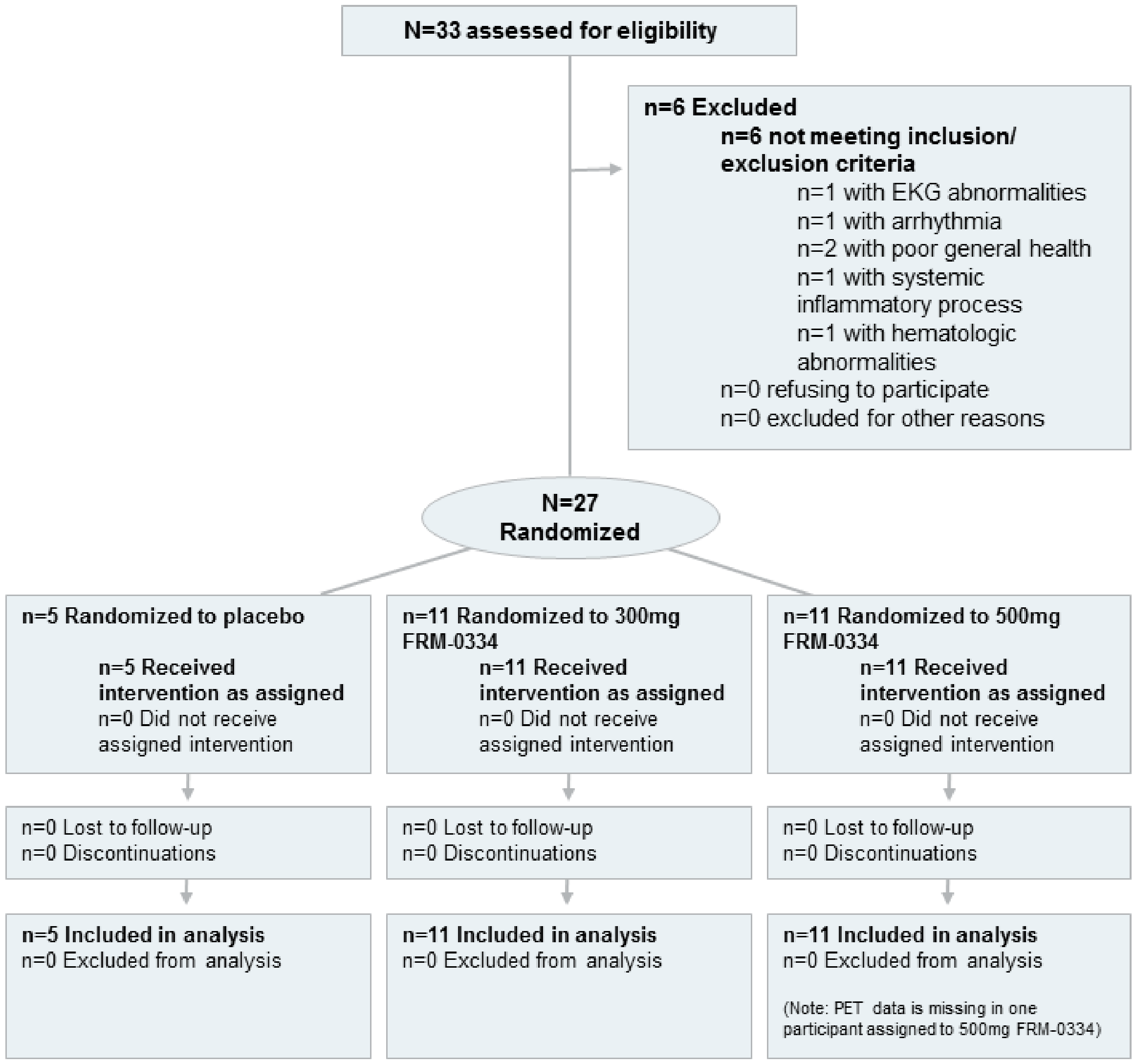

eFigure. Consort Diagram

© 2021 Ljubenkov PA et al. *JAMA Network Open.*

eTable 1. Clinical Sites and Corresponding PET and MRI Scanner Characteristics

	Summary of PET Collection Information (same for baseline and end of study unless otherwise noted)							Summary of MRI Collection Information			
	N with PET Data	PET Scanner Model	PET Frames	PET Frame Duration (ms)	PET Calculated Acquisition Time (minutes)	PET Slices Per Frame	PET Slice Thickness	N with MRI Data	MRI Scanner Model	3D T1 Series Description	Magnet Strength
Clinical Study Sites for *GRN* Mutation Carriers											
Hospital of the University of Pennsylvania, Philadelphia, Pennsylvania, United States of America	4	Camris Phillips Ingenuity TF	6	300000	30	90	2	3	Siemens Trio Tim	MP-RAGE	3
Mayo Clinic, Rochester, Minnesota	4	GE Medical Systems Discovery 690	6	300000	30	79	1.96	2	GE Medical Systems Signa HDxt	IR-SPGR	3
								1	GE Medical Systems Discovery MR750	MP-RAGE	3
								1	GE Medical Systems Discovery MR450	MP-RAGE	1.5
University of California, San Francisco, Memory and Aging Center, San Francisco, Unites States of America	1	GE Medical Systems Discovery STE	6	300000	30	47	3.27	1	Siemens Trio Tim	MP-RAGE	3
Azienda Ospedaliera Spedali Civili di Brescia, Brescia, Italy	5	GE Medical Systems Discovery 690	6	300000	30	47	3.27	4	Siemens Trio Avanto	MP-RAGE	1.5
								1	Siemens Skyra	MP-RAGE	3
Laboratory of Alzheimer's Neuroimaging and Epidemiology - LANE, IRCCS Istituto Centro San Giovanni di Dio Fatebenefratelli, Brescia, Italy	1	Siemens Biograph 40 mCT	6	300000	30	74	3	1	GE Medical Systems Signa HDxt	IR-SPGR	1.5
Hôpital Pitié-Salpêtrière, Paris, France	1	Siemens 1094	4 at baseline (6 at end of study)	300000	20 at baseline (30 at end of study)	56	4	1	Siemens Verio	MP-RAGE	3
CNR-MAJ / Rouen University Hospital, Rouen, France	1	GE Medical Systems Discovery 710	6	300000	30	47	3.27	1	GE Medical Systems Discovery MR750	BRAVO IR	3
Erasmus University Medical Center, Rotterdam, Netherlands	4	Siemens Biograph 128	6	300000	30	74	3	4	Philips Medical Systems Achieva	MP-RAGE	3
University Hospitals Leuven, Leuven, Belgium	2	Siemens 1080	6	300000	30	82	2	2	Philips Medical Systems Ingenia	MP-RAGE	3
The National Hospital for Neurology and Neuroscience, London, England	1	GE Medical Systems Discovery STE	6	300000	30	47	3.27	1	Siemens Trio Tim	MP-RAGE	3
	1	GE Medical Systems Discovery 710	1 at baseline (6 at end of study)	900000 at baseline (300000 at end of study)	15 at baseline (30 at end of study)	47	3.27	1	Siemens Prisma Fit	MP-RAGE	3
Memory Resource and Research Center of Lille, CHRU de Lille, Hôpital Roger Salengro, Lille, France	2	Siemens Biograph 20 mCT	6	300000	30	109	2.027	2	Philips Medical Systems Achieva	MP-RAGE	3
Clinical Study Site for Age-Matched Healthy Controls											
Lawrence Berkeley National Laboratory, Berkeley, United States of America	52	Siemens Biograph 6 Truepoint PET/CT	6	300000	30	109	2.027				

© 2021 Ljubenkov PA et al. *JAMA Network Open.*

eTable 2. Predicted Change Over Time in Primary, Secondary, and Exploratory Measures

Outcome Designation	Measure of interest	Available Participant Data			Predicted Daily Change in Participants Randomized to FRM-0334			Additional Change in Participants Randomized to FRM-0334 Relative to Placebo			Additional Change with Each hr*ng/ml Increase in Baseline (Day 1) FRM-0334 Plasma AUC			Additional Change with Each ng/ml Increase in Baseline (Day 1) FRM-0334 Plasma C_{max}		
		Placebo (N)	FRM-0334 (N)	Average number of time points per participant	units/day	P	95% CI	units/day	P	95% CI	units/day	P	95% CI	units/day	P	95% CI
Co-primary	Plasma progranulin (pg/ml)	5	22	5	4.3	0.558	(-10, 18)	-4.x10^+0	0.74	(-3.x10^+1, 2.2x10^+1)	-3.x10^-3	0.496	(-1.x10^-2, 6.0x10^-3)	-1.x10^-2	0.482	(-5.x10^-2, 2.5x10^-2)
Co-secondary	CSF progranulin (pg/ml)	3	21	1.9	$4.1x10^{-1}$	0.128	(-1.x10^-1, 9.5x10^-1)	-5.x10^-1	0.459	(-1.x10^+0, 8.7x10^-1)	-1.x10^-4	0.261	(-4.x10^-4, 1.3x10^-4)	-8.x10^-4	0.274	(-2.x10^-3, 6.3x10^-4)
Exploratory	CSF NfL (pg/ml)	4	22	1.9	2.2	0.187	(-1, 5.6)	-1.x10^+1	0.101	(-2.x10^+1, 2.1x10^+0)	-7.x10^-4	0.52	(-2.x10^-3, 1.4x10^-3)	-3.x10^-3	0.405	(-1.x10^-2, 5.3x10^-3)
	CSF $A\beta_{1\text{-}42}$ (pg/ml)	4	22	1.9	$6.4x10^{-1}$	0.267	(-4.x10^-1, 1.8x10^+0)	1.0x10^+0	0.594	(-2.x10^+0, 5.0x10^+0)	6.9x10^-5	0.845	(-6.x10^-4, 7.6x10^-4)	-3.x10^-4	0.81	(-3.x10^-3, 2.8x10^-3)
	CSF p-tau_{181} (pg/ml)	3	22	1.9	1	0.345	(-1, 3.1)	7.9x10^-1	0.779	(-4.x10^+0, 6.3x10^+0)	2.2x10^-5	0.973	(-1.x10^-3, 1.3x10^-3)	-1.x10^-3	0.724	(-6.x10^-3, 4.8x10^-3)
	CSF total tau (pg/ml)	4	22	2	-8.x10^-1	0.416	(-2.x10^+0, 1.1x10^+0)	-1.x10^+0	0.577	(-6.x10^+0, 3.3x10^+0)	-2.x10^-5	0.969	(-1.x10^-3, 1.2x10^-3)	4.3x10^-4	0.881	(-5.x10^-3, 6.1x10^-3)
	CDR® plus NACC FTLD sum of boxes	3	18	1.9	2.2x10^-2	0.057	(-6.x10^-4, 4.5x10^-2)	1.6x10^-2	0.569	(-4.x10^-2, 7.4x10^-2)	1.0x10^-5	0.127	(-3.x10^-6, 2.4x10^-5)	5.6x10^-5	0.059	(-2.x10^-6, 1.1x10^-4)
	FRS (%)	3	12	1.9	-8.x10^-2	0.122	(-1.x10^-1, 2.2x10^-2)	-4.x10^-2	0.663	(-2.x10^-1, 1.6x10^-1)	-1.x10^-5	0.697	(-9.x10^-5, 6.4x10^-5)	-4.x10^-6	0.985	(-4.x10^-4, 4.5x10^-4)
	Bifrontal FDG-SUVR	5	21	2	-4.x10^-4	0.052	(-8.x10^-4, 2.8x10^-6)	3.2x10^-4	0.523	(-6.x10^-4, 1.3x10^-3)	-2.x10^-7	0.026	(-4.x10^-7, -3.x10^-8)	-1.x10^-6	0.04	(-2.x10^-6, -4.x10^-8)

© 2021 Ljubenkov PA et al. *JAMA Network Open*.

eTable 3. Baseline Linear Regression Analyses Comparing Among Patient Characteristics and Outcome Measures						
Model Information			Results			
Dependent Variable	Independent variable of interest	Additional Covariates in the Model	b	95% Confidence Interval	P Value	R^2 for the model
FRM-0334 Visit 1 AUC	Age (years)	N/A	84.1	(20.2, 148)	0.012	0.2737
FRM-0334 Visit 1 Cmax	Age (years)	N/A	23.3	(10.2, 36.5)	0.001	0.4064
FRM-0334 Visit 7 AUC	Age (years)	N/A	125.2	(22.5, 227.9)	0.019	0.2442
FRM-0334 Visit 7 Cmax	Age (years)	N/A	21.0	(7.9, 34.2)	0.003	0.3576
FRM-0334 Visit 3 AUC	CDR® plus NACC FTLD sum of boxes	N/A	81.6	(-45.9, 209)	0.194	0.1032
FRM-0334 Visit 3 Cmax	CDR® plus NACC FTLD sum of boxes	N/A	25.2	(-2.1, 52.5)	0.068	0.1926
FRM-0334 Visit 7 AUC	CDR® plus NACC FTLD sum of boxes	N/A	82.1	(-131.1, 295.2)	0.426	0.04
FRM-0334 Visit 7 Cmax	CDR® plus NACC FTLD sum of boxes	N/A	17.8	(-10.1, 45.7)	0.196	0.1023
FRM-0334 Visit 7 AUC	FRM-0334 Day 3 AUC	N/A	1.27	(0.83, 1.71)	<0.0005	0.6489
FRM-0334 Visit 7 Cmax	FRM-0334 Day 3 Cmax	N/A	0.35	(-0.06, 0.77)	0.093	0.1345
Bifrontal FDG-SUVR	Plasma progranulin (pg/ml)	Age, Sex	-1.x10^-5	(-5.x10^-5, 2.5x10^-5)	0.493	0.469
Bifrontal FDG-SUVR	CSF progranulin (pg/ml)	Age, Sex	-2.x10^-4	(-1.x10^-3, 6.7x10^-4)	0.578	0.44
Bifrontal FDG-SUVR	CSF NfL (pg/ml)	Age, Sex	-9.2x10^-5	(-1.3x10^-4, -5.6x10^-5)	<0.0005	0.7918
Bifrontal FDG-SUVR	CSF NfL (pg/ml) - additional sensitivity analysis	Age, Sex, & (parenchymal volume/TIV)	-3.x10^-5	(-8.x10^-5, 6.8x10^+6)	0.09	0.8666
Bifrontal FDG-SUVR	CSF $A\beta_{1-42}$ (pg/ml)	Age, Sex	2.6x10^-4	(-1.x10^-4, 7.2x10^-4)	0.247	0.5193
Bifrontal FDG-SUVR	CSF p-tau_{181} (pg/ml)	Age, Sex	-6.x10^-4	(-2.x10^-3, 8.4x10^-4)	0.394	0.5573
Bifrontal FDG-SUVR	CSF total tau (pg/ml)	Age, Sex	-7.2.x10^-4	(-1.4.x10^-3, -9.5x10^-5)	0.026	0.6017
Bifrontal FDG-SUVR	CSF total tau (pg/ml) - additional sensitivity analysis	Age, Sex, & (parenchymal volume/TIV)	2.2x10^-5	(-4.x10^-4, 5.3x10^-4)	0.926	0.846
Bifrontal FDG-SUVR	CDR® plus NACC FTLD sum of boxes	Age, Sex	-3.6x10^-2	(-4.9x10^-2, -2.2x10^-2)	<0.0005	0.8068
Bifrontal FDG-SUVR	CDR® plus NACC FTLD sum of boxes - additional sensitivity analysis	Age, Sex, & (parenchymal volume/TIV)	-1.x10^-2	(-3.x10^-2, -4.x10^-4)	0.045	0.8849
Bifrontal FDG-SUVR	Clinical Global Impression Baseline Severity (CGI-S)	Age, Sex	-1.x10^-1	(-1.x10^-1, -6.x10^-2)	<0.0005	0.7583
Bifrontal FDG-SUVR	Frontotemporal Dementia Rating Scale (FRS) %	Age, Sex	8.8x10^-3	(3.7x10^-3, 1.3x10^-2)	0.004	0.7804
Log Plasma progranulin (pg/ml)	CDR® plus NACC FTLD sum of boxes	Age, Sex	9.6x10^-4	(-2.x10^-2, 2.5x10^-2)	0.935	0.032
Log CSF progranulin (pg/ml)	CDR® plus NACC FTLD sum of boxes	Age, Sex	5.2x10^-3	(-2.x10^-2, 3.4x10^-2)	0.707	0.0495
Log CSF NfL (pg/ml)	CDR® plus NACC FTLD sum of boxes	Age, Sex	8.8x10^-2	(1.9x10^-2, 1.5x10^-1)	0.016	0.7172
CSF $A\beta_{1-42}$ (pg/ml)	CDR® plus NACC FTLD sum of boxes	Age, Sex	-1.x10^+1	(-3.x10^+1, 6.6x10^+0)	0.194	0.3894
Log CSF p-tau_{181} (pg/ml)	CDR® plus NACC FTLD sum of boxes	Age, Sex	1.7x10^-2	(-3.x10^-2, 6.9x10^-2)	0.505	0.1218
Log CSF total tau (pg/ml)	CDR® plus NACC FTLD sum of boxes	Age, Sex	3.0x10^-2	(-1.x10^-2, 7.5x10^-2)	0.167	0.3799
Log CSF progranulin (pg/ml)	Plasma progranulin (pg/ml)	Age, Sex	6.2x10^-5	(2.4x10^-5, 1.0x10^-4)	0.003	0.4471
Log CSF NfL (pg/ml)	Plasma progranulin (pg/ml)	Age, Sex	7.0x10^-5	(-8.x10^-5, 2.2x10^-4)	0.35	0.5883
Log CSF total tau (pg/ml)	Plasma progranulin (pg/ml)	Age, Sex	2.8x10^-5	(-5.x10^-5, 1.1x10^-4)	0.476	0.3065
Log CSF NfL (pg/ml)	CSF progranulin (pg/ml)	Age, Sex	-1.x10^-4	(-3.x10^-3, 3.2x10^-3)	0.913	0.5676
Log CSF total tau (pg/ml)	CSF progranulin (pg/ml)	Age, Sex	1.1x10^-3	(-7.x10^-4, 2.9x10^-3)	0.219	0.3323
Log CSF total tau (pg/ml)	CSF NfL (pg/ml)	Age, Sex	1.1x10^-3	(-7.x10^-4, 2.9x10^-3)	0.219	0.3323
Log CSF total tau (pg/ml)	CSF p-tau_{181} (pg/ml)	Age, Sex	2.8x10^-5	(-5.x10^-5, 1.1x10^-4)	0.476	0.3065

Additional Linear Regression Analyses Comparing Change in PGRN to Change Other Measures After Treatment with FRM-0334 (high and low dose)						
Model Information			Results			
Dependent Variable	Independent variable of interest	Additional Covariates in the Model	b	95% Confidence Interval	P Value	R^2 for the model
Change in Bifrontal FDG-SUVR	Change in Plasma progranulin (pg/ml)	Age, Sex	-9.2x10^-6	(-2.6x10^-5, 7.9x10^-6)	0.263	0.203
Change in CSF NfL (pg/ml)	Change in Plasma progranulin (pg/ml)	Age, Sex	1.6x10^-1	(-1.7x10^-2, 3.3x10^-1)	0.072	0.3404
Change in CSF $A\beta_{1-42}$ (pg/ml)	Change in Plasma progranulin (pg/ml)	Age, Sex	-1.4x10^-2	(-4.9x10^-2, 2.0x10^-2)	0.378	0.2119

© 2021 Ljubenkov PA et al. *JAMA Network Open.*

Change in p-tau$_{181}$ (pg/ml)	Change in Plasma progranulin (pg/ml)	Age, Sex	2.9x10^-2	(-7.7x10^-2, 1.3x10^-1)	0.562	0.1903
Change in CSF total tau (pg/ml)	Change in Plasma progranulin (pg/ml)	Age, Sex	2.6x10^-3	(-1.1x10^-1, 1.1x10^-1)	0.958	0.078
Change in CDR® plus NACC FTLD sum of boxes	Change in Plasma progranulin (pg/ml)	Age, Sex	5.3x10^-4	(-8.3x10^-4, 1.9x10^-3)	0.404	0.2347
Change in FRS	Change in Plasma progranulin (pg/ml)	Age, Sex	-9.9x10^-4	(-8.0x10^-3, 6.0x10^-3)	0.742	0.0812
Change in Bifrontal FDG-SUVR	Change in CSF progranulin (pg/ml)	Age, Sex	6.6x10^-5	(-5.8x10^-4, 7.1x10^-4)	0.83	0.084
Change in CSF NfL (pg/ml)	Change in CSF progranulin (pg/ml)	Age, Sex	-2.0	(-6.0, 2.1)	0.317	0.1093
Change in CSF Aβ$_{1-42}$ (pg/ml)	Change in CSF progranulin (pg/ml)	Age, Sex	-7.9x10^-1	(-1.8, 1.7x10^-1)	0.101	0.2546
Change in p-tau$_{181}$ (pg/ml)	Change in CSF progranulin (pg/ml)	Age, Sex	-2.3	(-4.2, -3.8x10^-1)	0.022	0.4131
Change in CSF total tau (pg/ml)	Change in CSF progranulin (pg/ml)	Age, Sex	2.0	(-1.1, 4.1)	0.062	0.2682
Change in CDR® plus NACC FTLD sum of boxes	Change in CSF progranulin (pg/ml)	Age, Sex	5.3x10^-4	(-8.3x10^-4, 1.9x10^-3)	0.404	0.2347
Change in FRS	Change in CSF progranulin (pg/ml)	Age, Sex	3.8x10^-2	(-1.2x10^-1, 1.9x10^-1)	0.579	0.0722

© 2021 Ljubenkov PA et al. *JAMA Network Open.*

eTable 4. Supplemental FDG-SUVR Sensitivity Analyses Using a More Stringent Voxel Threshold of $P_{FWE}<0.05$
Note: Our Primary PET analysis (Figures 2 & 3) used a cluster threshold of $P_{FWE}<0.05$ and voxel level $P_{unc}<0.001$

All *GRN* Mutation Carriers (N=26) vs Age-Matched Controls (N=52) (Voxelwise ANCOVA controlling for age & sex))

Laterality	Brain Region	Brodmann Area	Cluster p (FWE corrected)	peak voxel p (FWE corrected)	t Value	MNI coordinate (mm)		
						x	y	z
Left	Anterior Cingulate	32	<0.0005	<0.0005	7.93	-2	38	24
Left	Dorsolateral Prefrontal	8		<0.0005	7.53	-36	22	48
Left	Dorsal Prefrontal	9		<0.0005	7.49	-8	52	42
Left	Angular Gyrus	39	<0.0005	0.001	5.78	-58	-58	38
Left	Supramarginal Gyrus	40		0.003	5.52	-58	-44	44
Left	Angular Gyrus	39		0.005	5.36	-42	-68	52
Left	Thalamus	-	0.03	0.004	5.38	-4	-16	2
Left	Caudate Head	-	0.048	0.005	5.37	18	8	10
Left	Middle Temporal Gyrus	21	0.063	0.011	5.12	-64	-24	-16
Left	Inferior Temporal Gyrus	20		0.013	5.07	-58	-30	-24
Left	Temporal Pole	20	0.034	0.012	5.1	-46	6	-42
Left	Inferior Frontal Gyrus	6		0.014	5.05	-60	0	-24
Left	Temporal Pole	20		0.027	4.84	-54	-6	-36
Left	Temporal Pole	20	0.292	0.022	4.92	-4	-60	34
Right	Angular Gyrus	39	0.895	0.04	4.72	56	-58	46
Left	Middle Temporal Gyrus	21	0.839	0.045	4.68	-58	-36	-4

Symptomatic *GRN* Mutation Carriers (N=18) vs Age-Matched Controls (N=52) (Voxelwise ANCOVA controlling for age & sex))

Laterality	Brain Region	Brodmann Area	Cluster p (FWE corrected)	peak voxel p (FWE corrected)	t Value	MNI coordinate (mm)		
						x	y	z
Left	Dorsal Prefrontal	9	<0.0005	<0.0005	8.28	-10	54	42
Left		8		<0.0005	7.85	-12	38	52
Left	Supplementary Motor	6		<0.0005	7.77	-20	26	56
Left	Dorsal Prefrontal	8	0.003	<0.0005	6.62	-12	8	6
Left	Angular Gyrus	39	<0.0005	0.001	5.88	-56	-56	40
Left				0.002	5.72	-50	-56	46
Left				0.002	5.62	-58	-46	44
Right	Caudate Head	-	0.14	0.009	5.27	18	8	10
Left	Thalamus	-	0.157	0.012	5.17	-4	-26	2
Left	Middle Temporal Gyrus	21	0.558	0.031	4.87	-66	-24	-16
Right	Medial Orbitofrontal	11	0.889	0.048	4.73	12	12	-18

FDG-SUVR vs CDR® plus NACC FTLD sum of boxes in all (N=26) *GRN* mutation Carriers
(Voxelwise Regression Analysis controlling for age & sex)

Laterality	Brain Region	Brodmann Area	Cluster p (FWE corrected)	peak voxel p (FWE corrected)	t Value	MNI coordinate (mm)		
						x	y	z
Right				0.005	8.2	30	58	26
	Dorsolateral Prefrontal	10	0.002	0.045	6.51	20	62	28
		10	0.031	0.033	6.73	-34	52	28
Left				0.034	6.71	-16	36	58
	Dorsolateral Prefrontal	8	0.017	0.035	6.69	-18	44	48

FDG-SUVR vs CSF NfL in all (N=26) *GRN* mutation Carriers
(Voxelwise Regression Analysis controlling for age & sex)

Laterality	Brain Region	Brodmann Area	Cluster p (FWE corrected)	peak voxel p (FWE corrected)	t Value	MNI coordinate (mm)		
						x	y	z
Left	Medial Orbitofrontal	11	<0.0005	0.004	7.32	-2	30	-22
				0.006	7.09	-2	46	-20
				0.017	6.51	-8	54	-22
	Lateral Orbitofrontal	10	<0.0005	0.005	7.27	-38	42	0
Right		10	0.016	0.023	6.32	46	54	-10
	Frontal Pole		0.031	0.036	6.06	32	64	-4
	Medial Prefrontal		0.017	0.037	6.05	10	62	-20
	Orbitofrontal			0.041	5.99	22	62	-18
Left	Lateral Orbitofrontal	47	0.03	0.044	5.95	-28	38	-16
	Dorsomedial Prefrontal	10	0.043	0.048	5.89	-12	62	14

© 2021 Ljubenkov PA et al. *JAMA Network Open.*

eMethods

Specific sites of participant recruitment

Hospital Roger Salengro (Lillle, France), Hôpital Charles-Nicolle (Rouen, France), Erasmus Medical Center (Rotterdam, Netherlands), Hospital of the University of Pennsylvania (Philadelphia, United States of America), IRCCS Istituto Centro San Giovanni Di Dio Fatebenefratelli (Brescia, Italy), The National Hospital for Neurology and Neuroscience (London, England), Mayo Clinic, Rochester (United States of America, University of California), San Francisco, Memory and Aging Center (San Francisco, United States of America), Pitié-Salpêtrière Hospital (Paris, France), Spedali Civili di Brescia (Brescia, Italy), and University Hospitals Leuven (Leuven, Belgium).

Fluid Biomarkers analysis:

Samples for PK and PD assessments were collected in polypropylene tubes and stored a -70°C prior to analysis. Assays run contemporaneously at the end of the study by central laboratories in order to minimize batch effect.

Plasma progranulin, cerebrospinal (CSF) fluid progranulin, and plasma FRM-0334 were measures by a contract research organization (CRO), ICON Laboratory Services, Whitesboro, NY. This BioVendor Enzyme Linked ImmunoSorbent Assay (ELISA) Kit was utilized to quantify progranulin in human plasma (K_2EDTA) and human CSF. The kit employed for this assay is for human serum/plasma and adapted for use with human CSF. In this ELISA method, the sample plate provided by the kit contains human progranulin antibody immobilized onto removable microwells. The standards (provided by the kit), samples, blanks, and diluted QCs are added to the appropriate wells of the sample plate already containing the Antibody Conjugate AK, provided by the kit, and incubated at ambient temperature for approximately 1 hour. The plate is then washed and an Enzyme Conjugate EK, provided by the kit, is added to appropriate wells and incubated for approximately 30 minutes. After the final wash step, Substrate Solution S, provided by the kit, is added to the plate. After an incubation of approximately 30 minutes, in the dark, the reaction is stopped with the Stopping Solution SL, also provided by the kit. Color develops in proportion to the amount of progranulin present. Plates are read on a plate reader using two filters (450 nm for detection and 620 nm for background). Progranulin concentrations are determined on a standard curve obtained by plotting optical density (OD) versus concentration using a four-parameter logistic curve-fitting program. The calibration curve range of this method is 18.8 pg/mL – 2500 pg/mL. Plasma FRM-0334 was measured via liquid chromatography/ tandem mass spectrometry using previously validated methods. Samples for plasma progranulin, CSF progranulin were run in duplicate. Based on the standard practices of the CRO, each specimen’s final concentration (an average of the two duplicate measures) was only reported and included in our analysis if the coefficient of variance (CV) was under 20%.

© 2021 Ljubenkov PA et al. *JAMA Network Open.*

All other CSF biomarkers were measures by an additional contract research organization (CRO), PRA Health Sciences, using immunoassay techniques.

FDG-PET PET Collection

The characteristics of differing PET scanners by site are detailed in **Supplemental table 1.** The 18F-FDG-PET scan was obtained according to acceptable procedure guidelines and local hospital standards and the 18F-FDG was administered as appropriate for a radiopharmaceutical[1]. Participant preparation included the following: participants were allowed to consume any food or sugar for at least 6 hours before injection of 18F-FDG and adequate pre-hydration was ensured. A finger prick to measure glucose levels was performed prior to the 18F-FDG-PET scan. FDG-PET were uploaded in DICOM format to a secure, central server within approximately 24 hours after the scan. The uploaded FDG-PET scan was not allowed to be copied and could only be viewed by the blinded, external, FDG-PET scan reviewers or Sponsor-designated representatives during the duration of the study. The external reviewer assessed the standardization and quality of the scans.

FDG-PET pre-processing

FDG-PET images were received in the DICOM format and converted to NiFTI with in-house scripts. Each scan was warped to the Montreal Neurological Institute (MNI) standard space with SPM12 (https://www.fil.ion.ucl.ac.uk/spm/software/spm12/) with a PET-only pipeline, using the PET template provided built-in with SPM12. After warping, for each scan, the average FDG-PET uptake in the pons was extracted and used to rescale the images obtaining parametric FDG-PET Standardized Uptake Value Ratio (SUVR) images. The pons region of interest was defined according to the Automatic Anatomical Labeling atlas in MNI space as provided by the Wake Forest University WFUPickAtlas SPM12 toolbox, and was smoothed to PET resolution prior extraction of the value. Lastly, being the data acquired with different PET scanners across different centers, the final warped FDG-PET SUVR images were downsampled to match the scan with the least spatial resolution estimated using Analysis of Functional NeuroImages (AFNI) software. A total of N=52 age-matched (mean±sd age 58.9±6.7. range 47-70) cognitively-normal (CN) subjects from the Berkeley Aging Cohort Study were added for FDG-PET comparisons. FDG-PET acquisition was performed at the Lawrence Berkeley National Lab as described elsewhere[2]. CN FDG-PET scans were processed following the same pipeline described above to obtain FDG-PET SUVR images matching final resolution of patients' scans.

W-score images creation. FDG-PET W-score images (W-maps) were estimated voxelwise for individual patients correcting for age. W-score maps generation and analysis is described in detail elsewhere[3,4]. Briefly, w-scores distribution is analogous to

© 2021 Ljubenkov PA et al. *JAMA Network Open.*

z-scores distribution, representing a statistical deviation of the observed value compared to expected value based on the control group, controlling for covariates. Significance for W-scores can be assessed using gaussian curve properties. Prior to further evaluation and processing, W-maps were sign inverted in order to indicate that higher W-scores equal more severe FDG-PET hypometabolism. Group-level average W-maps were estimated across all the mutation carriers and separately according to symptomatic or presymptomatic status. Individual W-maps were qualitatively evaluated to assess patterns of FDG-PET hypometabolism and heterogeneity across single-subjects. Subsequently, W-maps were binarized using four different thresholds W>1.28, >1.64, >2.32, >3.1, corresponding to p<0.1, p<0.05, p<0.01 and p<0.001 and then summed with SPM12 to obtain voxelwise frequency maps of hypometabolism across symptomatic and presymptomatic mutation carriers.

ROI analysis. ROI analysis was performed using regional definitions from the Neuromorphometrics Atlas provided in SPM12 (Neuromorphometrics Inc, http://www.neuromorphometrics.com, provided under academic subscription), including left and right inferior, middle and superior temporal gyri, temporal pole, anterior, lateral, medial and posterior orbital gyri, middle and superior frontal gyri, orbital and triangular part of the inferior frontal gyri and frontal pole. Weighted averages of individual ROIs were then estimated to obtain for each subject lateralized FDG-PET SUVR value for temporal and frontal macroROIs. Lastly, frontal and temporal asymmetry scores were calculated with the following formula: $(SUVR_{left}-SUVR_{right})/SUVR_{bilateral}$.

Voxel wise Analysis of SUVR

Associations between FDG-SUVR and variables of interest were assessed in two separate multiple regression models, (one for NfL, one for CDR® plus NACC FTLD sum of boxes score with age and sex entered as covariates. Analyses were restricted to a grey matter mask derived from SPM tissue probability maps. Resulting T-maps were thresholded (based on uncorrected p<0.001 at the voxel level with family wise error-corrected p<0.05 at the cluster level) and converted to R-maps using the CAT12 toolbox (www.neuro.uni-jena.de/cat/). Maps were rendered on a 3D brain surface using BrainNet Viewer (www.nitrc.org/projects/bnv/) and default interpolation.

Longitudinal Analysis of SUVR

Follow-up scans were co-registered to the respective baseline images and were warped to MNI space using the respective transformation parameters. Follow-up FDG-PET SUVR images creation and ROI analyses were performed in MNI space as described for the baseline data

MRI Processing

The characteristics of differing MRIs scanners by site are detailed in **Supplemental table 1.** Before processing, all T1-weighted images were visually inspected for quality

© 2021 Ljubenkov PA et al. *JAMA Network Open.*

control. Images with excessive motion or image artifact were excluded. Tissue segmentation was performed using unified segmentation in SPM12[5]. Each subject's gray matter segmentation was warped to create a study-specific template using Diffeomorphic Anatomical Registration using Exponentiated Lie algebra (DARTEL)[6]. Subject's native space gray and white matter segmentations were then normalized and modulated to study-specific template space using nonlinear and rigid-body transformation. Images were smoothed using a Gaussian kernel of 4-mm full width half maximum. Each subject's segmentation was carefully inspected to ensure robustness of the process.

For statistical purposes, linear and nonlinear transformations between DARTEL's space and ICBM space were applied[7]. Quantification of volumes in specific brain regions was accomplished by transforming a standard parcellation atlas into ICBM space and summing all modulated gray matter within each parcellated region of interest (ROI)[8]. Total intracranial volume was calculated for each subject as the sum of the gray matter, white matter, and cerebrospinal fluid segmentations.

Original Statistical Analysis Plan (excerpt from original trial protocol)

Statistical analyses will be conducted for safety, PD, PK, and other data using appropriate methods. A detailed statistical analysis plan (SAP) will be prepared for the final analyses.

Descriptive statistics will be presented for all analyses unless otherwise specified. For categorical variables, summary tabulations of the number and percentage within each category (with a category for missing data, if applicable) of the parameter will be presented. For continuous variables, data will be presented as number (n), mean, median, standard deviation, minimum, and maximum. Least squares (LS) means and geometric means will be provided for the appropriate tables.

Unless specified otherwise, all hypothesis testing will be 1-sided at an alpha level of 0.05, with no adjustments made for multiplicity. Missing data will not be imputed, unless specified otherwise for specific analyses. Statistical analyses will be performed using SAS® software version 9.3 or higher.

Sample Size Determination
A sample size of 15 subjects per sequential period (Group 1: 300 mg or placebo; Group 2: 500 mg or placebo) (n=12 FRM-0334 and n=3 placebo) will provide approximately 80% power to detect a 30% increase in plasma PGRN concentration (based on a 1-sided test, alpha=0.05).

© 2021 Ljubenkov PA et al. *JAMA Network Open.*

eReferences

1. Boellaard R, Delgado-Bolton R, Oyen WJG, et al. FDG PET/CT: EANM procedure guidelines for tumour imaging: version 2.0. *Eur J Nucl Med Mol Imaging*. 2015;42(2):328-354. doi:10.1007/s00259-014-2961-x
2. Ossenkoppele R, Schonhaut DR, Schöll M, et al. Tau PET patterns mirror clinical and neuroanatomical variability in Alzheimer ' s disease. *Brain*. 2016;139:1551-1567. doi:10.1093/brain/aww027
3. La Joie R, Perrotin A, Barré L, et al. Region-specific hierarchy between atrophy, hypometabolism, and 2-amyloid (Aβ) load in Alzheimer's disease dementia. *J Neurosci*. 2012;32(46):16265-16273. doi:10.1523/JNEUROSCI.2170-12.2012
4. van Loenhoud AC, Wink AM, Groot C, et al. A neuroimaging approach to capture cognitive reserve: Application to Alzheimer's disease. *Hum Brain Mapp*. 2017;38(9):4703-4715. doi:10.1002/hbm.23695
5. Ashburner J, Friston KJ. Unified segmentation. *Neuroimage*. 2005;26(3):839-851. doi:10.1016/j.neuroimage.2005.02.018
6. Ashburner J. A fast diffeomorphic image registration algorithm. *Neuroimage*. 2007;38(1):95-113. doi:10.1016/j.neuroimage.2007.07.007
7. Mazziotta JC, Toga AW, Evans A, Fox P, Lancaster J. A probabilistic atlas of the human brain: theory and rationale for its development. The International Consortium for Brain Mapping (ICBM). *Neuroimage*. 1995;2(2):89-101.
8. Bakker R, Tiesinga P, Kötter R. The Scalable Brain Atlas: Instant Web-Based Access to Public Brain Atlases and Related Content. *Neuroinformatics*. 2015;13(3):353-366. doi:10.1007/s12021-014-9258-x

© 2021 Ljubenkov PA et al. *JAMA Network Open.*
