# Supplementary material for: Effect of the Histone Deacetylase Inhibitor FRM-0334 on Progranulin Levels in Patients With Progranulin Gene Haploinsufficiency: A Randomized Clinical Trial
Source: JAMA Netw Open. 2021 Sep 24;4(9):e2125584. doi: 10.1001/jamanetworkopen.2021.25584 (PMC8463943; doi:10.1001/jamanetworkopen.2021.25584)
Supplement: Supplement 3. — Data Sharing Statement [file jamanetwopen-e2125584-s003.pdf]

Data Sharing Statement

Ljubenkov. Effect of the Histone Deacetylase Inhibitor FRM-0334 on Progranulin Levels in Patients With Progranulin Gene Haploinsufficiency. *JAMA Netw Open*. Published September 24, 2021. doi:10.1001/jamanetworkopen.2021.25584

Data
Data available: No

Additional Information
Explanation for why data not available: Secondary study data may be made available in response to written requests directed to the corresponding author, at the discretion of trial investigators. Patient identifying information (including imaging data that may enable facial recognition), post-treatment data, and data pertaining to treatment assignments will not be shared outside of the clinical trial's investigators.
